# Dormancy regulon reduction was pivotal to the evolution of *Mycobacterium tuberculosis*

**DOI:** 10.1038/s41467-026-71566-x

**Published:** 2026-04-13

**Authors:** Matthew Silcocks, James P. Lingford, Chen-Yi Cheung, William J. Jowsey, Rita M. McCall, Christopher D. Rae, Liam K. Harold, David Edwards, Evan Pepper-Tunick, Stephanie L. Neville, Megan J. Maher, Aleix Canalda-Baltrons, Xuling Chang, Phan Vuong Khac Thai, Kathryn E. Holt, Nitin S. Baliga, Gregory M. Cook, Thomas R. Hawn, Nguyen Thuy Thuong Thuong, Maxine Caws, Chris Greening, Christopher A. McDevitt, Jeffery S. Cox, Matthew B. McNeil, Sarah J. Dunstan

**Affiliations:** 1 Department of Infectious Diseases, The University of Melbourne at the Peter Doherty Institute for Infection and Immunity, Parkville, VIC Australia; 2 Department of Microbiology, Biomedicine Discovery Institute, Monash University, Clayton, VIC Australia; 3 Department of Microbiology and Immunology, University of Otago, Dunedin, New Zealand; 4 Department of Molecular and Cell Biology, University of California Berkeley, Berkeley, CA USA; 5 Department of Infectious Diseases, School of Translational Medicine, Monash University, Melbourne, VIC Australia; 6 Institute for Systems Biology, Seattle, WA USA; 7 Department of Microbiology and Immunology, The Peter Doherty Institute for Infection and Immunity, The University of Melbourne, Melbourne, VIC Australia; 8 School of Chemistry, Department of Biochemistry and Pharmacology and the Bio21 Molecular Science and Biotechnology Institute, The University of Melbourne, Melbourne, VIC Australia; 9 Yong Loo Lin School of Medicine, National University of Singapore, Singapore, Singapore; 10 Khoo Teck Puat – National University Children’s Medical Institute, National University Health System, Singapore, Singapore; 11 Tam Tri Sai Gon Hospital, District 12, Ho Chi Minh City, Vietnam; 12 Department of Infection Biology, London School of Hygiene & Tropical Medicine, London, UK; 13 School of Biomedical Sciences, Queensland University of Technology, Brisbane, QLD Australia; 14 Department of Medicine, University of Washington, Seattle, WA USA; 15 Oxford University Clinical Research Unit, Hospital for Tropical Diseases, District 5, Ho Chi Minh City, Vietnam; 16 Centre for Tropical Medicine and Global Health, University of Oxford, Oxford, UK; 17 Liverpool School of Tropical Medicine, Liverpool, UK; 18 Birat Nepal Medical Trust, Kathmandu, Nepal; 19 Department of Biochemistry, University of Otago, Dunedin, New Zealand

**Keywords:** Evolutionary genetics, Population genetics, Phylogenetics, Bacterial genetics, Tuberculosis

## Abstract

Phenotypically agnostic screens for positive selection in pathogen populations provide a means of pinpointing genes and regulatory regions involved in adaptation to the local environment or host population. We screened a large (*n* = 2506) collection of Vietnamese *Mycobacterium tuberculosis* (*Mtb*) isolates, finding targets of selection to be lineage-specific, and encompass diverse functions, including dormancy (*Rv0080*), zinc homeostasis (*zur*), and virulence (ESX-1 structure). Extending our screen to the wider *Mtb* complex (MTBC) phylogeny demonstrated *Rv0080* to display an extraordinarily dynamic evolutionary history, acquiring premature stop codons or putative functional mutations on branches upstream of 8 of the 10 human-adapted lineages, and undergoing positive selection in the remaining 2. Lineage 1, which is one of two such lineages retaining the ancestral *Rv0080* sequence, displays a rate of selection for this gene (dN/dS=9.37) exceeding any other in the *Mtb* genome, save a transcription factor linked to its expression (*Rv0042c*; dN/dS=11.02). Deletion of *Rv0080*’s *M. smegmatis* orthologue confers a survival advantage in hypoxic conditions, as does the evolution of nonsense or missense mutations on an ancestral *Rv0080* background. We show the dormancy survival regulon experienced recurrent episodes of reductive evolution across the MTBC phylogeny, illuminating a novel mechanism via which it adapted to human populations.

## Abstract (web summary)

*Mycobacterium tuberculosis* owes its success partly to its ability to enter a ‘dormant’, non-replicative state, reactivating years or even decades after initial infection. In this work, authors find that a key alteration in a gene involved in this dormancy response has evolved, or is evolving, in parallel in human-adapted lineages across the globe.

## Introduction

Within-species screens for positive selection in pathogen populations^1,2^ have enabled the identification of genomic regions important for adaptation, and which can subsequently be linked to phenotypes including virulence and antibiotic resistance. In the context of *Mtb*, these screens have highlighted the role of two-component systems^3^, identified genes implicated in second-line drug resistance^3^, a shortened ‘post-antibiotic’ effect^4^, and the evolution of virulence^5^. These prior studies, however, have typically screened for positive selection in large, amalgamated cohorts of *Mtb* isolates covering diverse geographical regions, and diverse selections of lineages^3–7^. Studies investigating signatures of selection specific to *Mtb* lin.eages or human host populations are scarce^8^, or have only surveyed certain forms of positive selection, such as homoplasy^9,10^. A growing body of evidence emphasises the differing evolutionary histories^11^, geographic distributions^12^ and human host population preferences^13^ of the 11 known *Mtb* lineages^14^, thus justifying the need to consider lineages as discrete units in these selective screens.

To help explain the differences in phenotypic manifestations between lineages, we recently proposed patterns of East Asian *Mtb* diversity to reflect the ‘Two Layer’ admixture model^15^. Under this model, *Mtb* lineage 1 was carried via the initial dispersal of hunter-gatherers around the rim of the Indian Ocean^16^, and lineage 2 via the second layer of peopling, which expanded dramatically with the development of agriculture^16^. Our model, proposing a Paleolithic origin for the association of humans and *Mtb* and coexpansion during the Neolithic, provides a theoretical explanation for long-standing observations that ‘evolutionarily ancient’ lineages (lineages 1, 5, 6, 7, 8, 9 and 10) display lower levels of virulence and are more likely to result in latent infection than their ‘modern’ counterparts (lineages 2, 3 and 4)^17–19^. Thus far, however, a genetic explanation for the difference between ancient and modern phenotypes remains elusive.

Here we screen for genes under positive selection amongst *Mtb* isolates from Ho Chi Minh City (HCMC), Vietnam: a setting allowing us to compare the evolutionary trajectories of ancient (lineage 1) and modern (lineages 2 and 4) lineages on a homogenous host genetic background. We posit that identifying targets of selection specific to ancient or modern lineages may help identify the genetic factors underpinning these two phenotypes. Intriguingly, we found a dormancy regulon gene, *Rv0080*, to be under intense positive selection in lineage 1, and initially hypothesised this to indicate selection against the dormancy response typical of ancient lineages. Analysis of the broader *Mtb* phylogeny, however, showed a loss of *Rv0080* function to have arisen repeatedly across the deep evolutionary history of the MTBC, thus revealing a key feature in the bigger picture of *Mtb* evolution.

## Results

### Positive selection amongst *Mtb* isolates in HCMC, Vietnam

**Fig. 1:**
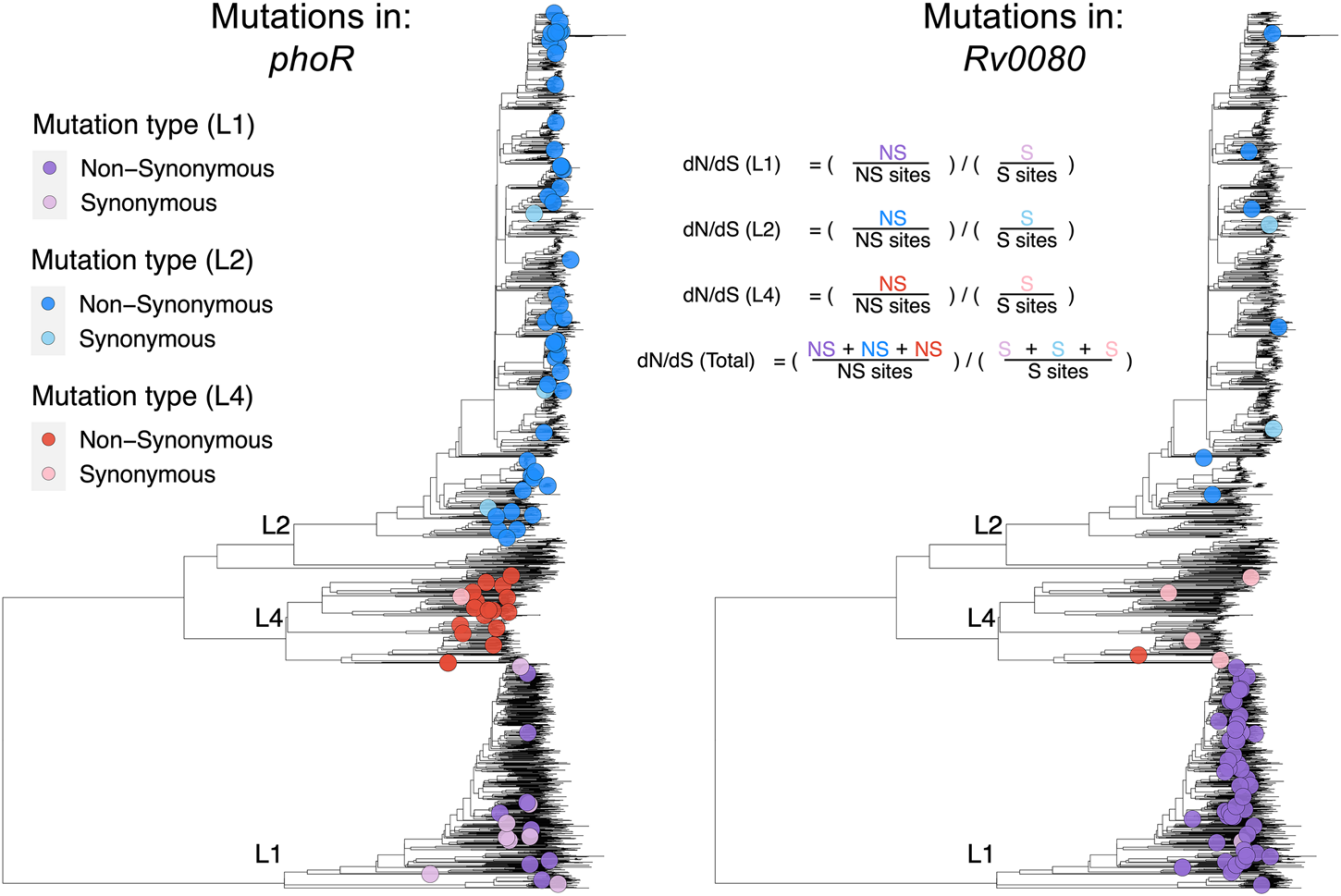
Approach used to calculate per-lineage and total dN/dS values for two genes in the *Mtb* genome. These two genes (left: *phoR* and right: *Rv0080*) represent strong examples of lineage-specific positive selection. *phoR* undergoes strong positive selection in lineage 2 (L2) and lineage 4 (L4), while *Rv0080* undergoes strong positive selection in lineage 1 (L1). Non-synonymous (NS; dark points) and synonymous (S; light points) mutations for a given gene are inferred across the branches of the Vietnamese *Mtb* phylogeny for L1 (*n* = 643 isolates), L2 (*n* = 1589) and L4 (*n* = 271). Per lineage dN/dS ratios for a given gene are calculated by summing NS and S mutations across each lineage, dividing each by the number of potential NS or S mutations per gene (also referred to as the number of NS or S sites), and taking the ratio (equations given in right panel). An overall dN/dS score per gene is also produced using sums of NS and S mutations across all lineages.

We inferred a phylogeny of 2506 *Mtb* isolates from HCMC, Vietnam, after calling variants using best-practice approaches, and restricting calls to regions amenable to short read mapping^20^. We then performed ancestral sequence reconstruction^21^ to infer the distribution of mutations across the evolutionary history of these isolates. We used an approach that allowed us to distinguish between non-synonymous and synonymous mutations and produced tallies of the total number of these two classes of mutation per gene^22^. By incorporating the total number of synonymous and non-synonymous sites within all non-masked regions of each gene, we calculated dN/dS ratios, which act as a quantitative measure of the strength of natural selection operating per gene. dN/dS values were calculated when considering the combined sum of non-synonymous and synonymous mutations in all lineages, and when considering each of the three lineages (i.e. lineage, 1, 2 and 4) separately (see equations in Fig. 1). This approach allowed us to identify instances of lineage-specific positive selection, which proved to be a key theme of this screen (Fig. 1).

**Fig. 2:**
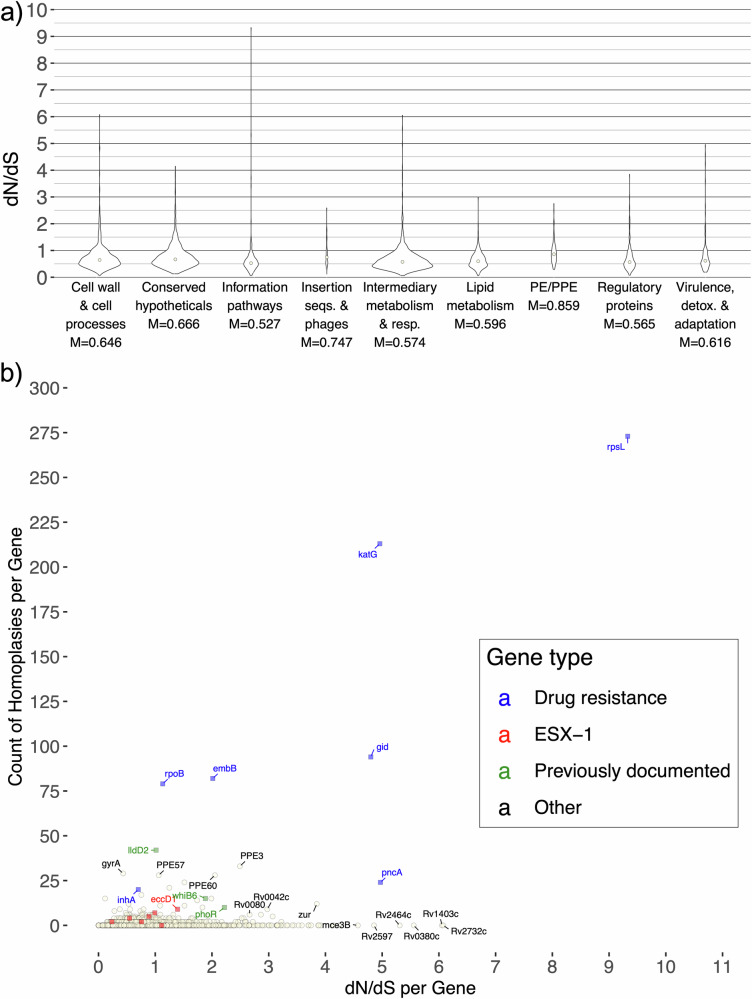
Quantifying the rate of positive selection across *Mtb* genes and functional categories. **a** Distributions of the rates of positive selection (measured as dN/dS) for genes in each of nine functional categories (defined according to Mycobrowser, release 4). Rates were inferred across all lineages of the Vietnamese *Mtb* dataset. Points indicate median values for each distribution, and labels below each distribution list these values. **b** Scatterplot of the rate of dN/dS per gene against the count of homoplastic mutations within that gene across all *Mtb* lineages. Genes implicated in first line drug resistance (blue), ESX-1 structural elements (red) and those identified in prior screens for positive selection (green) are marked for emphasis.

The median dN/dS value per gene across all lineages was 0.611, which was similar to previous within-species estimates for *Mtb*^3^. Comparing rates of dN/dS across different gene functional categories (defined according to Mycobrowser^23^, release 4) revealed significant differences (Kruskal–Wallis test *p* < 2.2 × 10^−16^), with ‘PE/PPE genes’ possessing the highest median rates of positive selection (dN/dS = 0.859), and genes involved in ‘information pathways’ (dN/dS = 0.527) and ‘regulatory proteins’ (dN/dS = 0.565) the lowest (Fig. 2a). Unsurprisingly, genes annotated as essential were more strongly conserved, on average, than genes annotated as non-essential (median dN/dS = 0.494 vs 0.643; Mann–Whitney U test *p* < 2.2 × 10^−16^).

We contrasted gene-level dN/dS ratios with the counts of individual homoplasies (identical amino acid changes occurring independently across the phylogeny) within that gene to identify genes displaying convergent evolution at specific sites. The genes with the highest count of individual homoplasies included the drug resistance-associated genes *katG*, *rpsL*, *gid*, *rpoB*, *embB* and *pncA*, as well as putative virulence genes reported in previous screens for selection, such as *phoR*^4,5^, *lldD2*^10,24^ and *whiB6*^4^. Other genes with high rates of homoplasy included structural elements of the ESX-1 locus, which includes *eccD1*, *eccCa1*, *eccCb1*, *eccB1*, *eccE1*, *eccA1* and *mycP1* (Fig. 2b; red points), which carry up to nine homoplasies each, and a total of 29 homoplasies collectively. This signal of selection in ESX-1 genes was largely driven by isolates from lineage 1. Lineage 1 isolates accumulated 5, 4, 3, 3 and 2 lineage-specific homoplasies in each of *eccD1*, *eccCb1*, *eccCa1*, *mycP1* and *eccE1*, respectively, while no lineage-specific homoplasies were observed amongst isolates from lineages 2 or 4. We also found the previously documented signal of selection in *phoR* to be specific to modern lineages 2 (6 homoplasies; dN/dS = 5.71) and 4 (2 homoplasies; dN/dS = 6.47), and not ancient lineage 1 (0 homoplasies; dN/dS = 0.380). An additional class of gene which stood out were members of the PE/PPE family, particularly *PPE3*, *PPE60* and *PPE57* (Fig. 2b). Although we make use of an approach which allows more reliable variant calling within PE/PPE genes^20^, we remain cautious of inferred rates of selection, given the homologous nature of PE/PPE genes.

We also completed an independent screen, aiming to identify genes that are globally conserved (i.e. gene-level dN/dS <1), yet subject to positive selection at a subset of sites. Using PAML software, we calculated both the proportion of sites in each gene experiencing, purifying selection (dN/dS < 1), positive selection (dN/dS > 1) and neutral evolution (dN/dS = 1), and the proportion of sites with posterior probabilities of dN/dS > 1 above various thresholds (thus identifying specific sites showing strong evidence of selection). Consistent with Wilson et al.^7^, only a small proportion of sites (range: 0–0.129) in each gene display posterior probabilities of selection greater than 0.9 (Supplementary Fig. 1). An example of a gene displaying evidence of purifying selection overall (dN/dS = 0.344), yet a relatively high proportion of sites under selection (proportion >0.9 of 7.59 × 10^−3^%) was *prpD* (Supplementary Fig. 2). A total of 4 residues in *prpD* had a posterior probability of positive selection of 0.9 or higher (thus indicating compelling evidence for selection), each of which evolved multiple mutations in the Vietnamese dataset. This screen also proved useful for highlighting other putative drug resistance targets, including *Rv1129c*^25^ (>0.9 of 8.21 × 10^−3^%; dN/dS=1.08) and *dnaA*^26^ (>0.9 of 5.91 × 10^−3^%; dN/dS = 1.08), possibly reflecting selection for specific amino acid substitutions on an otherwise conserved gene background. When considering other gene hits, a gene displaying a particularly low dN/dS ratio (dN/dS = 0.149), yet still experiencing site-specific positive selection was *leuA*: an essential element of the leucine biosynthesis pathway (Supplementary Fig. 2). A site of strong interest within *leuA* was residue 391, which underwent 9 independent mutations across the Vietnamese dataset, indicating strong selection for a subtle alteration in this otherwise highly conserved gene. Future genomic screens and functional follow-up studies may benefit from the prioritisation of genes displaying a similar pattern of selection, which evade typical screening filters.

Returning to our initial screen, and when comparing lineages 1 and 2, the two most numerous lineages in our dataset, we documented significant differences in the rate of positive selection for genes involved in ‘information pathways’ (paired Wilcoxon test for genes with more than 10 mutations in both lineages; *p* = 3.62 × 10^−3^), ‘intermediary metabolism and respiration’ (*p* = 4.31 × 10^−4^) and ‘lipid metabolism’ (*p* = 4.72 × 10^−3^). Consistent with prior investigations^9^, we found the median dN/dS rate per gene to be higher for lineage 2 than for lineage 1 (0.656 vs 0.583, Mann–Whitney U test *p* = 1.47 × 10^−10^). These observations motivated our exploration of selection at the lineage level, particularly the genes showing strong differences in the rate of positive selection between lineages (lineage-specific selection). We focused on the three most prominent instances of lineage-specific positive selection in the *Mtb* genome: *Rv0080*, *Rv0042c* and *zur* (Supplementary Fig. 3). All three of these genes are under intense positive selection in lineage 1 (dN/dS > 7.5; Supplementary Fig. 3), and none have been characterised in prior selective screens.

### A dormancy survival regulon gene is under strong selection in lineage 1 genomes

One of the genes displaying the highest rate of positive selection across our *Mtb* dataset was *Rv0080*, which forms part of the 48 gene dormancy survival (*dos*) regulon^27,28^. Genes of the *dos* regulon are expressed during the transition of the *Mtb* bacteria to what is generally considered to be a non-replicative, ‘dormant’ state, including in response to hypoxia, and facilitate survival throughout the dormancy period. While several members of the *dos* regulon have been functionally characterised^29–31^, the role of many, including *Rv0080*, remains unknown.

**Fig. 3:**
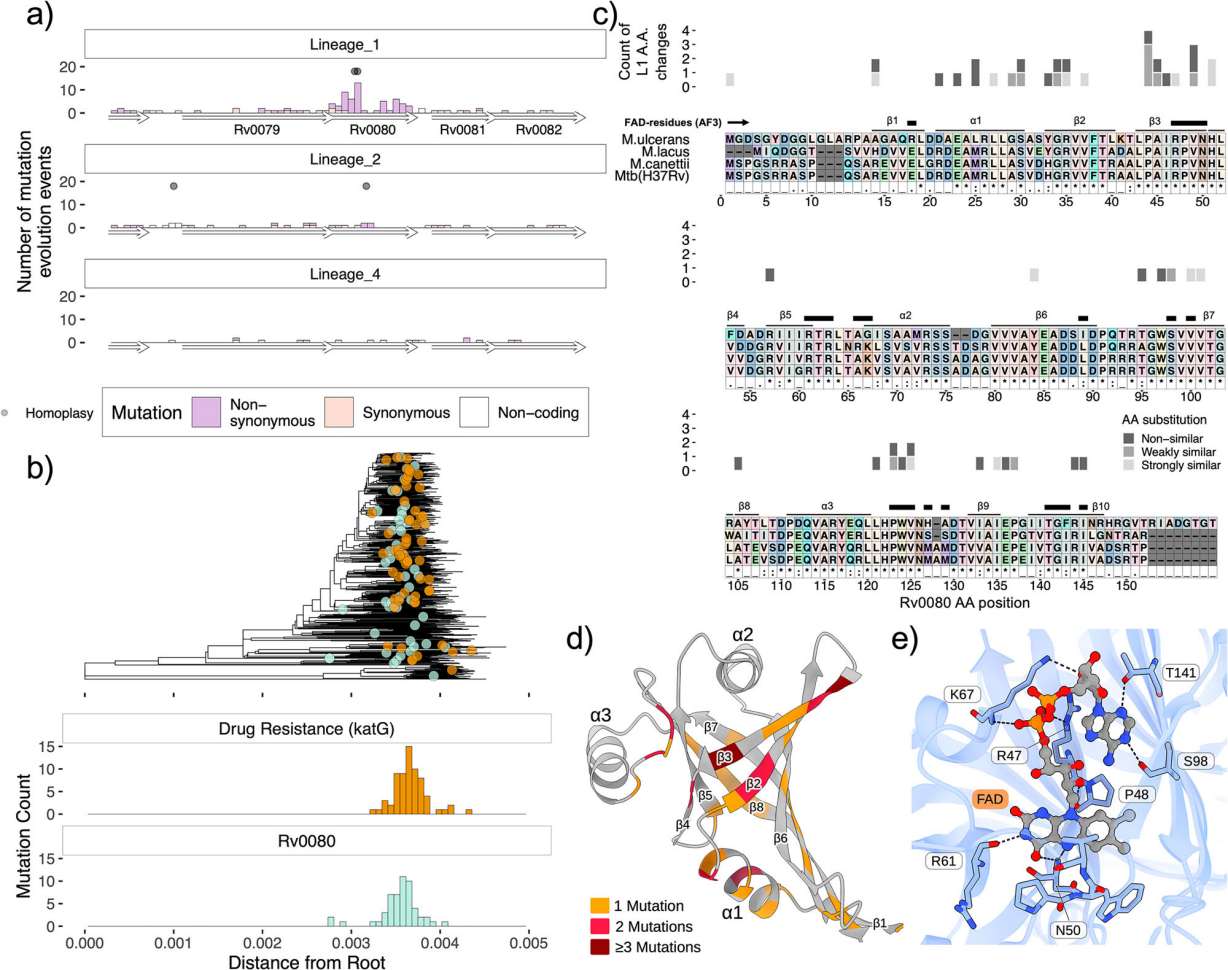
Selection for *Rv0080* mutations amongst lineage 1 strains and their structural context. **a** Histogram showing the count of non-synonymous, synonymous and non-coding mutations across *Rv0080* and surrounding genes for lineages 1, 2 and 4. Grey points above each histogram show the locations of lineage-specific homoplasies. **b** (Upper) phylogeny of lineage 1 isolates with non-synonymous mutations in *katG* (orange) and *Rv0080* (light blue) marked as points on the branches on which they were inferred to occur. (Lower) histograms showing the distribution of the distance of these *katG* and *Rv0080* mutations from the root of the tree. **c** Alignment of *Rv0080* and its mycobacterial homologues, with histogram showing the distribution of amino acid alterations across all Vietnamese lineage 1 isolates. Symbols below each residue in the alignment indicate the degree of conservation of that residue across homologues (‘*’ designates fully conserved residues, ‘:’ strongly conserved, ‘.’ weakly conserved and ‘_’ non-conserved). Colour coding of each cell of the histogram indicates the type of amino acid change for each lineage 1 mutation. Thick black bars above the amino acid sequence indicate residues within the predicted FAD-binding region. Thin black bars indicate elements of the *Rv0080* secondary structure. **d** AlphaFold3 model of a single chain of *Rv0080* with secondary structure elements labelled, and mutations occurring amongst Vietnamese isolates annotated in a colour scale (orange = 1 mutation; red = 2 mutations; dark red = 3 or more mutations). **e** Close-up view of the AlphaFold3 predicted FAD-binding site, with FAD-interacting residues shown.

The signal of positive selection observed for *Rv0080* was entirely driven by isolates from lineage 1. Across the lineage 1 tree, we observed 51 NS mutations in the *Rv0080* gene, and only 2 S mutations (Fig. 3a; dN/dS = 9.37). In contrast, only 6 NS mutations were found across the L2 tree and 2 S mutations, and 1 NS and 4 S mutations were found across the L4 tree (dN/dS rates not measured due to <10 mutations per gene). To put these figures in context, the proportion of lineage 1 isolates that carry a non-synonymous mutation in the *Rv0080* gene (78/643; 12.1%), exceeds the proportion carrying mutations in *katG* (63/643; 9.80%), the strongest target for isoniazid resistance in the *Mtb* genome. *Rv0080* was also enriched for homoplasies in lineage 1 relative to other lineages (4 NS homoplasies in L1 vs 2 and 0 in L2 and L4, respectively). We investigated the possibility that these rates of homoplasy could have arisen by chance, by applying a permutation test that counts homoplasies across randomly sampled blocks of the *Mtb* genome. The rate observed for lineage 1 was significantly higher than expected by chance when applying this permutation test (*p* = 1 × 10^−3^). When relaxing the definition of ‘homoplasy’ to consider all possible amino acid changes at a specific site (i.e. counting all mutations in residues with more than 1 mutation in Fig. 3c), the number of L1 homoplasies rose to 25, yet stayed the same for lineages 2 and 4 (2 and 0, respectively).

As the strongest signals of positive selection in *Mtb* cohorts generally reflect forms of adaptation to antibiotic resistance, we devised a series of tests to rule out the involvement of our genes of interest in these resistance pathways. These tests incorporated antibiotic resistance phenotype data, and considered the depth and ordering of mutations with respect to those in known antibiotic resistance genes. Three lines of evidence suggested that *Rv0080* is unlikely to play a role in the evolution of drug resistance. Firstly, we found *Rv0080* mutations to be older (situated closer to the root of the tree), on average, than mutations in genes associated with the first anti-TB drugs introduced in Vietnam: *katG* (isoniazid; INH) and *gid* (streptomycin; STR) (Fig. 3b; one sided Mann–Whitney U test; *p* = 1.59 × 10^−3^ for *katG* and *p* = 9.69 × 10^−4^ for *gid*). Secondly, by inspecting paths through the phylogeny where both *Rv0080* and *katG* mutations co-occur (9 in total), we find the *Rv0080* mutations to have arisen first on 5 instances, and second on 0 (the remaining 4 *Rv0080*-*katG* mutation pairs occurred on the same branch). Thirdly, we did not detect an enrichment of *Rv0080* mutations in isolates with phenotypic resistance to either INH or STR (chi squared test; *p* = 0.376 and *p* = 0.734, respectively).

Next, we considered the distribution of *Rv0080* mutations with respect to its amino acid sequence and that of closely related homologues (Fig. 3c). In interpreting the potential effects of mutations in the amino acid sequence of *Rv0080*, we made use of principles of residue conservation, as applied in the context of *Mtb* by Hershberg et al.^32^. We found 80.0% of mutations occurring in the *Rv0080* gene amongst lineage 1 isolates are in residues that are conserved in an alignment of homologues: a significantly higher figure than expected if these mutations were distributed randomly (chi squared test, *p* = 4.88 × 10^−4^). We also found 52.0% of *Rv0080* amino acid changes in lineage 1 to result in residues classified as ‘non-similar’: a higher figure than expected by chance when considering genome-wide patterns (permutation test; *p* = 0.012). The results both support the conclusion that the mutations observed in *Rv0080* amongst lineage 1 isolates are likely to have a strong effect on the structure or function of the protein, possibly resulting in a loss of function.

We next predicted the structure and function of *Rv0080* to help formulate hypotheses about its biological role. *Rv0080* belongs to the flavin/deazaflavin-dependent oxidoreductase (FDOR) superfamily, which fulfill a diverse range of functions using various redox cofactors^33^. A prior study of this protein superfamily indicated that *Rv0080* belongs to a subgroup predicted to bind flavin adenine dinucleotide (FAD) as a cofactor^33^. *Rv0080*’s only characterized homologue is *MSMEG_5243* from *M. smegmatis* (33.80% amino acid identity), which functions as a flavin-sequestering protein (renamed Fsq) that protects FAD from autooxidation and ROS production during hypoxia^34^. *fsq* is also a dormancy regulon member, and mutants defective in *fsq* are hypersensitive to oxidative stress when recovering from hypoxia^34^. We used AlphaFold to predict the *Rv0080* protein structure in both its apo (unbound) and FAD-bound forms (Supplementary Figs. 4, 5b, c). AlphaFold2 (AF2) and AlphaFold3 (AF3) both predict *Rv0080* to form a homodimer with high structural similarity to Fsq (Supplementary Fig. 5a; root mean square deviation of 0.862 Å and 0.888 Å respectively). *Rv0080* is predicted to bind FAD, forming hydrogen-bonds and hydrophobic contacts with both chains of the *Rv0080* homodimer interface (Fig. 5e and Supplementary Fig. 5d). *Rv0080*’s predicted FAD-binding residues were highly concordant with FAD-binding sites in the Fsq crystal structure (Supplementary Figs. 6, 5e).

Using the predicted *Rv0080* structures, we analysed the distribution of mutations amongst Vietnamese L1 isolates, revealing a significant enrichment in residues within the predicted FAD-binding region (mean of 0.545 mutations per residue in FAD-binding region and 0.264 outside; Mann–Whitney U test, *p* = 0.0283; Fig. 3c). While ablating FAD binding is likely a role of this subset of mutations, many also occurred outside the FAD-binding region (Fig. 3c, d), and may alter the function of *Rv0080* through other means, such as destabilising the overall protein structure. To investigate this possibility, we assessed the impact of mutations on *Rv0080*’s thermodynamic stability by predicting changes in the Gibbs free energy of folding (ΔΔG) in silico using Rosetta^35,36^. This revealed many of the mutations that occurred outside the predicted FAD-binding region to result in high ΔΔG values (Supplementary Fig. 7a), thus indicating a decrease in stability. Consistent with their hypothesised role in ablating FAD binding, we found three of the seven mutations which resulted in negative ΔΔG values to be located in the predicted FAD-binding region, and one other (R144L) to be surrounded by FAD-binding region residues (Supplementary Fig. 7a). We found *Rv0080* mutations observed amongst Vietnamese isolates to be more destabilising, on average, than non-synonymous mutations that were randomly induced in the *Rv0080* amino acid sequence (mean ΔΔG of 1.70 vs 1.22; Mann–Whitney U test, *p* = 4.85 × 10^−3^). Using Rosetta to conduct a ‘saturation mutagenesis’ enumerating all possible amino acid substitutions in *Rv0080* revealed strong destabilising effects for mutations in alpha-helix 1, and most beta-strands (Supplementary Fig. 8), which may explain mutational hotspots in non-FAD-binding regions (i.e. α1: 8 mutations; β2: 6 mutations; Fig. 3c, d).

Since the FAD-binding pocket requires two chains of *Rv0080* forming a homodimer, we also predicted the influence of mutations on the stability of the *Rv0080* homodimer interface using Rosetta FlexDDG. This analysis may suggest the adaptive role of additional mutations, for instance the mutations W124R and V125G were predicted to have substantial impacts on dimer stability (Supplementary Fig. 7b). We also infer certain mutations to Pro44 (the most frequently mutated in our cohort) to produce low to moderate decreases to dimer stability, however refrain from making strong inferences given their relatively low magnitude (Supplementary Fig. 7b). Although we were not able to ascribe an adaptive role to all mutations observed in *Rv0080*, the tendency of mutations to result in decreased protein stability or fall within the FAD-binding region supports selection for a loss or major alteration in function of this protein.

The distribution of indels across our dataset provides evidence that a complete loss of *Rv0080* function is one of the mechanisms targeted by selection. An additional 16 lineage 1 isolates were impacted by indels within the coding region of the *Rv0080* gene. Nine of these 16 indels resulted in frameshifts and led to premature stop codons, while the remaining 7 resulted in a variety of amino acid insertions or deletions (Supplementary Fig. 9). Only a single indel within the *Rv0080* gene was observed amongst the lineage 2 and 4 isolates in our dataset.

### *Rv0080* displays a dynamic evolutionary history across human-adapted MTBC lineages

Next, we sought to characterise the evolution of *Rv0080* across a wider sampling of lineage 1 isolates, to determine whether the signal of positive selection we observe was specific to this Vietnamese *Mtb* cohort. To do this we assembled a dataset of *Mtb* isolates spanning the full geographical range of lineage 1, including India, Thailand, Cambodia, northern Vietnam, Borneo and the Philippines (Supplementary Fig. 10). This dataset, which contains 981 L1 genomes, will henceforth be referred to as the Pan-Asia *Mtb* dataset.

**Fig. 4:**
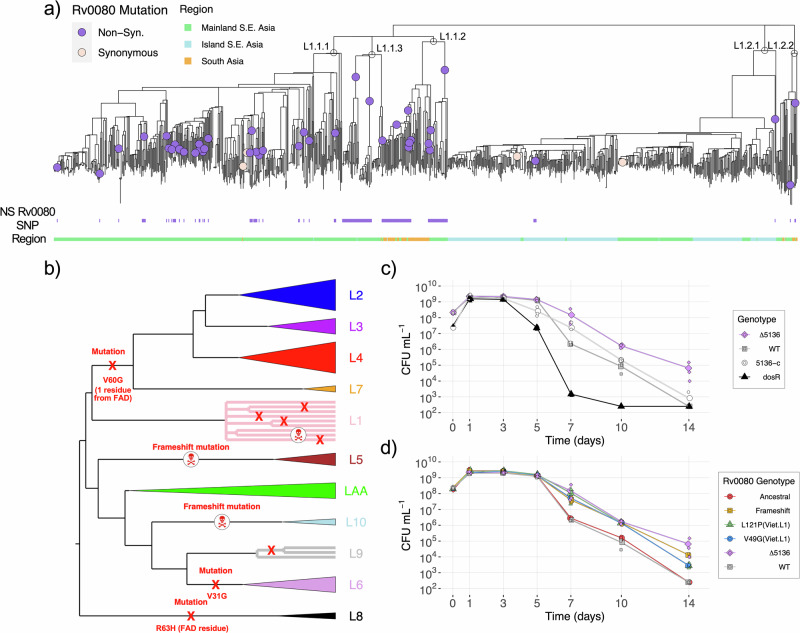
Selection for *Rv0080* mutations across the deep evolutionary history of the MTBC and their impact on hypoxia survival. **a** Phylogeny of lineage 1 isolates from the Pan-Asia *Mtb* dataset, with *Rv0080* variants marked as points on the branches on which they were inferred to occur. Pink points correspond to synonymous mutations and purple points correspond to non-synonymous mutations. The geographical region in which these isolates were identified is represented by the coloured bar below the phylogeny. **b** Phylogeny of the MTBC, showing all known lineages, and the mutations which occur within *Rv0080* on lineage-defining branches (red crosses), inferred from the LAA-Z dataset. All lineages aside from 1 and 9 were collapsed to illustrate the signal of ongoing positive selection we observe for these lineages. The label ‘LAA’ designates the main animal-adapted clade of the MTBC. **c** Survival of Δ*dosR*, Δ*MSMEG_5136*, Δ*MSMEG_5136*-Complement and the wild type *M. smegmatis* strain (measured as colony forming units) between the timepoint zero, and the completion of the hypoxia experiment (14 days). Small points indicate individual replicates (triplicate), and large points indicate the mean. **d** Survival of *M. smegmatis* Δ*MSMEG_5136* strains expressing various *Rv0080* alleles selected from amongst Vietnamese lineage 1 strains.

This analysis revealed *Rv0080* to be under positive selection in four of the five sublineages of L1 (L1.1.1, L1.1.2, L1.1.3 and L1.2.2) (Fig. 4a). The most numerous of these four sublineage, L1.1.1, evolved a total of 25 NS mutations and 2 S mutations in *Rv0080*, resulting in 10.7% (41/380) of isolates possessing NS mutations (Supplementary Table 1). Despite small sample sizes for sublineages L1.1.2 (*n* = 86), L1.1.3 (*n* = 54) and L1.2.2 (*n* = 28), we observed 9, 3 and 2 non-synonymous *Rv0080* mutation evolution events in each, and no synonymous mutations (Supplementary Table 1). Many of the mutations in L1.1.2 and L1.1.3 occurred early in the evolutionary history of these lineages, and we find 73.2% (63/86) and 75.9% (41/54) of isolates from these sublineages possess NS mutations in *Rv0080* (Fig. 4a; Supplementary Table 1). As per the Vietnamese dataset, multiple L1 isolates from the Pan-Asia *Mtb* dataset carried indels within the *Rv0080* coding sequence (9 in total), and we observed an enrichment of mutations in residues conserved across *Rv0080’*s homologues (chi squared test, *p* = 0.049) and the inferred FAD-binding region (Mann–Whitney U test, *p* = 8.45 × 10^−3^; Supplementary Fig. 11).

These results collectively suggest mutations in *Rv0080* to be a common mechanism of evolutionary adaptation amongst lineage 1 genomes. A notable exception was sublineage 1.2.1, which evolved only 2 NS mutations and 2 S mutations in *Rv0080*, leading to a total of 1.1% (5/433) of isolates possessing non-synonymous mutations (Supplementary Table 1). Strikingly, we find the two isolates descending from the first and second deepest splits of the L1.2.1 subtree, both from Borneo, carried *Rv0080* variants: the first an indel leading to a frameshift, and the second a V to A mutation at residue 100, which is within the FAD-binding region (Supplementary Fig. 12). We infer that a yet unknown mutation that occurs on the branch leading to the remaining isolates in this sublineage (marked with a blue star in Supplementary Fig. 12) obviates the need for this clade to undergo selection in *Rv0080*.

When assessing our Vietnamese *Mtb* dataset, we were surprised to find one additional isolate with an indel affecting the *Rv0080* coding sequence: the single lineage 5 isolate (not from Vietnam) used as an outgroup in phylogenetic inference. This observation motivated us to explore the evolution of *Rv0080* across the full breadth of MTBC phylogenetic diversity. To do this, we assembled a dataset henceforth referred to as the LAA-Z dataset, which contains strains representative of the deepest splits in all known human (L1-10) and animal-adapted (LAA) lineages (see Methods). We found *Rv0080* to display a highly dynamic evolutionary history across all human-adapted MTBC lineages, with frameshift mutations occurring on the lineage-defining branches of 2 of 10 lineages, non-synonymous mutations predicted to affect FAD binding or protein stability occurring on the branches upstream of 6 of 10 lineages, and ongoing positive selection occurring in the remaining 2 lineages (Fig. 4b).

Specifically, the premature stop codon-inducing indel documented in our lineage 5 outgroup sample was found to be fixed in all sublineages of the lineage 5 tree (Fig. 4b). We also observed an indel leading to a premature stop codon in both available genomes of the newly documented lineage 10 (Fig. 4b). The branch leading to all modern *Mtb* lineages (L2, 3 and 4), and lineage 7, was characterised by a V to G mutation at residue 60; which neighbours a residue predicted to forms hydrogen bonds with FAD (residue 61; Fig. 4b). Similarly, both available lineage 8 genomes carried a R to H mutation at residue 63, which is within the inferred FAD-binding region (Fig. 4b). Finally, all strains belonging to lineage 6 carried a V to G mutation at residue 31 (Fig. 4b). While not in the predicted FAD-binding region, residue 31 falls in a mutational hotspot in both the Vietnamese and Pan-Asia *Mtb* datasets, residing between alpha-helix 1 and beta-strand 2. We also observed that the two lineage-defining mutations, which did not fall directly within the inferred FAD-binding region, V60G and V31G, were predicted to have strong destabilising effects on the protein (mean ΔΔG = 4.57, s.d. = 0.186 and ΔΔG = 2.64, s.d. = 0.069 respectively), thus providing complementary evidence of their functional relevance. Consistent with observations from the Vietnamese dataset, the FAD-binding region mutation, R63H, did not result in a substantial decrease in predicted protein stability (mean ΔΔG = −0.175, s.d. = 0.418).

We aimed to contextualise the high count of amino acid-altering mutations in *Rv0080* on the lineage-defining branches of the phylogeny via comparison to mutation counts in other genes. Of the 932 genes within the 100–200 amino acid range (*Rv0080* is 152 residues), *Rv0080* was the only gene to undergo 5 or more amino acid-altering mutations (a class of mutation which includes both non-synonymous mutations and indels in coding regions). Via applying a permutation test which redistributes amino acid-altering mutations on lineage-defining branches randomly across the genome, we find *Rv0080* to accumulate significantly more mutations than expected by chance (*p* = 4 × 10^−5^). When adopting a more conservative approach to this analysis and considering only indels (which are responsible for the two frameshift mutations observed), *Rv0080* still returned a significant *p*-value (*p* = 1 × 10^−3^), being the only gene in the 100–200 amino acid range to accumulate two or more. Aside from lineage 1, which we have demonstrated to exhibit extreme positive selection for *Rv0080*, the only remaining human-adapted lineage of the MTBC without an *Rv0080* mutation on an upstream branch is lineage 9. Of the five available lineage 9 genomes, we documented an A to D mutation at residue 78 in three of them, and an L to F mutation at residue 52 in one. Thus, we conclude that *Rv0080* exhibits an extraordinary pattern of positive selection across human-adapted lineages of the MTBC, with non-synonymous, or loss of function mutations occurring on the lineage-defining branches upstream of 8 of 10 lineages, and ongoing positive selection occurring on the remaining two (Fig. 4b).

We verified that no positive selection for *Rv0080* had occurred within the animal-adapted clade (LAA) of the MTBC by screening 493 genomes (0 NS mutations and 0 S mutations). By analysing the lineage 2 (*n* = 686) and 4 (*n* = 247) genomes from the Pan-Asia *Mtb* dataset, and a collection of 277 lineage 6 genomes, we found no evidence of selection in *Rv0080* in these lineages (3 NS and 2 S mutations for L2; 3 NS and 0 S for L4; 2 NS and 0 S for L6). We also verified that all isolates from L2 and L4 in both the Vietnamese and Pan-Asia datasets possessed the V60G substitution, and that all L6 isolates analysed possessed the V31G substitution, thus confirming the absence of any back-mutations at these loci. We speculate that the lack of selection for *Rv0080* in lineages 2, 4 and 6 can be explained via the mutations they acquire on their lineage-defining branches rendering the protein largely or completely non-functional. Of relevance to our understanding of the role of *Rv0080* in modern lineages is the presence of a large clade of 22 Thai isolates from the Pan-Asia dataset that all carry a premature stop codon in *Rv0080* (Supplementary Fig. 13). The presence of a large clade with this unambiguously non-functional version of *Rv0080* implies the gene is not essential for survival or transmission on a lineage 2 background, and indicates the absence of strong purifying selection against strains possessing this mutation. Two main inferences we draw from this data are firstly, that modern lineages don’t appear to undergo selection for a total ablation of *Rv0080* function, but secondly that if such a mutation does occur, it doesn’t appear to be accompanied by a strong selective disadvantage. While functional data is required to substantiate this hypothesis, this data is consistent with the notion that the V60G substitution alters *Rv0080* function in a way that obviates the selection pressure for modern lineages to evolve additional mutations.

Still pestered by the lack of selection displayed by the L1.2.1 sublineage, we inspected mutations arising on the branch defining this clade (blue star; Supplementary Fig. 12). A candidate of interest was a NS mutation in the gene *Rv0042c*, which encodes a transcription factor, and which is the only gene to display a rate of selection higher than *Rv0080* (29 NS mutations; 1 S mutation; dN/dS = 11.02; Supplementary Fig. 3). We inspected the transcription factor overexpression data of Rustad et al.^37^, hypothesising that *Rv0042c* may be involved in the regulation of the *Rv0079*-*Rv0080* operon. Of the 206 transcription factors profiled, *Rv0042c* was found to be the highest driver of *Rv0079* expression aside from *dosR*, and the second highest driver of *Rv0080* expression aside from *dosR* and *Rv0081* (Supplementary Fig. 14). We therefore concluded that the extreme signal of L1-specific positive selection observed for *Rv0042c* (Supplementary Figs. 3, 15 may represent a mechanism of reducing or eliminating the expression of *Rv0080*. Exploring the distribution of *Rv0042c* mutations amongst L1 isolates from the Vietnamese dataset, we infer that a loss of function is one of the mechanisms targeted by selection. We found a significant enrichment of mutations in residues conserved across an alignment of *Rv0042c* orthologues (chi squared test, *p* = 0.012; Supplementary Fig. 16), and a total of 6 isolates with frameshift-inducing indels in the *Rv0042c* coding sequence. We note the hypothesis that a loss of *Rv0042c* function leads to a similar phenotype to a loss of *Rv0080* function to be directionally consistent with the data of Rustad et al.^37^, who show an increase in *Rv0042c* expression to result in an increase in the expression of *Rv0080*.

As a final means of investigating selection acting on *Rv0080*, we re-analysed the unfixed mutation data of Liu et al.^4^. This dataset reports sites in the genome for which reads supporting both the reference and alternate allele are found amongst the same isolate, thus providing insight into selection on a very recent timescale. When screening isolates belonging to all lineages (51,229 in total), we found *Rv0080* to accumulate a total of 32 mutations (28 NS and 4 S), and to rank as the gene with the 140th highest non-synonymous mutation density. When focusing our screen on lineage 1 isolates only (4762 isolates), the rank of *Rv0080* rose to 21st, with 8 NS mutations and 0 S mutations (Supplementary Fig. 17). In contrast, the NS mutation density ranked outside the top 700 genes when screening lineage 2 (13,650 isolates), and outside the top 1900 when screening lineage 4 (26,183 isolates). Of the four L1 sublineages for which *Rv0080* is under positive selection (L1.1.1, L1.1.2, L1.1.3 and L1.2.2), we found the two with the lowest proportion of fixed *Rv0080* mutations to display the highest rates of unfixed mutations (*Rv0080* ranked 8th for L1.1.1, and 55th for L1.2.2), suggesting a lack of selection pressure on genetic backgrounds which already carry an *Rv0080* mutation. This data provides evidence of the ongoing selection pressure facing *Rv0080* amongst L1 isolates to this day.

### Loss of *Rv0080* and its *M. smegmatis* orthologue confers a hypoxia survival advantage

Given the link between expression of *Rv0079*-*Rv0080* and hypoxia-related transcription factors (e.g. *dosR*), we hypothesized that *Rv0080* variants under positive selection would provide a survival advantage under hypoxic conditions. To investigate this hypothesis, we deleted *Rv0080*’s orthologue in *M. smegmatis*: *MSMEG_5136* (39.13% sequence identity; Supplementary Fig. 18a), which is also in the *dos* regulon. We compared the hypoxic survival of a markerless gene deletions of *MSMEG_5136* with wild-type mc2 155 and a markerless *dosR* deletion^38^. All strains were grown in sealed serum vials providing gradual onset of hypoxia via self-consumption of oxygen^34^. During the oxygen growth phase (first 24 h) all strains grew the same (Fig. 4c). At day 5, Δ*dosR* displayed an immediate reduction in cell survival, reaching the lower limit of detection within 10 days (Fig. 4c). Conversely, wild-type *M. smegmatis* had a gradual decline in viability reaching the lower limit of detection after 14 days (Fig. 4c). Consistent with our hypothesis that the loss of *Rv0080* provides a fitness advantage under hypoxia, the viability of Δ*MSMEG_5136* decreased at a lower rate compared to wild-type *M. smegmatis*, with ~10^5^ viable cells/ml remaining on day 14 (Fig. 4c). Furthermore, there was a 1–2 log_10_ difference in survival between Δ*MSMEG_5136* and the wild type at days 7 and 10 (Fig. 4c). Complementation of Δ*MSMEG_5136* with *MSMEG_5136* expressed from a native promoter restored a wild-type survival curve (Fig. 4c). In conclusion, the loss of the *Rv0080* orthologue, *MSMEG_5136*, provides a survival advantage under hypoxia.

We hypothesized that if *Rv0080* mutations under positive selection in clinical strains provide a survival advantage under hypoxia then they should fail to complement or have reduced complementation when expressed on an isogenic Δ*MSMEG_5136 M. smegmatis* background compared to ancestral *Rv0080*. When expressing the operonic *Rv0079*-*Rv0080* from the native promoter, i.e. 250 bp upstream of *Rv0079*, the ancestral version of *Rv0080* complemented the survival phenotype of Δ*MSMEG_5136*, reaching the lower limit of detection by day 14 (Fig. 4d).

Consistent with our hypothesis regarding the adaptive role of *Rv0080* mutations, we found that key variants selected from the Vietnamese dataset, when expressed in Δ*MSMEG_5136*, increased hypoxia survival relative to the ancestral allele. Specifically, the most frequent (L121P; 14 isolates), a homoplasy resulting in a non-similar amino acid substitution (V49G), and a frameshift, all either failed to or only partially complemented Δ*MSMEG_5136* with between 10^3^ and 10^4^ viable cells remaining at day 14 (Fig. 4d). Each of these strains displayed a survival advantage ranging between roughly 1 and 2 logs relative to the ancestral allele at days 7 and 10 (Fig. 4d). We detected only a minor degree of variation in the survival of each strain across a replicate of this experiment (Supplementary Fig. 19), and consistently observed that each *Rv0080* variant resulted in enhanced hypoxia survival relative to the ancestral allele.

### Repeated pseudogenisation of *Rv0080*’s closest homologue, *Rv3129*, in *M. canettii*

When inspecting the alignment of *Rv0080*’s *Mtb* and *M. smegmatis* homologues, an interesting observation was that its closest, *Rv3129*, shared a high degree of identity with its *M. smegmatis* orthologue, *fsq* (61.6%; Supplementary Fig. 18a, b), yet only matched a fraction (99 of 147 residues; 67.3%) of its sequence. When viewing alternate reading frames of the upstream sequence, we found an out-of-frame match for the remainder of *fsq*. By inserting a single nucleotide 13 bp upstream of the annotated start codon of *Rv3129*, we retrieved a full-length match to *fsq* with 59.3% identity (Supplementary Fig. 18c). We were interested to pursue this observation, given *fsq* and its orthologue, *Rv3129*, are both dormancy regulon genes.

To investigate the evolutionary timing of this pseudogenisation event, we searched the reconstructed *Rv3129* sequence within the genomes of a collection of five *M. canettii* strains, which display varying genetic distances to *Mtb*^39^. This revealed the same frameshift-inducing deletion in *Rv3129* in the two strains of *M. canettii* which are genetically closest to *Mtb* (strains A and D; Supplementary Figs. 20, 21). We concluded that the pseudogenisation of *Rv3129* took place relatively recently during the stepwise emergence of *Mtb* from *M. canettii*. Intriguingly, amongst the three *M. canettii* strains that are more distantly related to *Mtb*, we documented independent *Rv3129* pseudogenisation events in two of them (strains K and J; Supplementary Figs. 20, 21). We interpreted this finding as evidence of ongoing selection for a loss of *Rv3129* function across the evolutionary history of *M. canettii*: a finding warranting further investigation given this gene’s close genetic and presumed functional relationship to *Rv0080*.

### Positive selection within the *zur* gene and its promoter region amongst lineage 1 isolates

We found one other gene to display selection of a comparable magnitude to *Rv0080* and *Rv0042c*: the zinc uptake regulator gene (*zur*). *zur* encodes a transcription factor involved in zinc homeostasis, and acts to repress genes involved in zinc uptake via binding a motif overlapping their −10 and/or −35 promoter elements in a zinc-dependent manner^40,41^. In contrast to other bacterial species, in *Mtb*, *zur* forms an operon with the *smtB* gene, which is a zinc-binding transcriptional factor implicated in regulating the expression of genes involved in zinc efflux, such as *zitA* in *M. smegmatis*^42^. *smtB* also regulates the expression of the *smtB*/*zur* operon itself, by binding a 26 bp sequence motif overlapping its promoter element when in a zinc-free state.

**Fig. 5:**
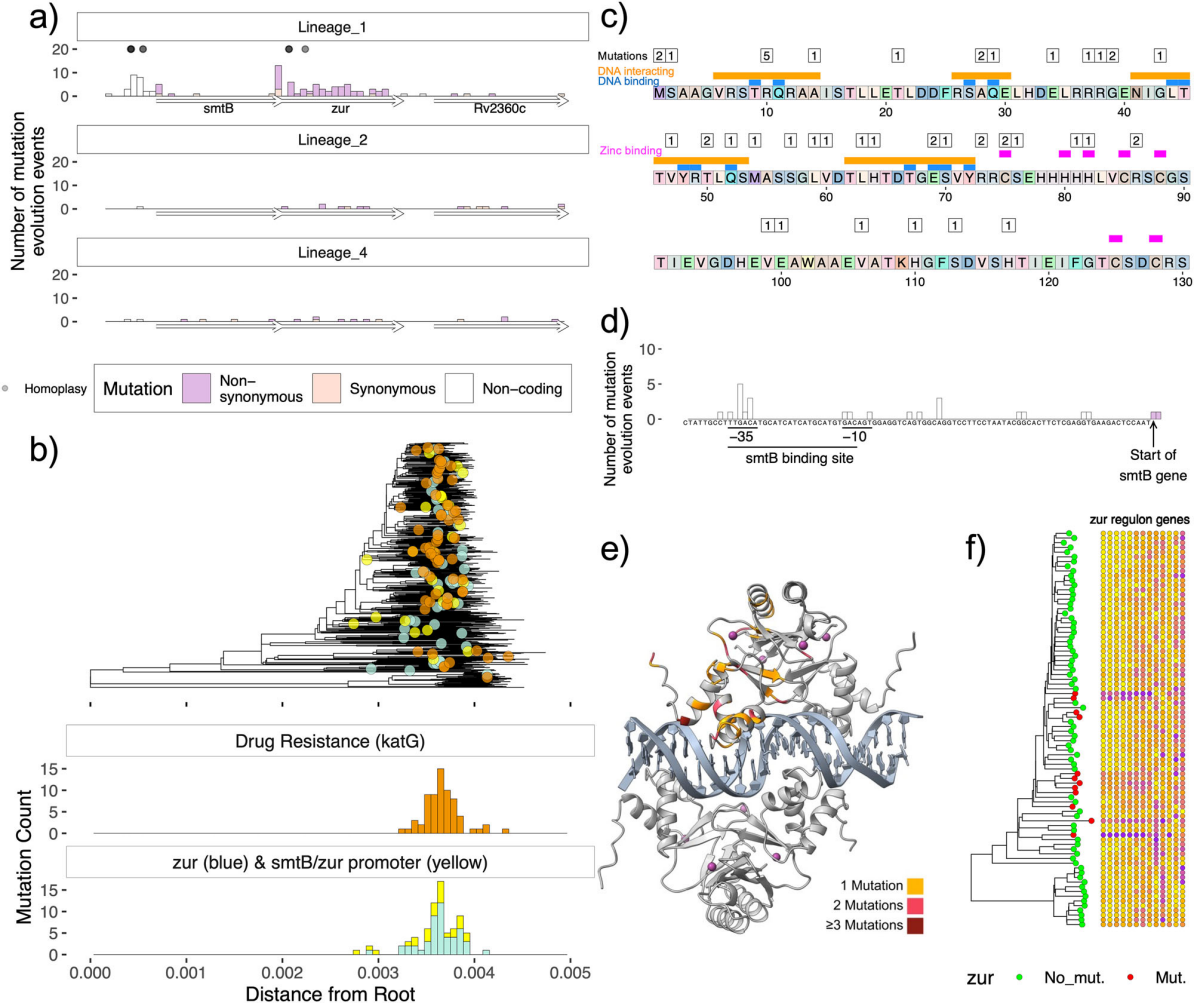
Selection for *zur* mutations and their regulatory consequences. **a** Histogram showing the count of non-synonymous, synonymous and non-coding mutations across *zur* and surrounding genes for lineages 1, 2 and 4. Grey points above each histogram show the locations of lineage-specific homoplasies. **b** (Upper) phylogeny of lineage 1 isolates with non-synonymous mutations in *katG* (orange), *zur* (light blue) and the *smtB*/*zur* promoter region (yellow) marked as points on the branches on which they were inferred to occur. (Lower) histograms showing the distribution of the distance of these *katG*, *zur* and *smtB*/*zur* promoter mutations from the root of the tree. **c** The *zur* amino acid sequence in *Mtb* (H37Rv) with inferred DNA-interacting, DNA-binding and zinc-binding residues marked in orange, blue and magenta lines, respectively. White boxes above each residue indicate the number of amino acid alterations that evolved across the Vietnamese lineage 1 isolates within that residue. **d** Histogram showing the distribution of mutations occurring across all lineage 1 isolates within the *smtB*/*zur* promoter region. Location of the *smtB* binding site, and −35 and −10 motifs are marked with black bars. **e** AlphaFold3 model of *Mtb zur* (light grey) and DNA (dark grey) analysed by DeepPBS, and mutations occurring amongst L1 isolates annotated in a colour scale (orange = 1 mutation; red = 2 mutations; dark red = 3 or more mutations) based on frequency in a representative *zur* protomer model. Zinc ions are shown as purple spheres. **f** Phylogeny of the 84 Vietnamese lineage 1 isolates for which RNA-seq data were available. Tip point colour designates whether the isolate possesses a *zur* mutation (either amino acid or promoter region), and heat-scale grid indicates the normalised expression level of 13 *zur* regulon genes: *eccA3*, *eccC3*, *PPE4*, *esxH*, *espG3*, *eccD3*, *mycP3*, *eccE3*, *PE13*, *Rv1870c*, *Rv2990c*, *esxS* and *Rv3612c*. See Supplementary Fig. 25 for numeric values. Purple indicates high expression, and yellow indicates low expression.

The *zur* gene accumulates a total of 48 NS mutations in lineage 1 isolates, and 2 S mutations (Fig. 5a; dN/dS ratio = 7.82), a figure markedly higher than observed for lineages 2 (6 NS and 1 S) and 4 (5 NS and 2 S) (rates not measured due to <10 mutations per gene). Lineage 1 isolates also accumulated substantial counts of homoplasies (6 in total; permutation test *p* = 1 × 10^−3^), compared to zero in lineages 2 and 4. As a result of these mutation events, a total of 50/643 (7.78%) of lineage 1 isolates were found to carry a non-synonymous mutation in the *zur* gene. Extending this inference to the Pan-Asia *Mtb* dataset also revealed *zur* to be a common target of selection amongst lineage 1 genomes (38 NS, 3 S; dN/dS = 4.13; Supplementary Fig. 22; Supplementary Table 2), including strains from the L1.2.1 sublineage (11 NS, 2 S; dN/dS = 1.79).

We also documented a strong enrichment of mutation events within the promoter of the *smtB*/*zur* operon amongst lineage 1 isolates (Fig. 5a, d; Supplementary Table 3). In the 182 bp upstream of this operon, a total of 25 mutations occurred, including 11 at the same nucleotide position. In total, 54 of 643 Vietnamese lineage 1 isolates (8.40%) carried a *smtB*/*zur* promoter region mutation, meaning an overall total of 103/643 (16.0%) of L1 isolates carried at least one *zur* non-synonymous mutation, or *smtB*/*zur* promoter region mutation. We found these *smtB*/*zur* promoter mutations to fall disproportionately within the −10 and −35 motifs (Fig. 5d), however, due to the overlap of these regions with the *smtB* binding site, we could not infer whether mutations inhibit binding of RNA polymerase or *smtB* (or both).

We found both *zur* amino acid mutations and promoter mutations to be temporally recent, as indicated via comparisons of mutational ordering and phylogenetic depths to known drug resistance mutations (Fig. 5b; MWU tests *zur* A.A. vs *katG*, *p* = 0.5; *zur* A.A. vs *gid*, *p* = 0.282; *zur* promoter vs *katG*, *p* = 0.804; *zur* promoter vs *gid*, *p* = 0.745). Despite the recent evolution of *zur* amino acid or promoter mutations, we did not find them over-represented amongst isolates with phenotypic resistance to either INH (chi squared test A.A., *p* = 1; promoter, *p* = 0.695) or STR (chi squared test A.A. *p* = 1; promoter, *p* = 0.379), which likely excludes a role in drug resistance. The similarities in phylogenetic depths, lack of drug resistance associations and largely independent nature of *zur* amino acid and *smtB*/*zur* promoter mutations (with only 1 isolate in our collection possessing both) led us to hypothesis that these two types of mutation may result in a similar effect on phenotype.

By constructing an alignment of mycobacterial homologues, we found *zur* mutations to hit conserved residues more often than expected by chance (70.2% vs 60.0%; Supplementary Fig. 23), but did not yield a significant *p*-value for this comparison (*p* = 0.545). Similarly, the rate of non-similar substitutions in the *zur* gene (46.8%) yielded a marginally non-significant *p*-value when comparing to genome-wide patterns (*p* = 0.054). Although indels impacted the *zur* coding sequence, they were less numerous than those found in *Rv0080*, occurring in 4 lineage 1 isolates, and resulting in frameshifts and premature stop codons on 3 occasions.

To assess the distribution of mutations within *zur* amongst Vietnamese *Mtb* isolates, we constructed a predictive model of the *zur*-DNA complex using AF3 and analysed it using DeepPBS^43^ (Fig. 5e). To consider the hypothesis that the observed mutations decrease the affinity of *zur* for the DNA strand, we identified residues predicted to bind the strand or to interact with it (blue and orange lines, Fig. 5c). Although a number of mutations occur within these regions, we did not detect a significant enrichment of mutations in either DNA-binding or DNA-interacting residues (Mann–Whitney U Tests, *p* = 0.378 and *p* = 0.19 respectively). Despite not observing a significant level of mutation enrichment, we still identified mutations likely to influence zinc binding, such as R10 (five mutations). As arginine is one of the most favoured residues for DNA binding, its substitution to histidine (four of the five mutations) results in the replacement of a positively charged amino acid with one that has a pH sensitive side chain, and may alter the role of this residue within *zur*. Other residues of interest were D33 and R36, which have been shown in the *E. coli* orthologue to be crucial for asymmetric salt-bridge formation for cooperative binding between dimers to DNA^44^. Although these residues were not observed to acquire mutations in the Vietnamese dataset, mutations were seen in the adjacent residues, E34 and R37, which could impact salt bridge formation (Fig. 5c).

Following our observation that mutations may disrupt *Rv0080* function via multiple independent mechanisms (i.e. FAD-binding site, destabilising or dimerization), and the conclusions of a similar study of drug resistance target, *Rv0678*^45^, we explored other functional implications of *zur* mutations, like the disruption of zinc binding. *zur* orthologs have two conserved zinc-binding sites, with disruption of zinc binding at either reducing DNA-binding activity of the protein^44^. We inferred the mutations observed at residues 75 and 82, which contribute to the nitrogen-rich metal-binding site (Site B), would be predicted to impact sensing of the metal and potentially DNA binding (Fig. 5c). We also inferred that mutations in residues 81, 100, and 117 could also influence metal binding, despite not contributing to canonical *zur* metal-binding sites. We concluded that mutations arising in *zur* may have a diverse range of functional effects, including disrupting DNA binding, zinc binding, or other mechanisms like destabilise overall protein structure (e.g. V106, H110, S113, H117 are near a predicted dimer interface) or affect the translation or folding of *zur*.

In contrast to *Rv0080*, we did not find *zur* to display a dynamic evolutionary history across the deep branches of the MTBC phylogeny (Supplementary Fig. 24). Using the LAA-Z dataset to infer mutations on all lineage-defining branches revealed only a R64H mutation on the branch upstream of modern lineages (L2, 3 and 4), and 2 mutations (A55V and V60A) on the branch upstream of all isolates from lineage 6 (Supplementary Fig. 24). It was also notable that *zur* was amongst the strongest targets of positive selection amongst our collection of lineage 6 genomes. In total, we observed 10 non-synonymous mutations and 1 synonymous mutation across our 277 L6 isolates (including A55V and V60A mentioned previously; dN/dS = 3.26). We also documented 1 NS mutation across the five L9 genomes screened. No substantial signal of selection was observed for *zur* amongst the animal-adapted lineages (5 NS mutations and 0 S mutation; *n* = 493 isolates). As per our screen of Vietnamese *Mtb* isolates, we found the signal of selection for *zur* within the unfixed mutation data of Liu et al.^4^ was driven largely by isolates from lineage 1. While *zur* ranked as the gene with the 22nd highest mutational density overall, and 1st for lineage 1 isolates, its ranking fell to 102nd when screening L2 isolates only, and 82nd when screening L4 isolates only. We also failed to detect enrichment of mutations in DNA-binding or DNA-interacting residues in this dataset (Mann–Whitney U Tests, *p* = 0.456 and *p* = 0.2, respectively). We deduced that *zur* may represent an analogous example to *Rv0080*, where selection occurring in parallel in ancient lineages attempts to replicate the phenotypic change induced by a mutation fixed in modern lineages: R64H. We speculate the lack of strong selection in modern lineages may reflect the presence of the R64H mutation they have already evolved.

Thanks to the recent publication of RNA-sequencing data for a subset of the Vietnamese *Mtb* dataset^46^, we were able to gain insight into the functional effects of *zur* mutations. We compared the expression levels of the 32 known *zur* regulon genes^41^ between the 12 available lineage 1 isolates with *zur* amino acid or promoter mutations against the 72 without. Given the relatively high variability between replicates of the same strain documented for this dataset^46^, we limited our comparison to the subset of 13 *zur* regulon genes displaying strongly concordant expression levels between replicates (defined as scoring *p* < 0.05 in a Spearman’s correlation test). Our analysis revealed *zur* mutants to display significantly higher levels of expression of 9 of these 13 *zur* regulon genes compared to L1 isolates without mutations (all MWU *p*-values < 0.05; Supplementary Fig. 25; Fig. 5f). It was also apparent that certain *zur* amino acid/ promoter mutations led to consistently higher expression levels across genes, with the codon H81Y mutation, which sits between two inferred zinc binding residues, ranking as the mutant with the highest expression level for 9 of the 13 analysed genes (Supplementary Fig. 25). Similarly, T2641233G, the closest intergenic mutation to the start codon of *smtB*, ranked as the mutant with the highest expression for 2 genes, and the gene with the second highest expression for 7 (Supplementary Fig. 25). *zur* regulon expression also appeared to be generally higher in L2 and L4 compared to L1 strains without mutations, with the same 10 of 13 genes displaying significantly higher expression in both the L2 vs L1 (unmutated), and L4 vs L1 (unmutated) comparisons (MWU all *p* < 0.05; Supplementary Fig. 25).

We also observed some differences in expression of the *smtB*/*zur* operon between L1 strains with and without *zur* mutations. When aggregating strains with amino acid and promoter mutations, these *zur* mutants didn’t display significant differences in expression relative to unmutated L1 strains. Strains with promoter mutations alone, however, displayed a significantly lower level of *smtB* expression (MWU test, *p* = 1.82 × 10^−3^; Supplementary Fig. 26), and a marginally non-significant reduction in *zur* expression relative to unmutated L1 strains (MWU test, *p* = 0.0554; Supplementary Fig. 26). Strains with *zur* amino acid mutations appeared to display *zur* expression levels typical of unmutated L1 strains, and strains from lineages 2 and 4 (Supplementary Fig. 26). Collectively, our data indicates that a functional consequence of *zur* mutations is to increase the expression of genes within the *zur* regulon, leading to levels typical of modern lineages. Promoter region mutations evidently do so via a downregulation of the *smtB*/*zur* operon itself.

## Discussion

We have inferred the evolutionary trajectory of 2506 *Mtb* isolates from HCMC, Vietnam, and identified previously unrecognised genes likely to be implicated in the survival and fitness of the pathogen. A key observation from our screen is that selection may act on diverse forms of variation within the same target gene. In addition to coding-region SNVs, these forms of variation include coding-region indels (i.e. *Rv0080*) and promoter region mutations (i.e. *zur*), which each may result in analogous effects on phenotype. These evolutionary mechanisms mirror those impacting drug resistance-associated genes, which similarly confer resistance via the evolution of non-synonymous SNV’s, promoter region mutations, indels, or a combination of these^47^.

Despite the variety of these mechanisms, most prior selective screens in *Mtb* only screen for NS mutations^5,7^, or conduct separate screens for NS and promoter mutations, without integrating the two, or considering indels^3,4^. Screens that are cognizant of these three forms of variation simultaneously may possess additional power to identify gene hits. For instance, when screening NS SNPs, 78/643 (12.1%) of L1 isolates in Vietnam carry an alteration in the *Rv0080* amino acid sequence, whereas this figure rises to 94/643 (14.6%) when considering indels in the coding sequence. Similarly, 50/643 (7.78%) of L1 isolates in Vietnam carry NS SNPs in *zur*, 103/643 (16.0%) carry either a NS SNP or promoter mutation (or one of each), and an additional four carry indels in the coding sequence. In addition to boosting power to identify targets of selection, considering all forms of variation simultaneously may garner further insight into the consequences of the mutations observed. For instance, the presence of frameshift-inducing indels in *Rv0080* suggests a loss of function is one of the changes being selected for. In contrast, genes displaying high dN/dS ratios yet lower rates of frameshift mutations can be inferred to be the subject of selection for more subtle alterations in function. Examples of such targets include *Rv1779c* and *Rv0380c*, which display dN/dS ratios of 2.66 (43 NS and 6 S mutations) and 5.56 (16 NS and 1 S mutation) in the Vietnamese dataset, yet evolve only 2 and 0 frameshift inducing indels. Adding this dimension to future screens will hopefully provide context for the precise functional mechanisms being targeted by selection.

By screening for selection in a lineage-specific manner, we were also able to identify important functional variants that evolved on lineage-defining branches of the phylogeny. For instance, *zur* and *Rv0080*, two of the three strongest targets of selection amongst lineage 1 genomes, are amongst the <5% of genes which undergo an amino acid alteration on the branches that separate lineage 1 from modern lineages. A plausible hypothesis is that the *Rv0080* and *zur* mutations now fixed within modern lineages confer important functional changes to these proteins, and that ancient lineages are undergoing convergent evolution to emulate these changes. This phenomenon mirrors the previously documented *esxW* T2A mutation, which contributes to the enhanced virulence of Beijing lineage strains^17^. The observation that this same codon change was under selection amongst isolates from lineages 1 and 4 allowed the identification of the adaptive role of this mutation for the Beijing lineage. Systematic screens for variants displaying this pattern may help further illuminate the evolution of the MTBC and its lineages.

When considering *zur*, although it appeared on the periphery of a prior selective screen^4^, our analysis reveals this signal of selection to be overwhelmingly driven by isolates from lineage 1. Using transcriptomic data, we have also been able to link the presence of *zur* mutations (both amino acid and promoter), to increased expression of genes within the *zur* regulon, with certain mutations leading to consistently higher levels of expression across genes. This data also indicates that L2 and L4 isolates, which possess the R64H mutation, display levels of *zur* regulon expression exceeding unmutated L1 strains. Our genetic data has revealed that mutations in *zur* don’t tend to hit conserved residues or result in radical substitutions to a significant degree. Similarly, unlike *Rv0080*, no lineages of the MTBC complex displayed frameshift mutations, and entirely non-functional version of *zur*. Coupled with moderate degree of upregulation observed for L2 and L4, we therefore hypothesise that a complete loss or radical reduction in *zur* function may not be as advantageous as more subtle alterations in the protein. Strong upregulation of *zur* regulon genes may be advantageous for *Mtb* during certain stages of infection, however bacteria retaining some function may be better equipped to modulate expression levels, and possibly toggle between zinc-scavenging and zinc-conserving states. Our data doesn’t offer direct insight into why upregulation of the *zur* regulon may be advantageous, however, in addition to the importance of metal homeostasis in infection^48^, we note this regulon also contains two strong targets of selection: *PPE3* (Fig. 2b) and *esxH*^49^, the latter of which is antigenic. Regardless of the precise mechanism involved, this data sheds light on a key target of selection throughout MTBC evolution and prioritises it for subsequent research.

A key finding of this analysis is that an element of the dormancy survival regulon has undergone intense positive selection throughout the evolutionary history of the MTBC. Despite the attention the *dos* regulon has received^27,28^, few of its genes have been functionally characterised. Similarly, *dos* regulon genes have also not been implicated in the initial emergence of *Mtb* from *M. canettii*^5^, nor stood out in selective screens^3,7,8^. Contrary to prior studies, our analysis suggests that reductive genomic evolution has acted on the dormancy regulon of *Mtb*, specifically to eliminate or reduce the function of *Rv0080*. Evidence of this comes from the presence of fixed nonsense or missense mutations in all but two human-adapted lineages of the MTBC, and strong ongoing selection in the remaining two (L1 and L9). While it is possible that certain *Rv0080* mutations on the lineage-defining branches of the phylogeny were fixed via genetic drift, the disproportionate enrichment of these mutations in *Rv0080*, the fact that two are frameshift-inducing indels, and the proximity of the remaining mutations to functionally important residues renders this possibility unlikely.

The rate of positive selection for *Rv0080* implies that the alteration or loss of function observed confers the bacteria a strong selective advantage, which has likely contributed to the global spread of the *Mtb* pathogen. Via experimental analysis, we’ve been able to ascribe a functional significance to some of the *Rv0080* mutations observed: demonstrating in an *M. smegmatis* model that they confer a survival advantage during hypoxic conditions. Thanks to the inclusion of a frameshift allele in our assay, we can deduce that *Rv0080* variants that arose across the deep, lineage-defining branches of the *Mtb* phylogeny, specifically those leading to lineages 5 and 10, resulted in this same hypoxia survival phenotype. Although further experimental data will be required to ascertain the function of additional lineage-defining mutations including V60G and V31G, this data provides strong grounds to conclude that selection for a loss of *Rv0080* took place across the deep evolutionary history of the MTBC. This conclusion is underscored by the very low likelihood of a gene of *Rv0080*’s size accumulating 2 or more frameshift-inducing indels across the lineage-defining branches. We stress that while the genetic and functional aspects of this data are highly suggestive, replication of these findings in an isogenic *Mtb* background is needed to confirm these inferences, and clarify the precise mechanisms involved.

In contrast to *zur*, we interpret our data to indicate a comparatively stronger degree of selection for a loss of *Rv0080* function. This inference is based on the presence of frameshift mutations fixed in two lineages of the MTBC, and the tendency of mutations in *Rv0080* to hit conserved residues and result in radical substitutions. In addition to exploring whether the V60G substitution results in enhanced hypoxia survival, a key line of future investigation will be establishing whether strains with this mutation retain some degree of *Rv0080* function. We infer from the relatively low frequency of frameshift-inducing indels amongst the modern lineages from our datasets that strains possessing V60G don’t experience strong ongoing selection for a complete ablation of the *Rv0080* amino acid sequence. This observation could lend support either to the hypothesis that V60G results in a complete loss of *Rv0080* function, or that V60G mutants retain some degree of *Rv0080* function, and that doing so is beneficial to the survival or virulence of *Mtb* (as we infer for *zur*).

Deciphering the precise function of *Rv0080* may be facilitated via a more comprehensive understanding of the dormancy phase in *Mtb*, which is still poorly understood. For instance, studies sequencing lesions representing active and latent infection in non-human-primates^50^ and transmission chains in humans^51^ inferred similar substitution rates between active and latent states, challenging the paradigm that *Mtb* remains non-replicative during dormancy. Examination of lung lesions in latently infected non-human-primates revealed high variance in bacterial burden^52^, which may support a similar conclusion. Conversely, other studies sampling from human transmission chains have inferred much lower substitution rates^53^, and proposed a slowdown of this rate as the length of the dormant phase progresses^54^, suggesting total metabolic shutdown.

In conclusion, while initially seeking to identify the genetic changes explaining the clinical differences and epidemiological characteristics of ancient and modern lineages, our study has unexpectedly shed light on an aspect of the bigger picture of MTBC evolution. We have demonstrated that a key alteration to the *dos* regulon has evolved in parallel in *Mtb* lineages across the globe. While work needs to be done to unravel the precise role of *Rv0080* in hypoxia survival, we have showcased the remarkable evolutionary history of this gene, and have laid a framework for identifying other key players in *Mtb* evolution.

## Methods

### Vietnamese *Mtb* accessions, quality control and variant calling

The Vietnamese *Mtb* sequences used for this analysis were derived from two prior genomic studies^17,55^, with the addition of a single lineage 5 accession, for use in phylogenetic inference. Fastq files for these samples (*n* = 2574) were filtered using cutadapt^56^ v3.5, using a quality cutoff of 15 and length cutoff of 20. Reads were then mapped to the H37Rv reference genome using bwa-mem2^57^ v2.2.1, followed by duplicate read removal using picard^58^ v2.25.0. Pilon^59^ v1.23 was then used to call variants using a minimum depth threshold of 10, a mapping quality of 40 and quality score of 20, producing a vcf file for each sample.

As initial quality control steps, samples were filtered on the basis of the heterozygote ratio^60^, calculated for non-masked regions of the *Mtb* genome^20^. Vcf files were then combined using bcftools^61^ v1.9, and genomic positions for which more than 10% of samples had a missing call were removed. Samples with genotype calls at fewer than 90% of sites were also filtered. Variant calls were further restricted to sites falling within the coordinates of Marin et al.^20^, and only polymorphic SNPs were retained. After applying these filters, a total of 2506 samples remained. Lineages were assigned for each sample using fast-lineage-caller^62^ v0.3.2, and a fasta file with the genotype for each sample at each polymorphic position in the *Mtb* genome was produced for subsequent phylogenetic inference.

### Ancestral sequence reconstruction and dN/dS inference

A phylogeny was constructed for all *Mtb* isolates using RAxML^63^ v8.2.12, and a GTR model of nucleotide substitution. SNPPar^22^ v1.2 was then applied individually to the lineage 1, 2 and 4 subtrees to perform ancestral sequence reconstruction. The count of non-synonymous and synonymous mutations within each gene for each lineage were obtained by applying custom scripts to the SNPPar output. These counts were translated into dN/dS ratios using the formula dN/dS = (non-synonymous mutation count/potential non-synonymous mutation count)/(synonymous mutation count/potential synonymous mutation count)^3^. The potential NS and S mutations per gene were calculated using custom scripts, which considered the abundance of each of the 64 possible codons the non-masked portion of that gene. dN/dS was calculated separately for each lineage after summing all mutations for that gene across lineages 1, 2 and 4. Counts of homoplasies (i.e. the total count of the amino acid changes that evolve multiple times independently across the phylogeny) per gene were generated both on a per-lineage level and across all lineages using the SNPPar output.

### Gene categorisation and antimicrobial resistance analysis

We obtained the functional categorisation for each gene from the Mycobrowser catalogue (release 4), available from https://mycobrowser.epfl.ch/releases. Data on gene essentiality were obtained from Comas et al.^64^. To compare the relative depth and ordering of mutations in gene hits against those found in known antibiotic resistance genes, we applied custom scripts to the SNPPar output. We also incorporated the phenotypic drug resistance data described in Silcocks et al.^55^ to test associations between mutations in gene hits and antibiotic resistance phenotypes.

### Inter-species amino acid alignment and conservation grading

Homologues of the *Rv0080*, *zur* and *Rv0042c* proteins in other mycobacterial species were obtained from the NCBI database^65^ via blastp. These amino acid sequences were concatenated into a multi-sample fasta file and aligned using Clustal Omega^66^ v1.2.4.

The conservation of residues in the alignment was scored using the default scheme of the Clustal Omega software (https://www.ebi.ac.uk/jdispatcher/docs/faqs/bioinformatics/). To elaborate, this scheme lists 9 groups of strongly similar residues (STA, NEQK, NHQK, NDEQ, QHRK, MILV, MILF, HY and FYW) and 11 groups of weakly similar residues (CSA, ATV, SAG, STNK, STPA, SGND, SNDEQK, NDEQHK, NEQHRK, FVLIM, HFY). These amino acid alignments were visualised, and histograms showing the distribution of non-synonymous mutations occurring within the lineage 1 clade were plotted above.

The cells of this histogram were colour-coded according to a pairwise amino acid conservation scheme derived from the one described above. To elaborate, a pair of residues was classified as ‘strongly conserved’ if this pair appear together in any of the 9 groups of strongly similar residues. A pair of residues was classified as ‘weakly conserved’ if they don’t appear together in any of the 9 strongly similar groups, but do appear together in any of the 11 groups of weakly similar residues. Pairs of residues that don’t appear together in either strongly similar or weakly similar groups were classed as ‘non-similar’.

### Inferring amino acid sequences for isolates with indels in the *Rv0080* coding sequence

After calling variants using Pilon, we identified isolates that carried indels in the *Rv0080* coding sequence from the resulting vcf using gene coordinates from Mycobrowser v4^23^. We then constructed de novo genome assemblies for all isolates inferred to possess indels using shovill v1.1.0 (https://github.com/tseemann/shovill). We used BLAST^67^ v2.13.0 to retrieve the *Rv0080* coding sequence in each assembly.

### Collation of *Mtb* genomes from additional studies

To measure the rate of positive selection observed in *Rv0080* across a wider sampling of isolates from lineages 1, 2 and 4, we collated all accessions from six recent studies of Asian *Mtb* populations^68–73^. Throughout the text, this dataset is referred to as the Pan-Asia *Mtb* dataset.

We downloaded and called variants using an identical approach to that described above for the Vietnamese dataset, and also used the same approaches for lineage calling, phylogenetic inference, ancestral sequence reconstruction, and dN/dS calculation. After QC filtering, we were left with *n* = 981, *n* = 686 and *n* = 247 isolates from lineages 1, 2 and 4, respectively.

To measure the rate of positive selection in *Rv0080* across lineage 6, and the main animal-adapted clade of the MTBC (comprising clades A2, A3 and A4, as defined by Brites et al.), we collated all accessions listed in the studies of Coscolla et al.^74^ and Brites et al.^75^. We followed an identical approach to that described above for variant calling, phylogenetic inference and dN/dS calculation. After QC filtering, we were left with *n* = 277 and *n* = 493 isolates from lineage 6 and the animal-adapted clade, respectively.

### Comparison of *Rv0080* amino acid sequences across lineage-defining branches of the MTBC

To explore *Rv0080* variation across lineage-defining branches of the MTBC phylogeny (i.e. all branches which are not collapsed in Fig. 4b), we obtained the following strains, representative of the deepest known split within each lineage (L7: ERR756347 and ERR181435; L5: ERR1023223, ERR2383620 and ERR502501; L10: ERR2516384 and ERR2707158; L8: SRR10828835 and SRR1173725; L9: ERR181314, ERR181315, ERR4162024, ERR4192384 and SRR31579360; L6: ERR1082113 and ERR551419; L1: SRR5067296 and SRR5067486; L2: SRR5065662 and SRR5065551; L3: ERR5979447 and ERR552264; L4 SRR5067625 and SRR5073871; and the main animal-adapted clade: ERR125621 and ERR234675).

To obtain the *Rv0080* amino acid sequences of these strains, we followed an identical approach to that described above for isolates possessing indels in *Rv0080*. This included downloading the raw sequencing data for each sample, constructing a de novo assembly using shovill (https://github.com/tseemann/shovill) and obtaining the *Rv0080* coding sequence using BLAST^67^. *Rv0080* mutations or indels were inferred to have occurred on the lineage-defining branch of a lineage if all representative strains from that lineage were found to possess that same variant.

### Inferring all amino acid-altering mutations across lineage-defining branches of the MTBC

To contextualise the number of mutations we observed in *Rv0080* across the deep evolutionary history of the MTBC, we also inferred the number of amino acid-altering variants on each lineage-defining branch of the phylogeny. Amino acid-altering mutations were defined as any mutation that alters the amino acid sequence of the protein, including all non-synonymous SNPs, and indels that fall within the coding sequence of that gene. To identify all SNPs that occurred across these branches, we produced a variant callset from the Illumina fastq files for isolates representing the single deepest split within each lineage (see above). We called variants on this dataset using an identical approach to that described above for the Vietnamese dataset.

After constructing a phylogeny from this dataset, we performed ancestral sequence reconstruction using an identical approach to that described above. We then used information output by SNPPar to produce tallies of the number of non-synonymous mutations that accumulate within each gene across all lineage-defining branches. We also produced tallies of the number of indels which occur across the lineage-defining branches of the phylogeny, by implementing custom scripts to map each indel to the branch it occurred on.

Due to relatively poor sequencing coverage for accession SRR1173725, we were unable to include both lineage 8 genomes in this inference, and therefore misclassify a small number of SNPs from the remaining lineage 8 sample (SRR10828835) as occurring on the lineage-defining branch of L8. The absence of a second L8 sample makes our inferences surrounding selection in *Rv0080* conservative, as we overestimate the number of amino acid-altering mutations that occur on the lineage-defining branch of L8.

### Identification of *Rv0080* homologues in *M. smegmatis*

We identified homologues of *Rv0080* across both the *Mtb* and *M. smegmatis* (GCA_013349145.1) genomes by searching the *Rv0080* amino acid sequence using tblastn. This search identified *MSMEG_5243* (*fsq*), *MSMEG_5136* and *MSMEG_6368* in the *M. smegmatis* genome, and *Rv3129* in the *Mtb* genome (Supplementary Fig. 18a). Pairwise identities between these five proteins were calculated using Clustal Omega (Supplementary Fig. 18a). We reconstructed the ‘intact’ version of *Rv3129* by inspecting reading frames of the upstream sequence of the annotated gene as described in the main text (Supplementary Fig. 18b, c).

### *M. smegmatis* markerless deletion mutant

The markerless deletion of *MSMEG_5136* was created identically to the *fsq* mutant in Harold et al.^34^, with the primers LH1-4 replaced with the following primers:

LH101 – TTTTACTAGTGCGTTGGTCGATGCCTGCTC,

LH102 – TCGAGGTCTGCACGTCCAGCTCCTGACCTT,

LH103 – GCTGGACGTGCAGACCTCGACGCCCTGAAG and

LH104 - AATTACTAGT CACGAGGATCACCGGCAGGT

The mutant was named *ΔMSMEG_5136*.

### Construction of plasmids expressing *MSMEG_5136* or *Rv0080*

To express *MSMEG_5136*, its open reading frame plus 250 bp of upstream sequence was cloned into pMV306HNK^76^ by being swapped in for the pMV261 fragment (position 3827–4222). To express *Rv0080*, the *Rv0079*-*Rv0080* operon plus 250 bp of upstream of *Rv0079* was cloned into pMV306HNK by being swapped in for the pMV261 fragment (position 3827–4222). All plasmids were synthesized and cloned at GenScript. All variants of *Rv0080* were constructed by GenScript using their mutagenesis protocol. Plasmids and their sequences are available upon request. For clarity, the ‘ancestral’ *Rv0080* allele refers to the *Rv0080* sequence held by the common ancestor of the *Mtb* complex, (i.e. identical to the H37Rv sequence, but without the V60G mutation). The V49G, L121P and frameshift mutations were all constructed on this ancestral background (i.e. constructed in combination with G60V). All plasmids were transformed into *M. smegmatis* strains using previously described protocols^77^.

### *M. smegmatis* hypoxia experiments

Strains of *M. smegmatis* mc2 155 were grown until turbid in 5 ml HdeB minimal media supplemented with glucose (0.2%) and Kan (50 µg/ml) at 37 °C with shaking. Turbid cultures were diluted to an OD_600_ of 1.0 in HdeB minimal media, and 3 ml of diluted culture was added to 27 ml HdeB minimal media supplemented with glucose (0.2%), and Kan (50 µg/ml) in a 120 ml serum vial, sealed with a rubber stopper and grown at 37 °C with shaking (160 rpm). Serum vials and rubber stoppers were sterilised by autoclaving prior to the addition of separately sterilised media. To confirm oxygen depletion, resazurin was added at a final concentration of 0.02% w/v and decolourization monitored, signifying hypoxia. CFUs were determined by removing 100 µl culture from the vials with a hypodermic needle and syringe, diluting and spotting for viable colonies on stated days.

### *Rv0080* structural predictions with AlphaFold

AF2^78,79^ multimer predictions for *Rv0080* were run using LocalColabFold v1.5.5^80^ (https://github.com/YoshitakaMo/LocalColabFold) with the parameters --msa-mode=mmseqs2_uniref_env, --model-type=alphafold2_multimer_v3, --num-recycle=20, --amber, and --use-gpu-relax, with other parameters kept on their default setting. All predictions were computed on a NVIDIA A100 Tensor Core GPU with CUDA v12.0 installed on a MASSIVE HPC host node. AF2 outputs with the highest confidence scores and relaxed with Amber (e.g. models assigned relaxed_rank_001) were used in downstream structural analysis. AF3 predictions for *Rv0080* were run using the official AF3 web server^81^ (https://alphafoldserver.com). All output models were analysed in UCSF ChimeraX v1.8^82^.

To account for the hypothesised impact the V60G mutation (present in the H37Rv reference genome, and all strains from lineages 2, 3, 4 and 7) has on the structure or function of *Rv0080*, the *Rv0080* sequence we input into AlphaFold was an ‘ancestral’ version which did not carry this variant. Residues within 4 Å of FAD were obtained for the *Rv0080* AF3 prediction, and labelled in Fig. 3c, e, and Supplementary Fig. 6. Residues within 4 Å of FAD in the Fsq crystal structure were labelled in Supplementary Fig. 6.

### Protein thermodynamic stability prediction

Change in Gibbs free energy of folding (ΔΔG) is calculated as the Gibbs free energy of the wild-type protein (ΔG_wt_) minus the Gibbs free energy of the mutant protein (ΔG_mut_). Therefore, mutations that increase protein stability yield a negative ΔΔG value, whereas mutations that destabilise the protein structure yield a positive ΔΔG value. An increase of ΔΔG by 1 kcal/mol for a typical protein reduces its equilibrium constant for folding by a factor of ~5 at physiological temperature. However, mutations with ΔΔG values within the range of −1 to 1 kcal/mol are considered to be selectively neutral and assumed to have a minimal impact on overall protein thermodynamic stability^83,84^.

ΔΔG of *Rv0080* mutants were predicted using a modified version of the RosettaDDGPrediction software (https://github.com/jlingford/ddg_rosetta), which runs Rosetta energy calculations called via a Python wrapper^35,36^. The ΔΔG of single mutations were predicted on a single chain of the AF2 *Rv0080* model as the template and energy scores calculated using the Rosetta cartesian_ddg REF2015 protocol, which is run with the rosetta_ddg_run module and the parameters --configfile-run cartesian2020_ref2015.yaml --configfile-settings rosettampi.yaml. Saturation mutagenesis ΔΔG prediction was similarly run with the Rosetta cartesian_ddg REF2015 protocol and providing the parameters --saturation and --reslistfile to the rosetta_ddg_run module. The Rosetta cartesian_ddg REF2015 protocol produces five independent ΔΔG predictions, which were averaged to produce the final average ΔΔG score per mutation.

To predict the ΔΔG of mutations on the stability of the protein-protein interaction in the *Rv0080* homodimer interface, we used the FlexDDG REF2015 protocol. The FlexDDG protocol is run using rosetta_ddg_run --configfile-run flexddg_ref2015.yaml --configfile-settings rosettampi.yaml and providing the AF2 homodimer model of *Rv0080* as input. This protocol produces 105 independent predictions, which were averaged to produce the final average protein-protein interaction ΔΔG score per mutation.

### *zur* promoter motif inference

The coordinates of the *smtB*/*zur* promoter region were obtained from the Mycobrowser release 4 genome annotation, and the −10 and −35 motifs of this region were inferred using BPROM software (http://www.softberry.com/cgi-bin/programs/gfindb/bprom.pl). The coordinates of the *smtB* binding domain were obtained by mapping the sequence reported by Riccardi et al.^40^ onto this promoter region.

### *zur* protein modelling

AF3 predictions for *zur*, using the H37Rv sequence of *zur* (4 copies) and the DNA (double stranded) were run using the official AF3 web server^81^. The AF3-generated model of the *zur*-DNA complex was analysed by DeepPBS^43^. This identified residues predicted to make direct contact with the DNA strand as: T9, Q11, S27, Q29, L44, T45, Y48, R49, Q52, T67, E69, S70, Y72. Residues predicted to interact with the DNA strand were V6, R7, S8, T9, R10, Q11, R12, A13, A14, R26, S27, A28, Q29, E30, N41 - S53; L44, T45, Y48, R49, Q52, T62 - Y72; T67, E69, S70, Y72. Predicted zinc binding sites were residues C85 C88 C125 C128 (Site A) and C75 H80 H82 (Site B).

### PAML analysis

To provide further insight into genes targeted by selection within the Vietnamese *Mtb* dataset, we applied the CODEML algorithm of PAML v4.1.2 software package^85^ to all genes in the *Mtb* genome. The CODEML algorithm was supplied with both the phylogeny of all 2506 Vietnamese *Mtb* isolates and a .phylip file containing the nucleotide sequence of each gene for each isolate. Mutations present in each isolate were introduced into this .phylip file using custom scripts, which excluded indel calls. We applied models 0, 1 and 2 of the CODEML algorithm, and extracted for each gene the dN/dS ratio, the likelihood score of each model, the fractions of sites experiencing purifying, neutral and positive selection, and the posterior probability of each individual site being under positive selection (dN/dS > 1).

### Unfixed mutation analysis

We downloaded the unfixed mutation dataset of Liu et al.^4^ made available in file ‘All_unfixed_mutation.txt’ from the ‘resR_Project’ GitHub repository (https://github.com/MtbEvolution/resR_Project). As reported in the original study^4^, we filtered this dataset down to mutations with an alternate allele frequency between 1 and 90%, leaving the 221,853 mutations reported in the paper. For consistency with the other *Mtb* datasets analysed herein, we then filtered out calls in repetitive regions of the genome, using the coordinates of Marin et al.^20^. Separate analyses were also performed after restricting this fileset down to isolates from lineages 1, 2 and 4, using the lineage calls described in file ‘51229_Mtb_Seq_Info.txt’ available from the ‘resR_Project’ GitHub repository, and also for all five sublineages of L1. Instead of calculating the density of unfixed mutations per gene (as per Liu et al.^4^), we calculated the density of unfixed non-synonymous mutations per gene, to reduce the noise associated with the small sample size of the lineage 1 dataset.

### *M. canettii* analysis

We downloaded the five high-quality *M. canettii* assemblies described by Supply et al.^39^. These strains were 140010059 (strain A), 140060008 (strain D), 140070017 (strain J), 140070010 (strain K) and 140070008 (strain L). We identified the coordinates of *Rv3129* in each assembly by applying BLAST, using the reconstructed version of *Rv3129* (mentioned above), and translating the retrieved coding sequence. We found the same frameshift-inducing indel present in *Mtb* to be present in strains A and D, in addition to a downstream premature stop codon present in both strains (Supplementary Figs. 20, 21).

### *zur* regulon expression analysis

We downloaded the dataset of normalised gene expression levels described by Culviner et al.^46^, and screened our Vietnamese *Mtb* callset for lineage 1 isolates with both RNA-seq data and *zur* mutations (either non-synonymous SNPs, indels or promoter region mutations). Given the moderate degree of variability in expression levels reported for replicates of the same strain^46^, we first performed a Spearman’s correlation test comparing expression for the 32 *zur* regulon genes^41^ between the pair of replicates for each sample. We compared the expression levels of the 13 genes that achieved significant *p*-values between lineage 1 isolates with and without *zur* mutations, and also completed comparisons of expression between unmutated lineage 1 isolates, and isolates from lineages 2 and 4. We inferred a phylogeny of L1 strains with available RNA-seq data using the same methods as described above, and the L5 sample as an outgroup. We compared *smtB* and *zur* expression using the same approach as described above for *zur* regulon genes (after verifying there was significant correlation between strain replicates), and contrasted expression of these two genes between samples with amino acid and promoter mutations, and between lineages.

### Statistical analysis

Unless otherwise stated, all statistical analyses were completed using R software^86^.

The distributions of dN/dS ratios within genes across different functional categories were compared using a Kruskal–Wallis omnibus test, and pairwise Mann–Whitney U tests. The distributions of dN/dS ratios per gene were also compared across essentiality categories (essential vs non-essential), again using the pairwise Mann–Whitney U test.

To test for a statistically significant enrichment in the rate of homoplasies per gene, we applied a permutation test that calculates the rate of homoplasy for a given lineage across randomly sampled blocks of the *Mtb* genome. This test samples 5 kb blocks of the *Mtb* genome (with replacement), and calculates a homoplasy rate by dividing the count of non-synonymous homoplasies within that block by the count of synonymous mutations. The rates observed across 1000 blocks are combined to form a null distribution, and a *p*-value is generated for the gene of interest via comparison of its non-synonymous homoplasy rate to this null distribution.

To compare the distributions of mutation depths in the phylogeny for a gene hit against known antibiotic resistance genes, we applied a Mann–Whitney U test. We tested for an association between non-synonymous mutations in target genes and binary drug resistance phenotype status using a chi squared test.

We tested for the enrichment of mutations in conserved (i.e. marked with a ‘*’ symbol) residues of the *Rv0080*, *Rv0042c* and *zur* amino acid alignment using a chi squared test. This test considers a 2 × 2 contingency table describing whether or not an amino acid residue of the *Rv0080* protein is conserved in the alignment of mycobacterial homologues, and whether or not it has accumulated at least one mutation across the lineage 1 isolates.

The enrichment of non-similar amino acid substitutions in *Rv0080* relative to genome-wide patterns was tested using a permutation approach similar to that described above. We compared the distribution of mutation counts in predicted FAD-binding regions of *Rv0080* via a Mann–Whitney U test, after excluding mutations that inhibited transcription (codon 1) or resulted in premature stop codons (3 in total).

To test the likelihood of observing 5 or more mutations in *Rv0080* across the lineage-defining branches of the MTBC phylogeny, we also used a permutation-based approach. This test takes all amino acid-altering mutations that occur across the lineage-defining branches of the phylogeny, and randomly redistributes them across all coding regions of the *Mtb* genome. We recorded the number of mutations that *Rv0080* (an arbitrary window of 459 bp) accumulates in each iteration, and used this to create a null distribution from which a *p*-value was generated. An analogous approach was used to contextualise the likelihood of *Rv0080* evolving 2 or more frameshift-inducing indels across the lineage-defining branches of the *Mtb* complex phylogeny, by permuting just the indels across branches.

We compared the predicted ΔΔG for mutations that occur within lineage 1 isolates from our Vietnamese collection with random non-synonymous mutations induced in the *Rv0080* coding sequence. To do this, we made a list of all possible non-synonymous mutations that can occur in *Rv0080* (excluding any that don’t have associated ΔΔG values like those resulting in premature stop codons and mutations in codon 1), and took the average ΔΔG of each from five replicates. We then randomly selected 1000 of these mutations (with replacement), and compared the distribution of ΔΔG values to those occurring amongst Vietnamese isolates using a Mann–Whitney U test.

Correlation between expression levels of *zur* regulon genes between replicates of the same sample was tested using a Spearman’s correlation test. The distribution of expression levels for *zur* regulon genes between lineage 1 strains with and without *zur* mutations was compared using a one-sided Mann–Whitney U test, and the comparison of L2 vs L1 (unmutated) and L4 vs L1 (unmutated) was completed using the same approach.

### Reporting summary

Further information on research design is available in the Nature Portfolio Reporting Summary linked to this article.

## Supplementary information

Supplementary Information

reporting summary

Transparent Peer Review file

## Data Availability

All data analysed in this manuscript were drawn from previously published studies and are publicly available. All relevant sources from which data were obtained are referenced in the text.

## Code availability

Code for performing the Rosetta DDG prediction is available on GitHub (https://github.com/jlingford/ddg_rosetta).
