# Supplementary material for: Dormancy regulon reduction was pivotal to the evolution of Mycobacterium tuberculosis
Source: Nat Commun. 2026 Apr 13;17:5484. doi: 10.1038/s41467-026-71566-x (PMC13284343; doi:10.1038/s41467-026-71566-x)
Supplement: Supplementary file 1 — Supplementary Information [file 41467_2026_71566_MOESM1_ESM.pdf]

Supplementary Information

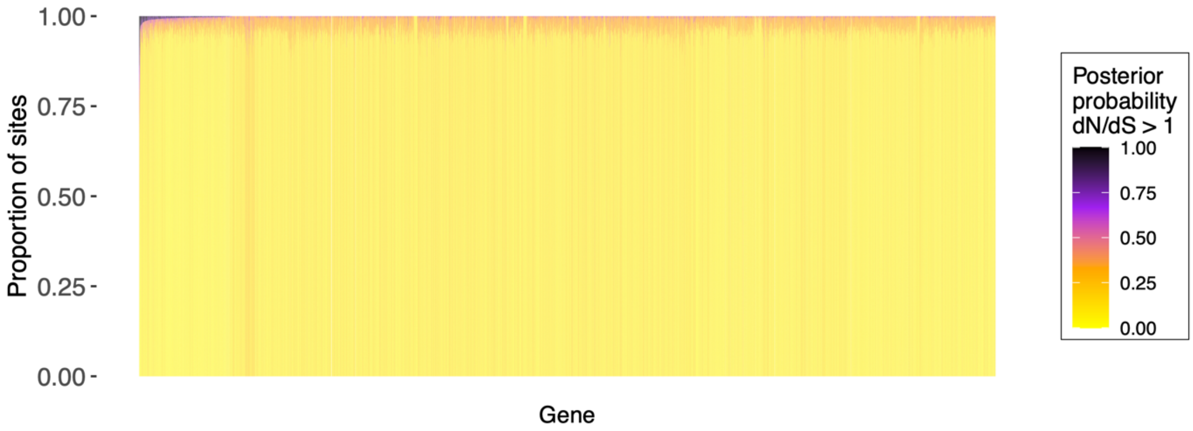

Supplementary Figure 1) Distribution of dN/dS rates across genes in the *Mtb* genome. Barplot shows the fraction of sites within each *Mtb* gene inferred by CODEML to possess posterior probabilities of positive selection over various thresholds. Genes are ordered by the fraction of sites with a posterior probability of selection of 0.9 or greater (highest to lowest).

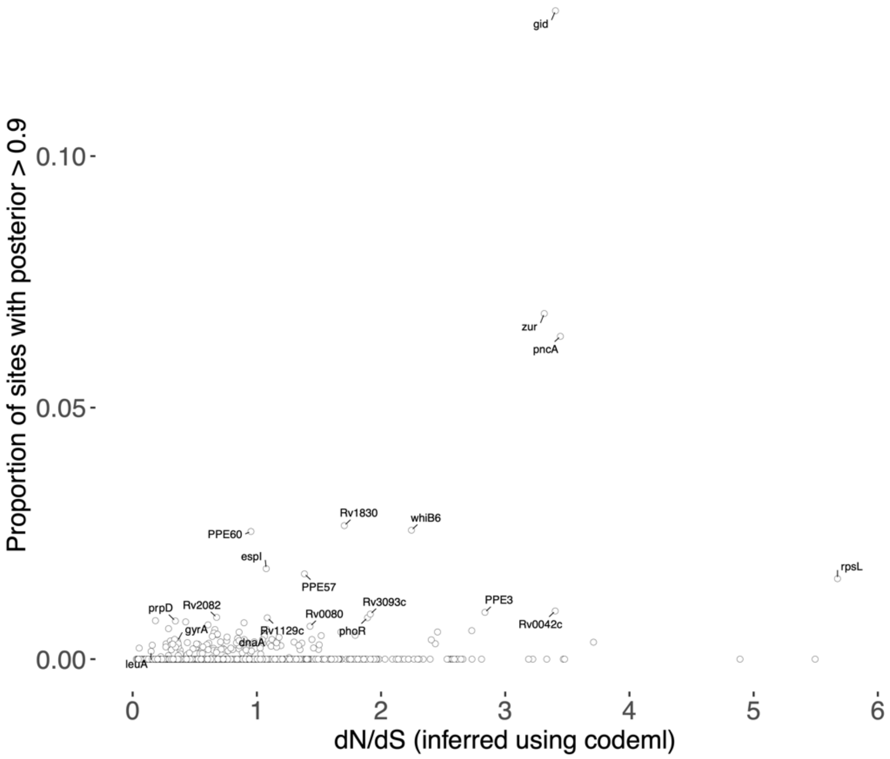

Supplementary Figure 2) Genes displaying evidence of positive selection. Scatterplot shows the dN/dS ratio for each gene (estimated using CODEML), and the proportion of sites in that gene with posterior probabilities of selection of 0.9 or greater. Only genes which evolved at least 20 mutations across the Vietnamese *Mtb* dataset are displayed.

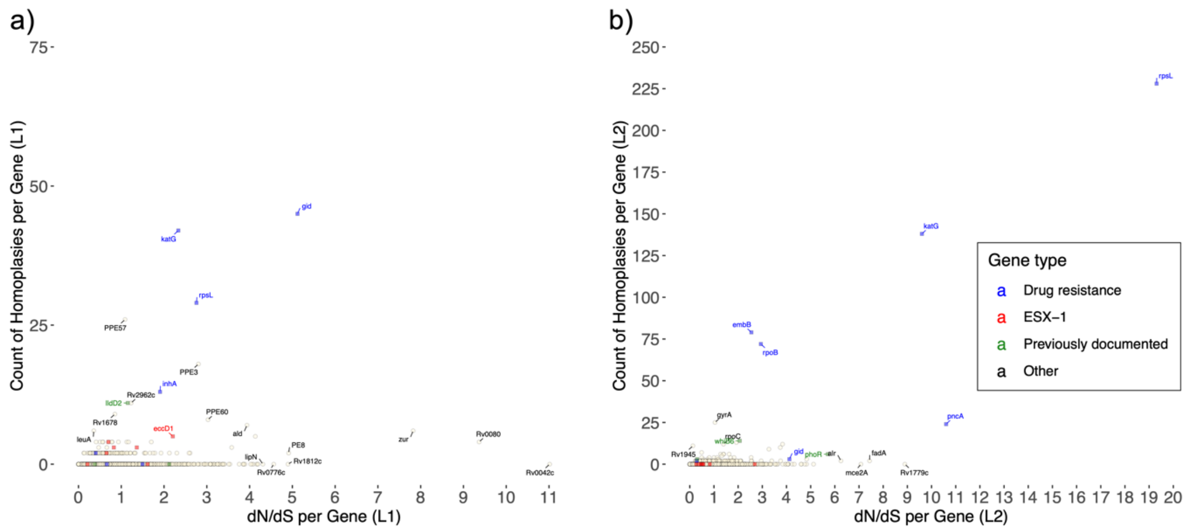

Supplementary Figure 3) Lineage-specific dN/dS rates per gene. Scatterplots show the rate of dN/dS per gene against the count of homoplastic mutations within that gene across lineages 1 (panel a) and 2 (panel b). Genes implicated in first line drug resistance (blue), ESX-1 structural elements (red) and those identified in prior screens for positive selection (green) are marked for emphasis. Note the extreme dN/dS ratios observed for *Rv0080*, *Rv0042c* and *zur* amongst L1 isolates.

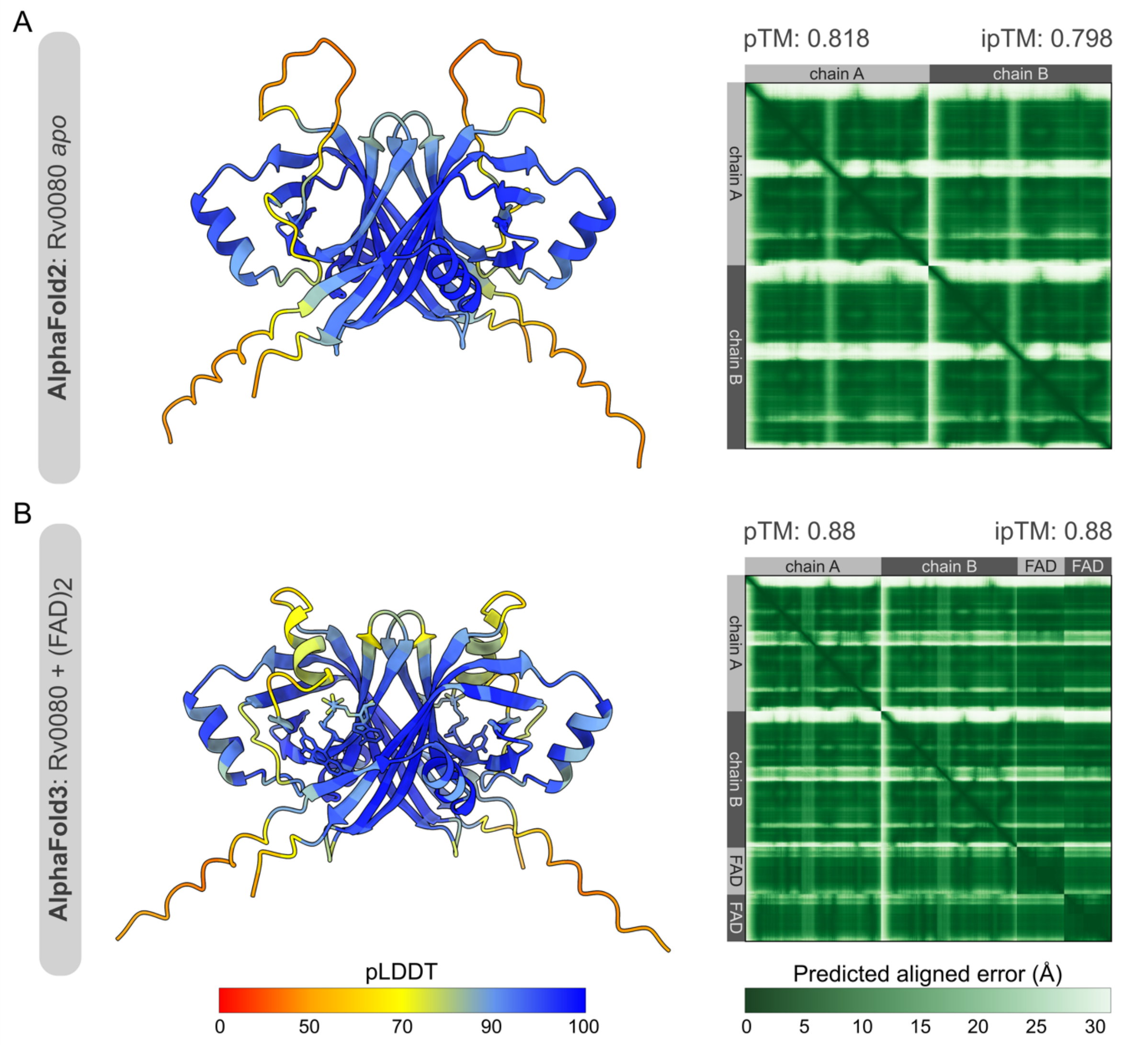

Supplementary Figure 4) AlphaFold confidence metrics. A) AlphaFold2 prediction for the *Rv0080* homodimer. B) AlphaFold3 prediction for *Rv0080* homodimer with two FAD ligands. Overall, the AlphaFold predictions are high confidence. pLDDT, predicted least distance difference test score; pTM, predicted template modelling score; ipTM, interface predicted template modelling score.

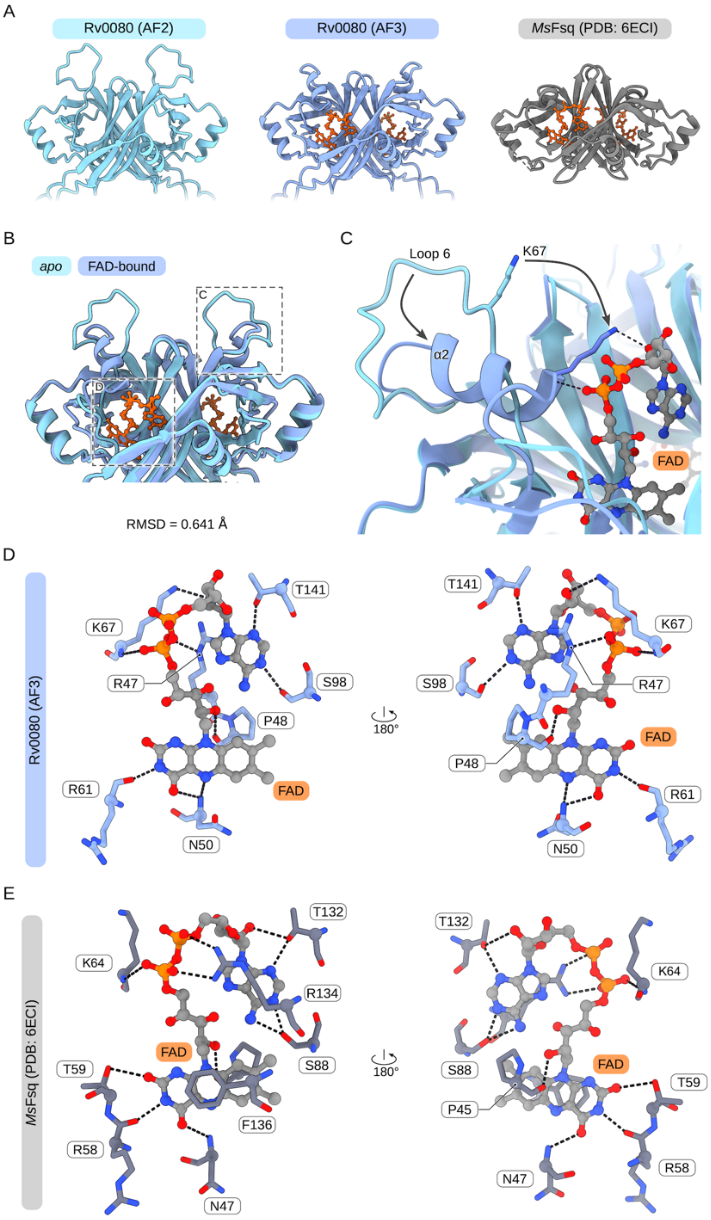

Supplementary Figure 5) Predicted structure and FAD-binding in *Rv0080*. A) Comparison between *Rv0080* AF2 and AF3 models with the X-ray crystallographic structure of *M. smegmatis* Fsq (PDB ID: 6ECI). *Rv0080* and Fsq share structural similarity. FAD ligands highlighted in orange. **B)** Superposition of AF2 and AF3 structural predictions show high agreement between both models, except for a flexible region (loop 6) in the AF2 model, which is predicted to be an alpha-helix in the AF3 model. **C)** Close-up view of the conformationally variable region. The flexible loop in the AF2 model is predicted to form an alpha-helix that binds to FAD in the AF3 model, with Lys67 (denoted K67) forming a "cap" over the FAD. This may indicate that a conformational transition in this region of the structure (loop 6/alpha-helix 2) is involved in FAD-binding. **D)** Zoom-in on the predicted FAD-binding site in *Rv0080*, with key residues predicted to form hydrogen bonds and/or cation-pi interactions with FAD shown. **E)** Zoom-in on the Fsq FAD-binding site, with key residues that hydrogen bond or pi-stack with FAD shown. Comparison between the FAD-binding site of *Rv0080* and Fsq shows that FAD-binding residues are generally conserved between both structures. Notably, *Rv0080* lacks an equivalent to Phe136 (F136) found in Fsq, which pi-stacks with the isoalloxazine ring of FAD.

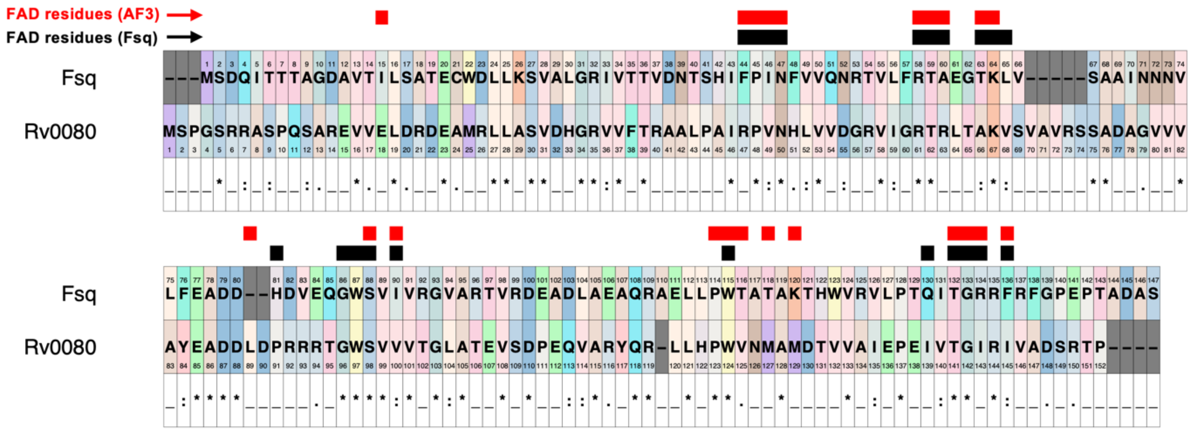

Supplementary Figure 6) Alignment of the amino acid sequences of *Rv0080* and Fsq. This alignment was produced with Clustal Omega, using default settings. Symbols below each residue indicate the degree of conservation of that residue (‘*’ designates fully conserved residues, ‘:’ strongly conserved, ‘.’ weakly conserved and ‘_’ non-conserved). Black bars above the alignment indicate Fsq residues (and their *Rv0080* equivalents) within 4 Å of FAD, based off the crystal structure described by Harold et al. (2019). Red bars above the alignment indicate *Rv0080* residues within 4 Å of FAD, as predicted using AF3.

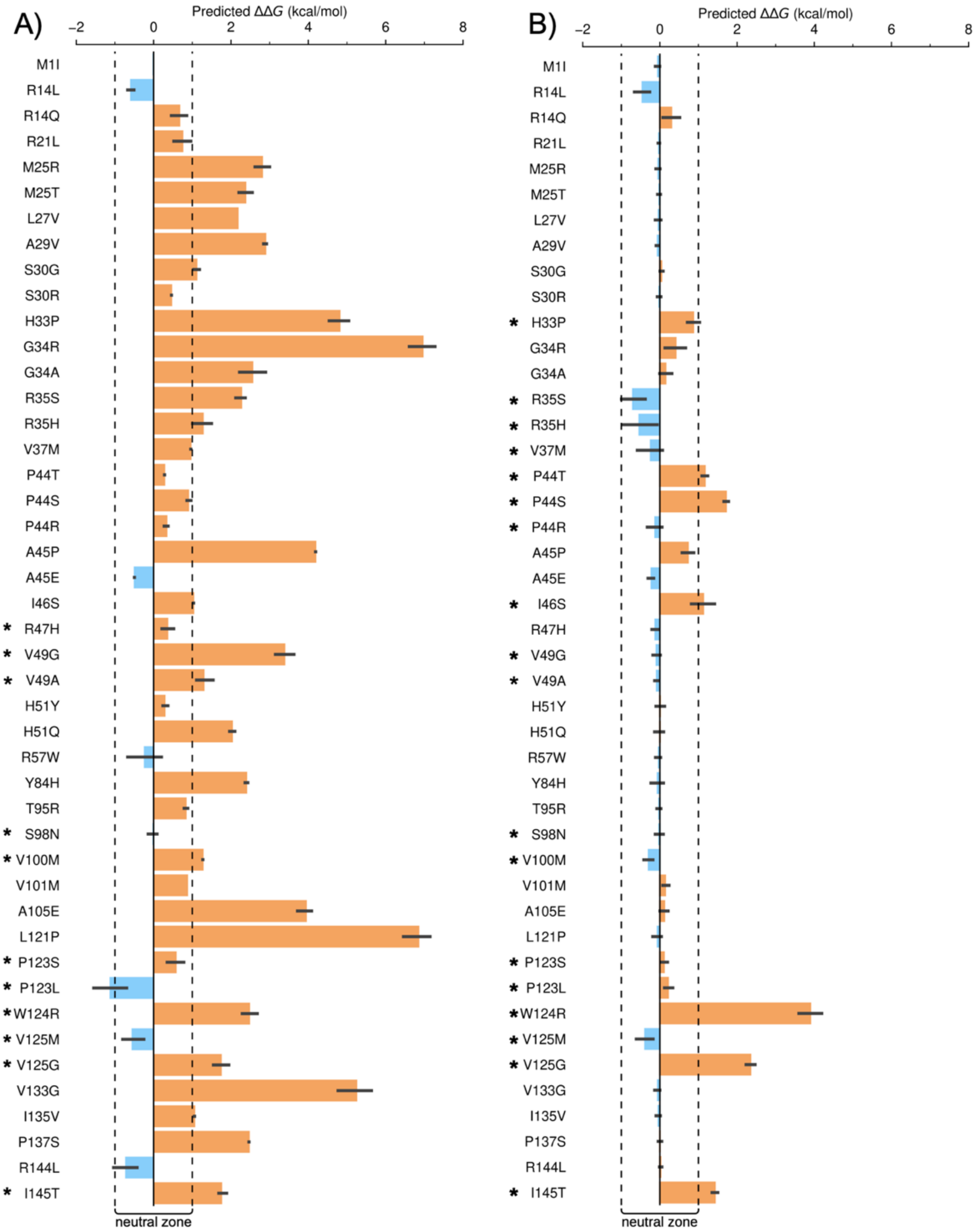

Supplementary Figure 7) Predicted ΔΔG values of *Rv0080* mutations. A) ΔΔG predicted for all *Rv0080* mutations which occur across the Vietnamese *Mtb* dataset. Mutations marked with an asterisks are within 4 Å of the FAD-binding site predicted by AlphaFold3 and might be directly involved in FAD-binding. **B)** ΔΔG predictions of mutations on the stability of the protein-protein interaction in the *Rv0080* homodimer interface made using FlexDDG. Mutations marked with an asterisks are located within the homodimer interface of the AlphaFold3 model. ΔΔG between -1 to +1 kcal/mol is considered a “neutral zone” with minimal effect on overall protein thermodynamic stability. Error bars, s.d. (n=5 in panel A; n=105 in panel B).

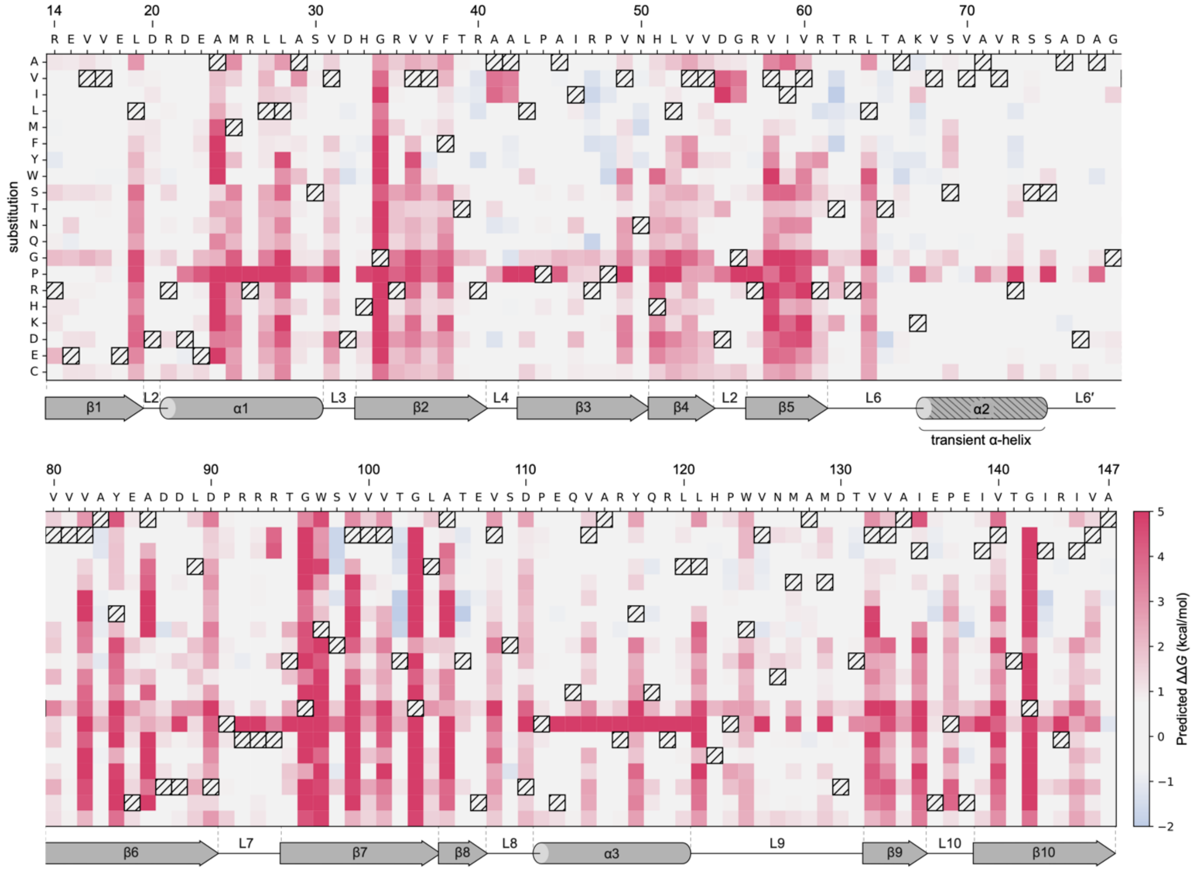

Supplementary Figure 8) ***In silico*** **saturation mutagenesis of** ***Rv0080*****.** Point mutation ΔΔG values were predicted with Rosetta using a single chain of *Rv0080* (AlphaFold2 model) as input. Overall, amino acid positions that tend to be prone to destabilising mutations are located in the secondary structure elements of alpha-helix 1 and most beta-strands, whereas mutations that tend to have a minimal effect on protein stability tend to be located in loops, beta-strand 3, and alpha-helix 2 and 3. Self-substitutions are marked with a hashed square on the heatmap. Secondary structure elements corresponding to each amino acid position are shown below the saturation mutagenesis heatmap.

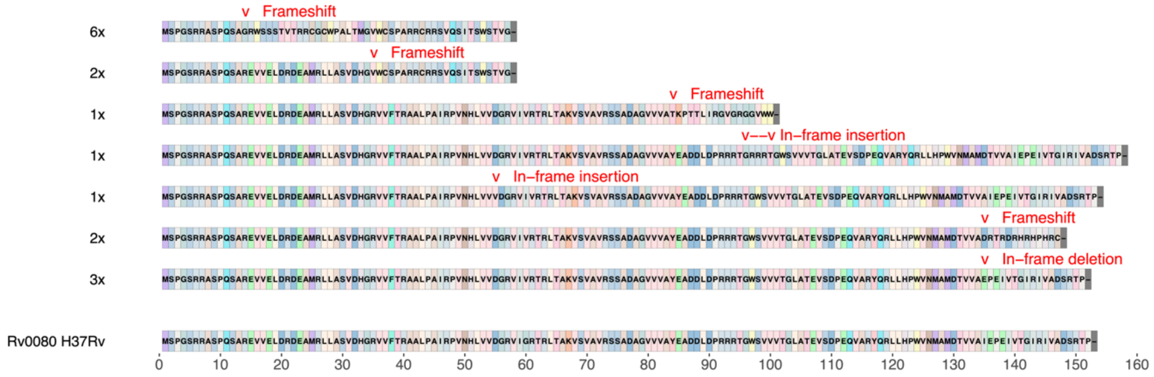

Supplementary Figure 9) Indels in the *Rv0080* coding sequence. Diagram shows all variants of the *Rv0080* amino acid sequence amongst isolates from the Vietnamese *Mtb* cohort. The number of occurrences of each variant are annotated on the left, and the location of frameshift mutations, amino acid insertions and deletions are annotated in red text. The *Rv0080* amino acid sequence from the H37Rv reference sequence is displayed below.

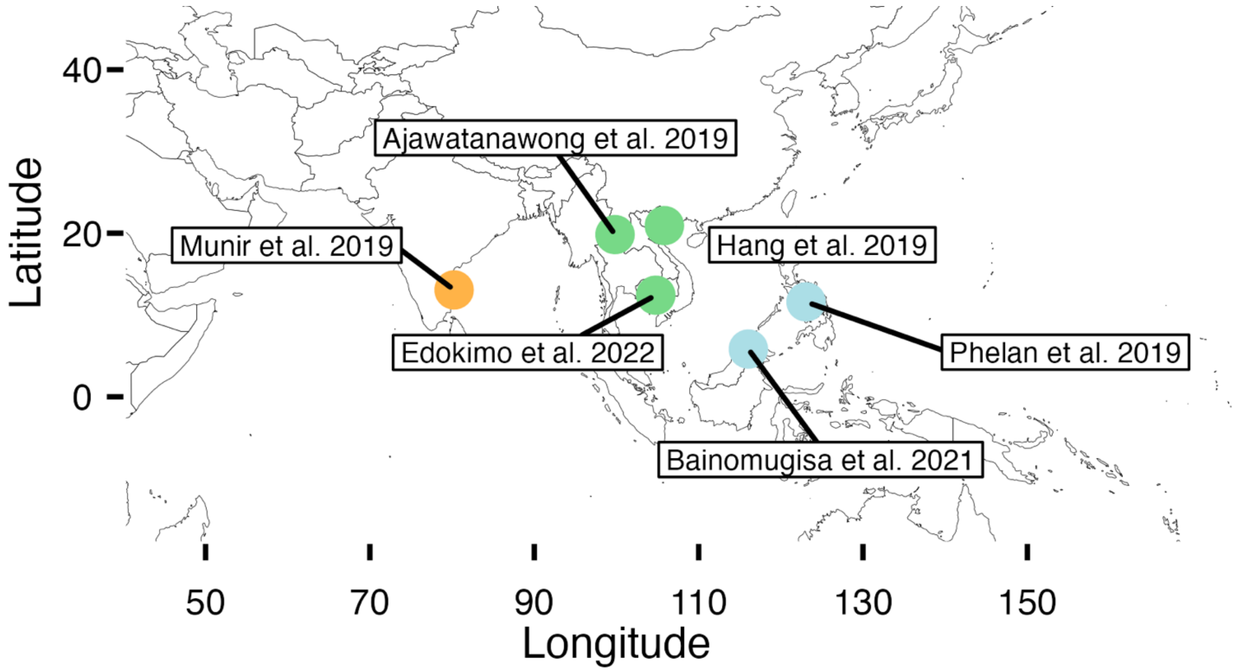

Supplementary Figure 10) Additional *Mtb* cohorts analysed. Map shows the locations of studies from which each of the six additional cohorts constituting the 'Pan-Asia *Mtb* dataset' was derived. Map was obtained using the map_data() function in R (R Core Team, 2021).

Sublineage	NS mutations	S mutations	Count w. mutations	Proportion w. mutations
L1.1.1	25	2	41/380	0.11
L1.1.2	9	0	63/86	0.73
L1.1.3	3	0	41/54	0.76
L1.2.1	2	2	5/433	0.01
L1.2.2	2	0	3/28	0.11

Supplementary Table 1) Counts of the number of non-synonymous and synonymous mutation evolution events within *Rv0080* across all five L1 sublineages from the Pan-Asia *Mtb* dataset. Also shown are the count and proportion of isolates from each sublineage possessing non-synonymous mutations in *Rv0080*.

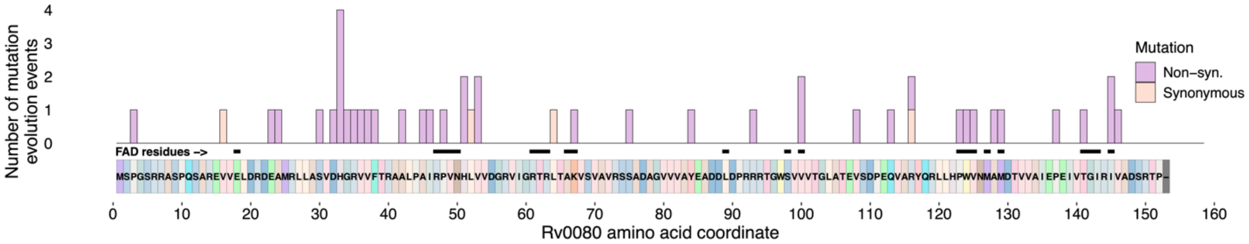

Supplementary Figure 11) *Rv0080* mutations observed amongst additional L1 genomes. (Upper) histogram showing the count of non-synonymous (purple) and synonymous (pink) mutation evolution events within the *Rv0080* coding sequence across all L1 isolates from the Pan-Asia *Mtb* dataset. (Lower) *Rv0080* amino acid sequence from the H37Rv reference genome. Black bars above the amino acid sequence indicate residues within the predicted FAD-binding region, as inferred using AF3.

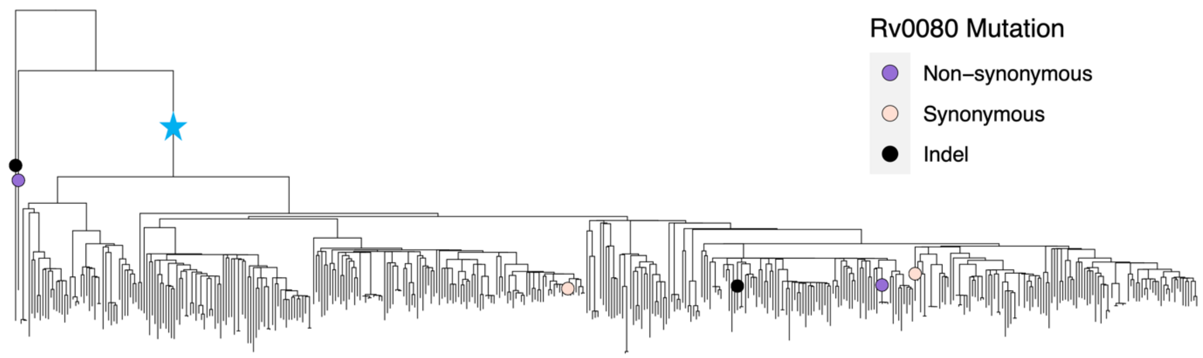

Supplementary Figure 12) Lack of selection for *Rv0080* mutations amongst L1.2.1 strains. Phylogeny shows the lineage 1.2.1 isolates from the Pan-Asia *Mtb* dataset, with *Rv0080* variants marked as points on the branches on which they were inferred to occur. Pink points correspond to synonymous mutations, purple points correspond to non-synonymous mutations, and black points correspond to indels. Note the occurrence of an indel and a non-synonymous *Rv0080* mutation on the branches descending from the first and second deepest splits within this tree. The blue star marks the branch defining the clade of isolates which do not appear to undergo selection for *Rv0080* mutations.

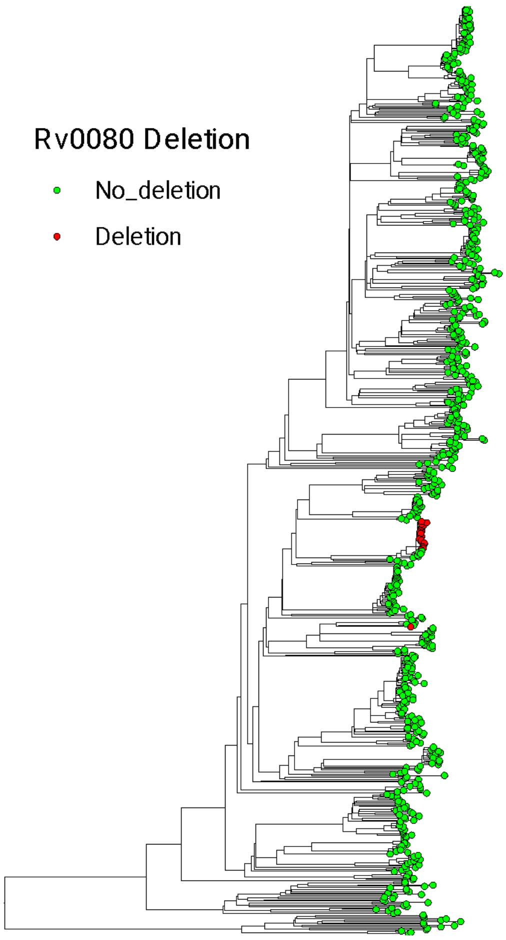

Supplementary Figure 13) *Rv0080* indels amongst L2 strains. Phylogeny depicts the lineage 2 isolates from the Pan-Asia *Mtb* dataset. Isolates inferred to possess an indel within the *Rv0080* coding sequence are marked in red, and those without are marked in green. A total of 23 L2 isolates possess indels in *Rv0080*, with 22 of these forming a single cluster.

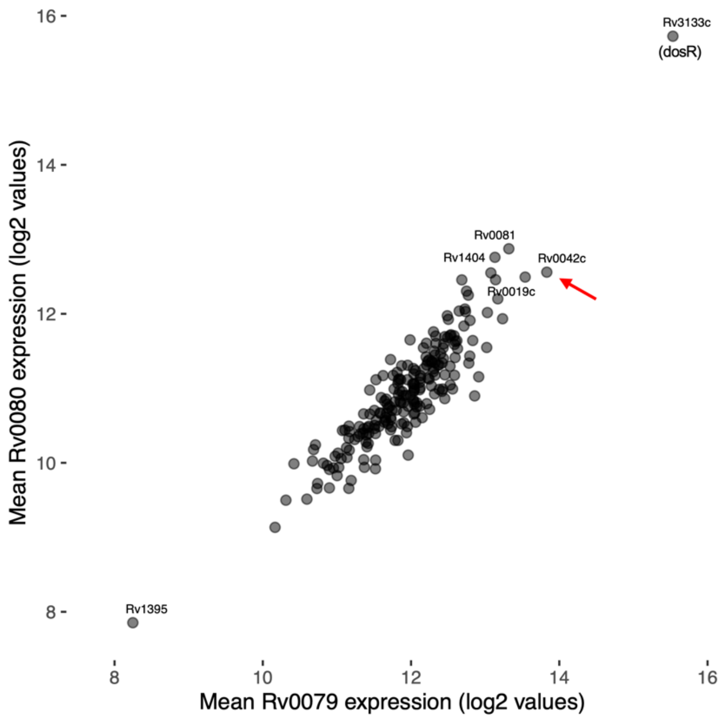

Supplementary Figure 14) Transcription factors linked to expression of the *Rv0079-Rv0080* operon. Plot shows the expression levels of *Rv0079* (x-axis) and *Rv0080* (y-axis) when each of 206 *Mtb* transcription factors (points) are overexpressed. Raw expression levels for each transcription factor were obtained from Rustad et al. (2014), and the mean of all replicates was taken. Expression levels are presented in arbitrary log2 units, as in Rustad et al. (2014). *Rv0042c* is the transcription factor resulting in the highest *Rv0079* expression level aside from *Rv3133c* (*dosR*), and the second highest *Rv0080* expression level aside from *dosR* and *Rv0081*.

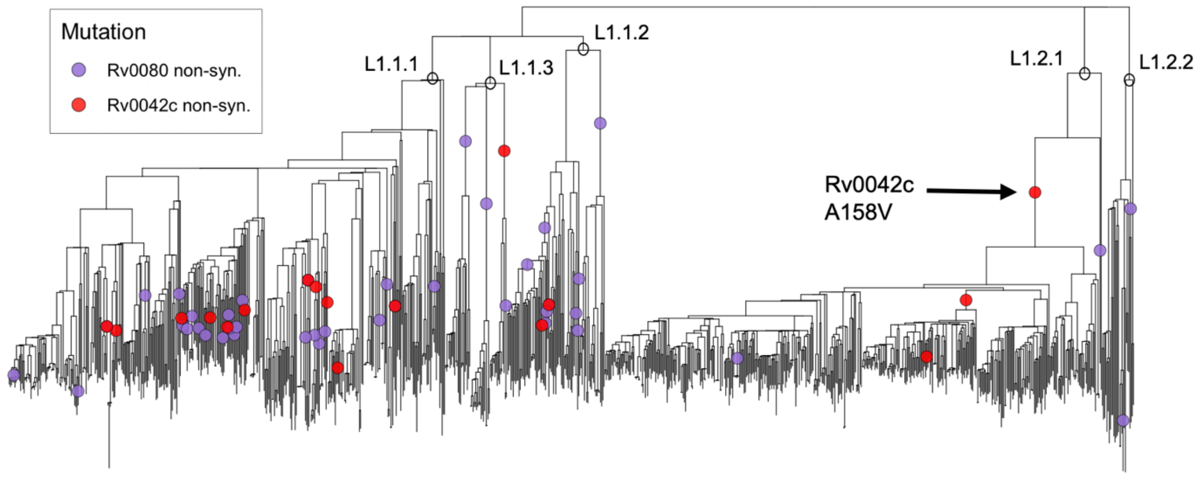

Supplementary Figure 15) Mutations in *Rv0080* and *Rv0042c*. Phylogeny of lineage 1 isolates from the Pan-Asia *Mtb* dataset, with NS mutations in *Rv0080* and *Rv0042c* marked as points on the branches on which they were inferred to occur. Purple points indicate mutations in *Rv0080* and red points indicate mutations in *Rv0042c*. The *Rv0042c* mutation arising in the L1.2.1 sublineage is labelled.

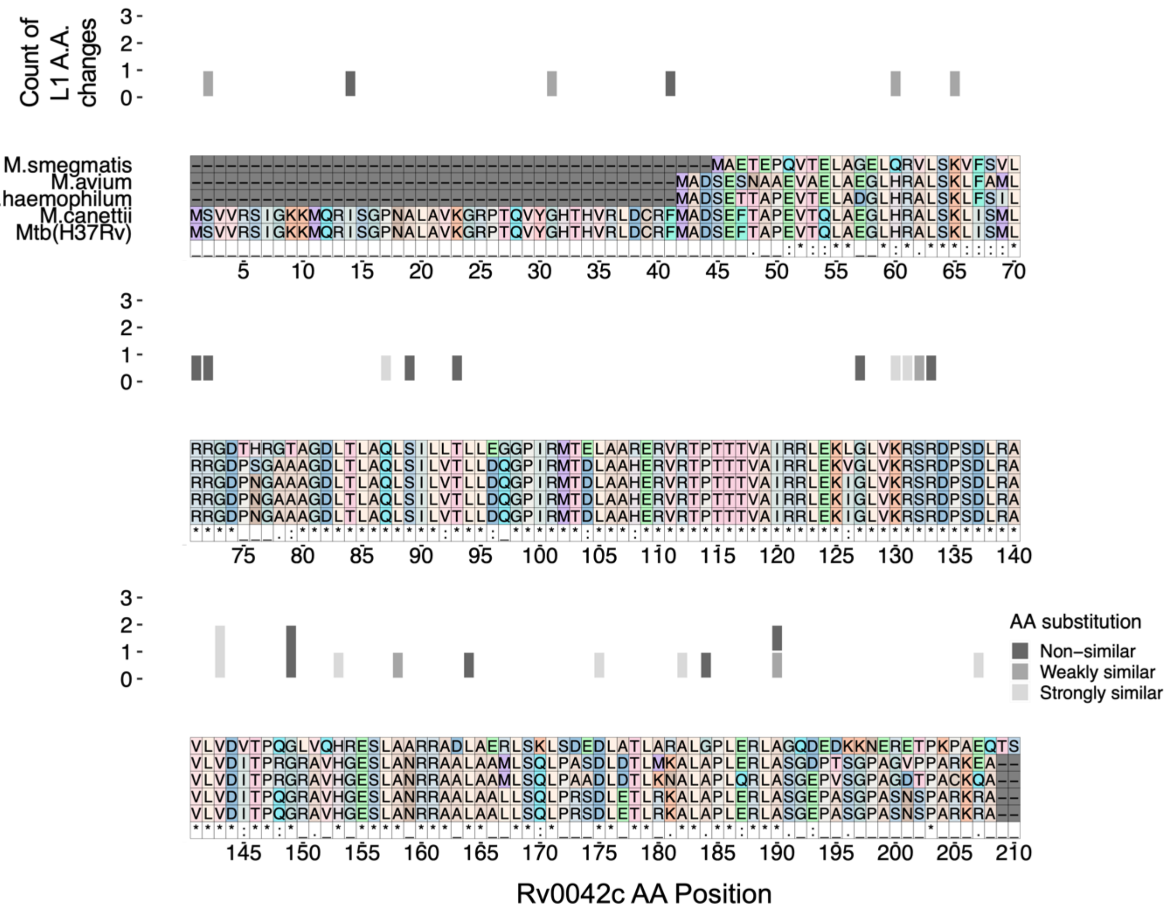

Supplementary Figure 16) Mutations within *Rv0042c*. Alignment of *Rv0042c* and its homologues in several mycobacterial species, with histogram showing the distribution of amino acid alterations across all Vietnamese lineage 1 isolates. Symbols below each residue in the alignment indicate the degree of conservation of that residue across homologues (‘*’ designates fully conserved residues, ‘:’ strongly conserved, ‘.’ weakly conserved and ‘_’ non-conserved). Colour coding of each cell of the histogram indicates the type of amino acid change for each lineage 1 mutation.

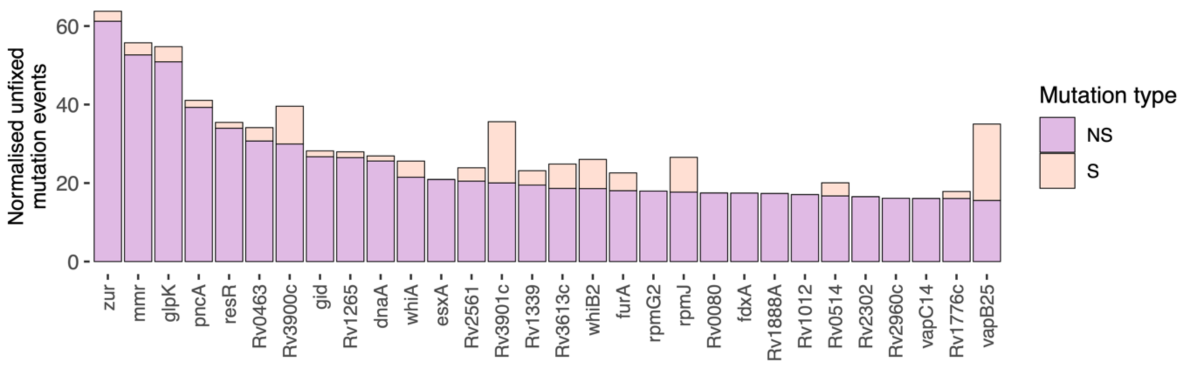

Supplementary Figure 17) Rates of unfixed mutations per gene amongst L1 strains. Barplot shows the mutational density per gene inferred from the unfixed mutation dataset of Liu et al. (2022). Mutational densities were calculated when considering mutations identified across isolates belonging to lineage 1 only, and genes are ranked based off the density of non-synonymous mutations. The genes with the top 30 highest NS mutation densities are shown.

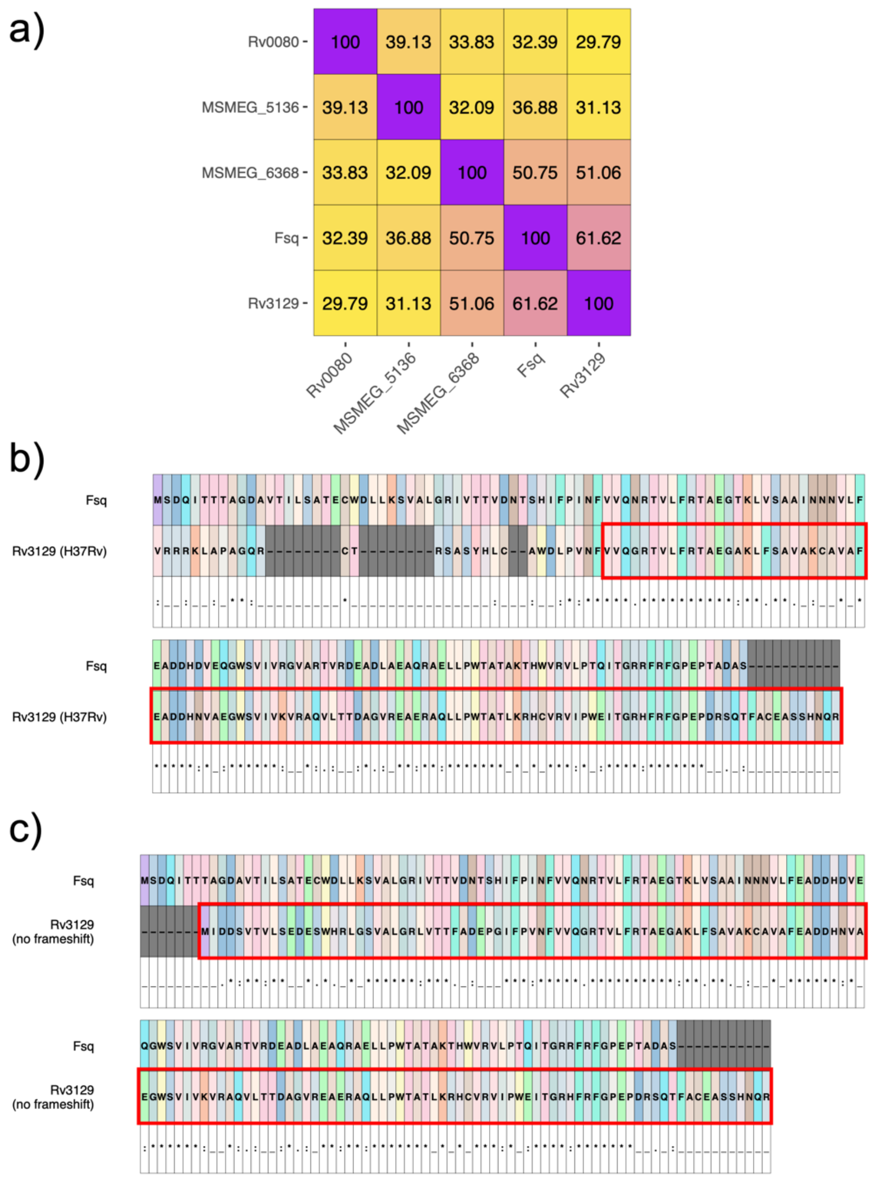

Supplementary Figure 18) *Rv0080* homologues in *Mtb* and *M. smegmatis*. a) Heatmap showing percentage amino acid identity between *Rv0080* and its four homologues identified in the *Mtb* (*Rv3129*) and *M. smegmatis* (*MSMEG_5136*, *MSMEG_6368* and *fsq*) genomes. **b)** Alignment of the amino acid sequences of *fsq* and *Rv3129* (annotated according to Mycobrowser, v4) and its upstream sequence. Symbols below each residue indicate the degree of conservation of that residue ('*' designates fully conserved residues, ':' strongly conserved, '.' weakly conserved and '_' non-conserved). **c)** Alignment of the amino acid sequence of *fsq* and *Rv3129*, after inserting a single 'T' nucleotide at position 3,494,647 of the H37Rv reference genome to restore the reading frame (see Methods). Red rectangles indicate putative reading frames of the *Rv3129* protein.

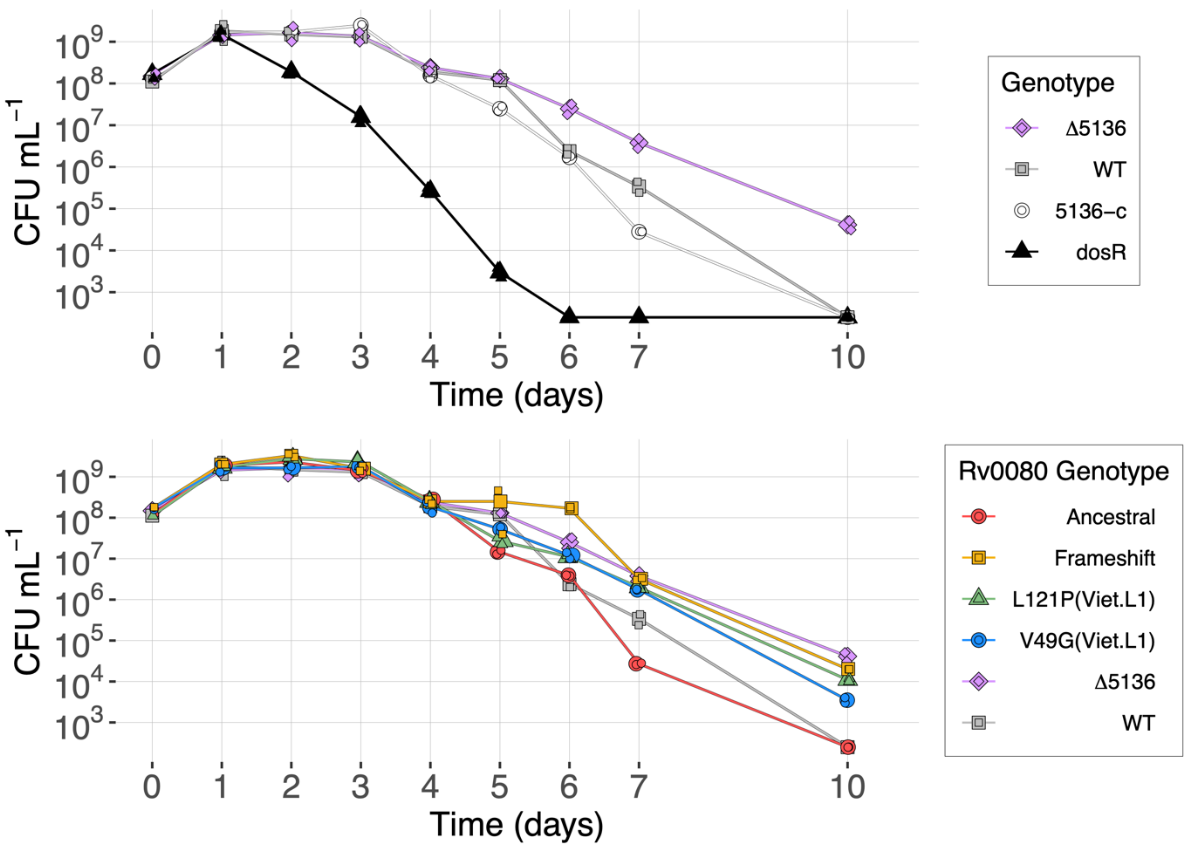

Supplementary Figure 19) Replicate of the hypoxia survival experiment described in Figure 4c and d. (Upper) Survival of Δ*dosR*, Δ*MSMEG_5136*, Δ*MSMEG_5136*-Complement and the wild type *M. smegmatis* strain (measured as colony forming units) between the timepoint zero, and the completion of the hypoxia experiment (10 days). Small points indicate individual replicates (duplicate), and large points indicate the mean. (lower) Survival of *M. smegmatis* Δ*MSMEG_5136* strains expressing various *Rv0080* alleles selected from amongst Vietnamese lineage 1 strains.

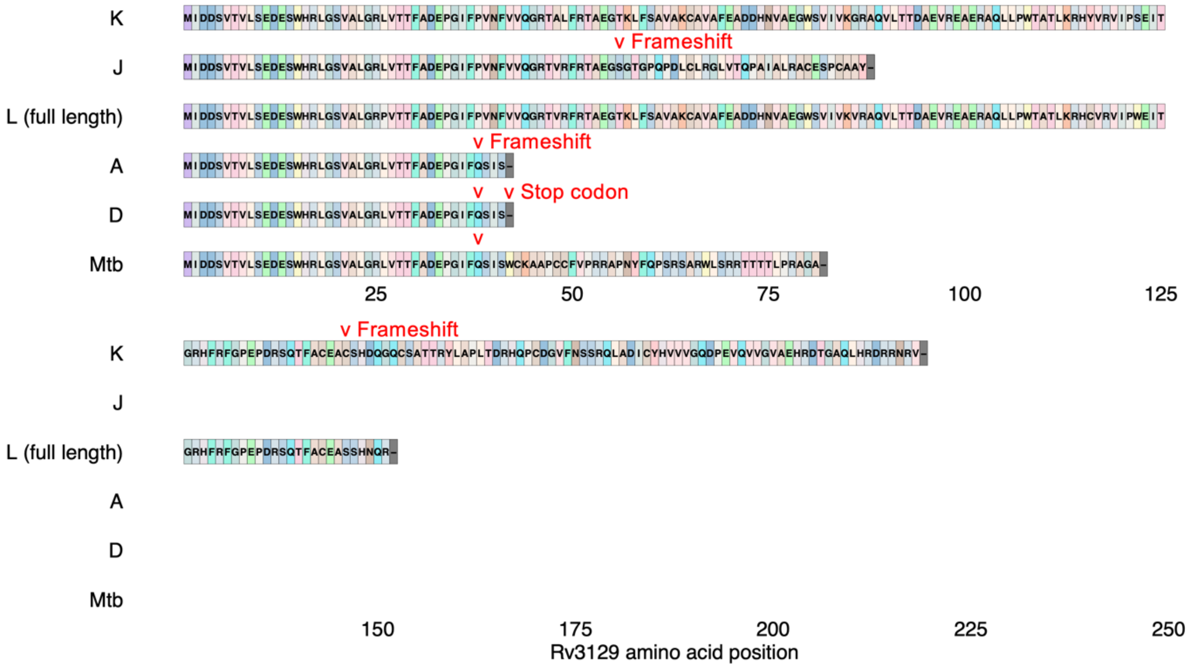

Supplementary Figure 20) Variants of the *Rv3129* amino acid sequence amongst *M. canettii* strains K, J, L, A and D compared to the amino acid sequence of *Mtb*. Strain L is the only one found to possess a full-length, intact version of the gene. The location of the frameshift mutations which occur in strains K, J, A, D and in *Mtb* are labelled in red. Note that strains A and D also possess an additional stop codon, which evolved after the frameshift mutation these strains share with *Mtb*.

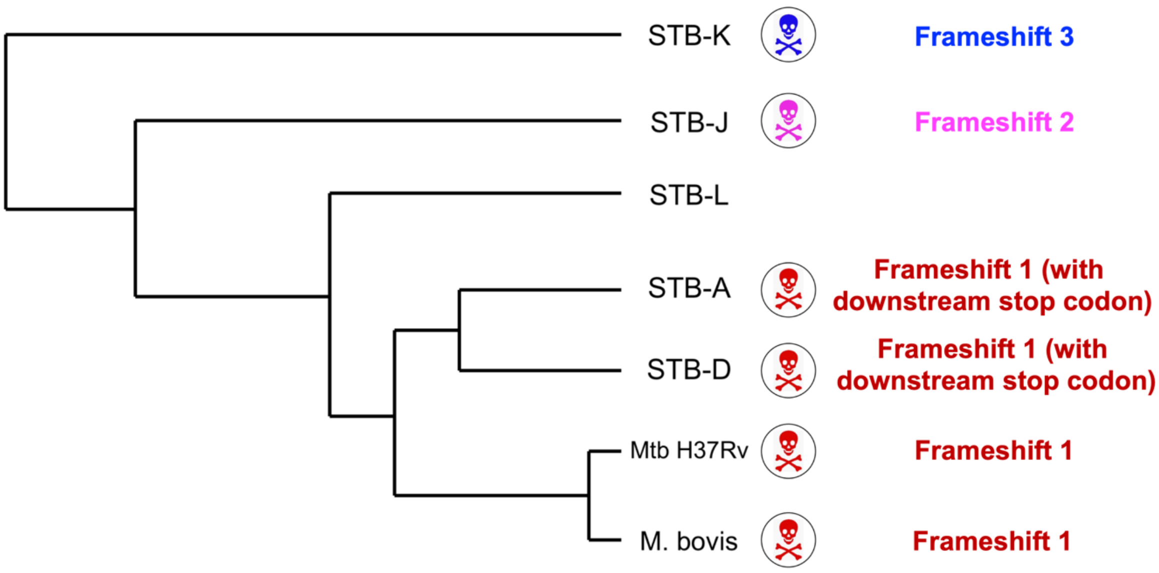

Supplementary Figure 21) *Rv3129* alleles across *M. canettii* strain diversity. The tree depicting relationships between *M. canettii* strains was reproduced from Supply et al. (2013) with approximate branch lengths. Variants of the *Rv3129* amino acid sequence are annotated alongside each strain.

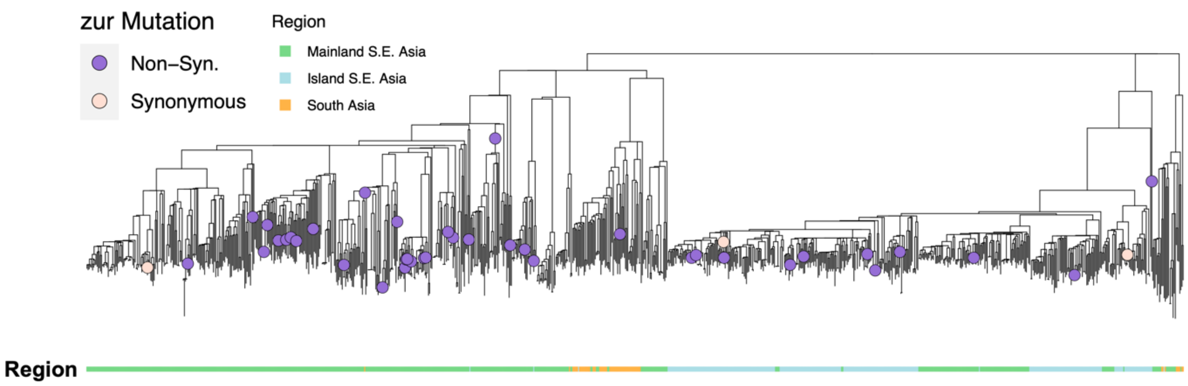

Supplementary Figure 22) *zur* evolution across a wider L1 dataset. Phylogeny shows L1 isolates from the Pan-Asia *Mtb* dataset, with *zur* variants marked as points on the branches on which they were inferred to occur. Pink points correspond to synonymous mutations and purple points correspond to non-synonymous mutations.

Sublineage	NS mutations	S mutations	Count w. mutations	Proportion w. mutations
L1.1.1	24	1	57/380	0.15
L1.1.2	1	0	1/86	0.012
L1.1.3	2	0	2/54	0.037
L1.2.1	11	2	10/433	0.023
L1.2.2	0	0	0/28	0

Supplementary Table 2) Counts of the number of non-synonymous and synonymous mutation evolution events within *zur* across all five L1 sublineages from the Pan-Asia *Mtb* dataset. Also shown are the counts and proportion of isolates from each sublineage possessing non-synonymous mutations in *zur*.

Sublineage	Promoter mutations	Count w. promoter mutations	Proportion w. promoter mutations
L1.1.1	11	12/380	0.032
L1.1.2	3	5/86	0.058
L1.1.3	0	0/54	0
L1.2.1	0	0/433	0
L1.2.2	0	0/28	0

Supplementary Table 3) Counts of the number of *smtB*/*zur* promoter region mutation evolution events across all five L1 sublineages from the Pan-Asia *Mtb* dataset. Also shown are the counts and proportion of isolates from each sublineage possessing a *smtB*/*zur* promoter region mutation.

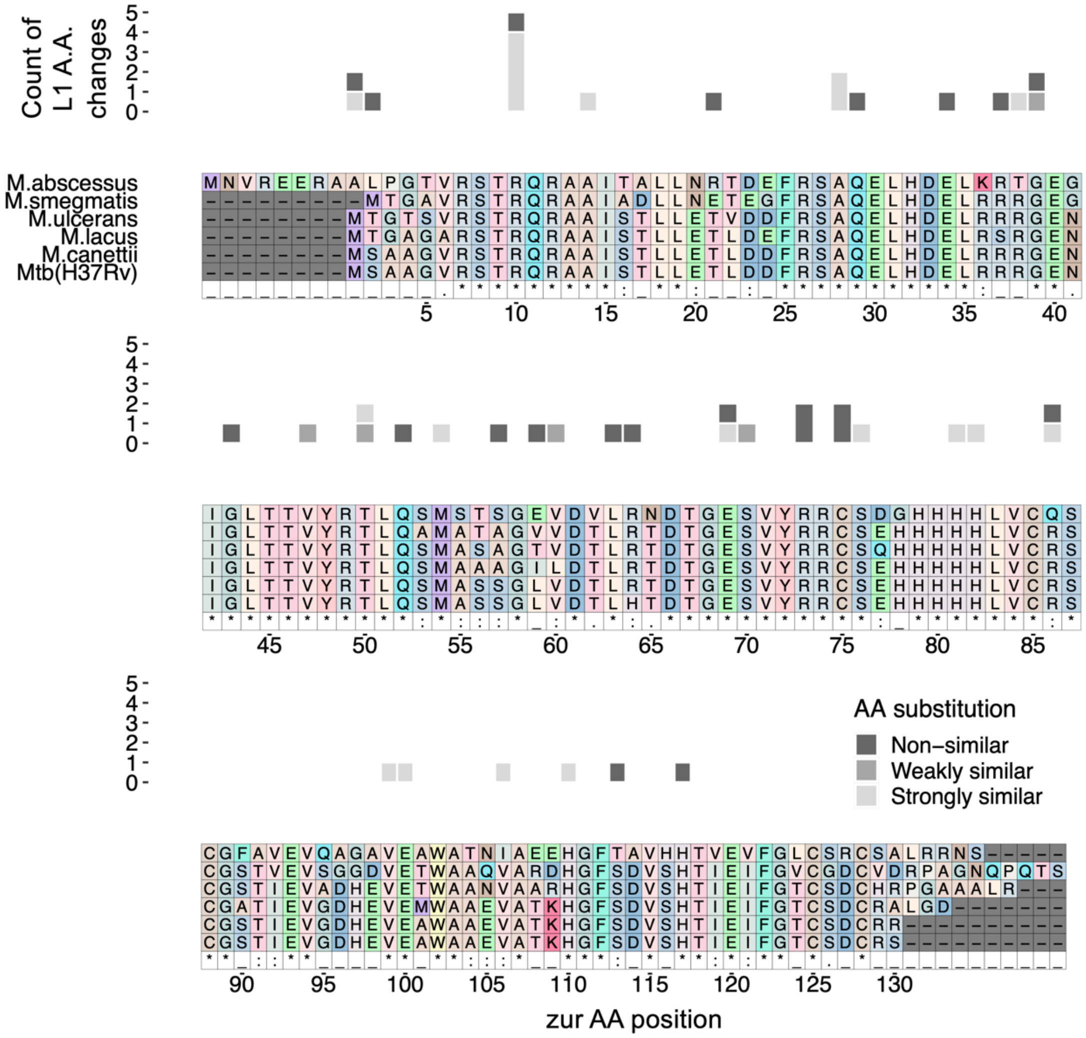

Supplementary Figure 23) Distribution of *zur* mutations. Alignment of *zur* and its homologues in several mycobacterial species, with histogram showing the distribution of amino acid alterations across all Vietnamese lineage 1 isolates. Symbols below each residue in the alignment indicate the degree of conservation of that residue across homologues ('*' designates fully conserved residues, ':' strongly conserved, '.' weakly conserved and '_' non-conserved). Colour coding of each cell of the histogram indicates the type of amino acid change for each lineage 1 mutation.

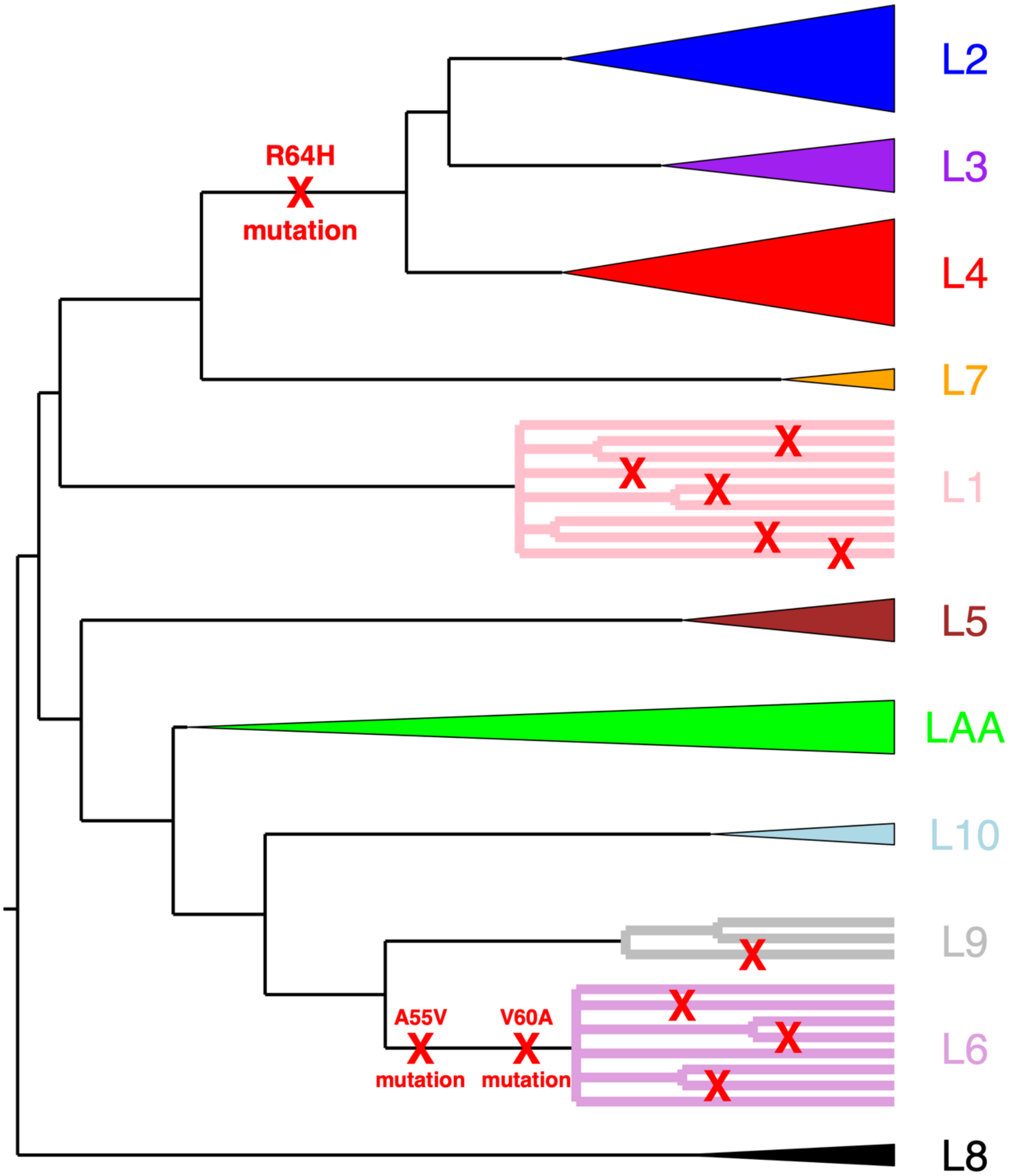

Supplementary Figure 24) ***zur*** **evolution across the MTBC.** Stylised phylogeny of the MTBC, showing all known lineages and the mutations which occur within *zur* on lineage defining branches. All sublineages aside from 1, 6 and 9 were collapsed.

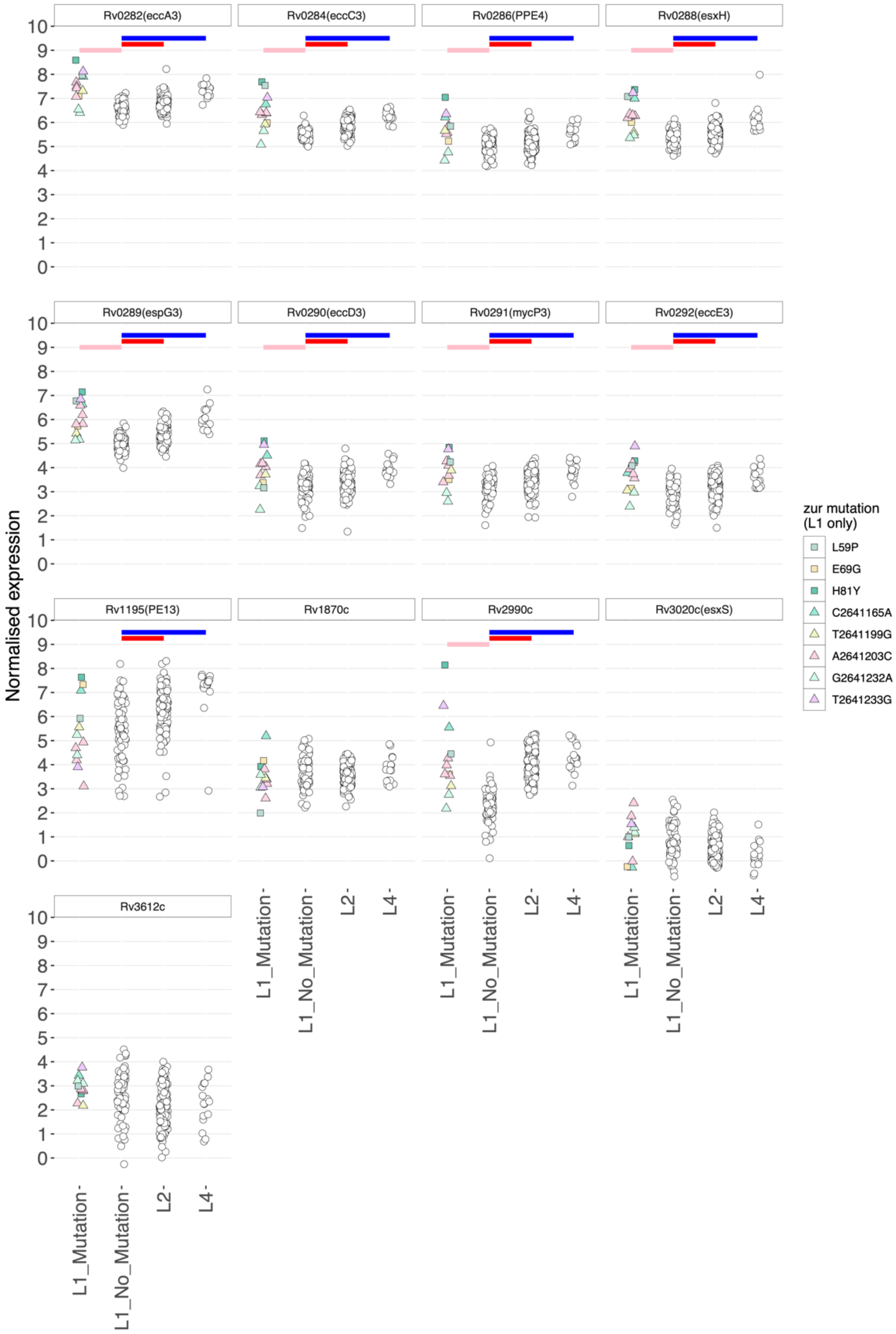

Supplementary Figure 25) *zur* regulon expression. Scatterplots compare the level of expression of 13 *zur* regulon genes between lineage 1 isolates with *zur* mutations, lineage 1 isolates without *zur* mutations, and isolates from lineages 2 and 4. Expression levels were averaged over strain replicates where available. Lineage 1 isolates with *zur* mutations are designated with points corresponding to the mutation they possess, and whether this mutation is an amino acid alternation in *zur* (squares), or a promotor region mutation (triangles). Pink, red and blue bars indicate statistically significant values for three comparisons: comparison 1: L1-mutant vs L1-non-mutant, comparison 2: L1-non-mutant vs L2 and comparison 3: L1-non-mutant vs L4, inferred using a one-sided Mann Whitney U Test.

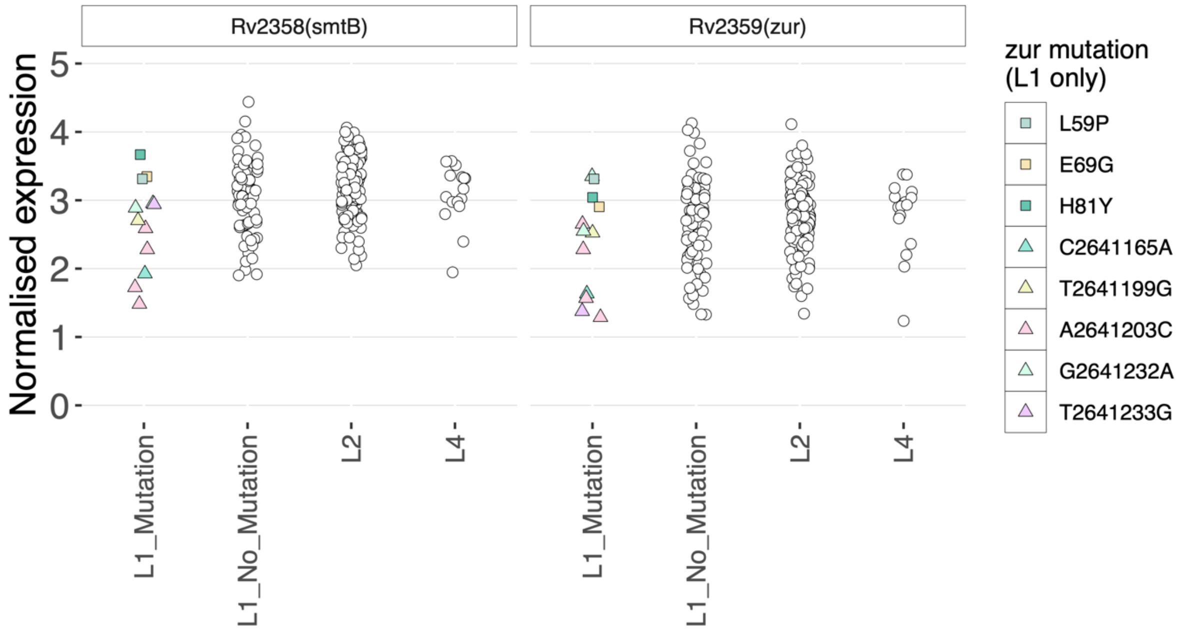

Supplementary Figure 26) *smtB-zur* operon expression. Scatterplots compare the level of expression of the *smtB* and *zur* genes between lineage 1 isolates with *zur* mutations, lineage 1 isolates without *zur* mutations, and isolates from lineages 2 and 4. Expression levels were averaged over strain replicates where available. Lineage 1 isolates with *zur* mutations are designated with points corresponding to the mutation they possess, and whether this mutation is an amino acid alternation in *zur* (squares), or a promotor region mutation (triangles).
