# Supplementary material for: Dormancy regulon reduction was pivotal to the evolution of Mycobacterium tuberculosis
Source: Nat Commun. 2026 Apr 13;17:5484. doi: 10.1038/s41467-026-71566-x (PMC13284343; doi:10.1038/s41467-026-71566-x)
Supplement: Supplementary file 3 — Transparent Peer Review file [file 41467_2026_71566_MOESM3_ESM.pdf]

nature portfolio

Peer Review File

Dormancy Regulon Reduction was Pivotal to the Evolution of M. tuberculosis

Corresponding Author: Dr Matthew Silcocks

This file contains all reviewer reports in order by version, followed by all author rebuttals in order by version.

Version 0:

Reviewer comments:

Reviewer #1

(Remarks to the Author)
This work principally describes results from in-silico analysis of three genome sets that indicate strong ongoing selection acting on the Rv0080 gene - previously reported to be part of the DosR-dependent dormancy regulon - within 4 of 5 L1 sublineages of the MTBC. In contrast, loss-of-function or putative functional mutations appear fixed in this gene at deep, lineage defining branch-levels for 8 other MTBC lineages. The authors analyze with great details the potential functional consequences of various non-synonymous mutations observed across the phylogeny, by using the predicted structure of the protein homodimer and FAD-binding domain of Rv0080 compared to the crystal structure of Fsq, one of its two homologues that is required for survival during hypoxia in M. smegmatis. Based on the tendency of mutations to predictably result in decreased protein stability or fall within the FAD-binding region, along with the detection of frameshift mutations in some parts of the phylogeny, it is inferred that Rv0080 is under selection for a loss or major negative alteration in protein function. Experimental data, obtained with M. smegmatis harboring a deletion of MSMEG_5136 (the closest gene homologue of Rv0080) and two L1 MTB clinical isolates containing one nonsense or one missense mutation in Rv0080, are used to conclude that the loss or the reduction of Rv0080 function is beneficial for survival during hypoxia, providing a selective advantage that likely contributed to the global spread of MTB.
This apparent strong positive selection within different L1 sublineages, contrasting with mutations differing between but fixed within most other MTBC lineages, is intriguing. However, I believe that the experimental data do not convincingly support the conclusion that the loss of Rv0080 confers a survival advantage in hypoxic conditions. Although the in-silico analysis is quite extensive and generally well-argued for this gene, many aspects of the interpretation remain speculative. This impression is even stronger for the part on zur, the other main gene under strong selection that is analyzed, for which no experimental results are provided. The latter gene, and Rv0042c that is more concisely considered in relation with Rv0080, were already found to be among the most prominent targets of positive selection in a global dataset of > 50,000 MTB genomes, in a previous landmark work published by Liu et al (Science, 2022).

Specific remarks are as follows.

1. In contrast to Δfsq, the difference in CFUs under hypoxic conditions between ΔMSMEG_5136 and wild-type M. smegmatis is only seen for two time points (624h and 720h) in Fig. 4c, without a higher survival of the deletion mutant at the final time point. The difference of about one log, at most, observed with three biological replicates at these two time points is not pronounced, especially considering some frequent limitations in the accuracy of mycobacterial CFU counting. The relatively large standard deviation observed at 720 h (with individual CFU values not shown) further raises concerns regarding the significance of the observed effect.
2. At days 18 and 24 in Fig. 4d — the time points used to infer an effect on hypoxia survival in MTB — the mean CFU counts for one or both MTB mutants are even closer to those of the wild-type strain, with substantial overlap in both individual replicate values and standard deviations, between the L121P mutant (day 18) or the frameshift mutant (day 24) and the wild-type. Moreover, the average CFUs are higher for the wild-type than for both mutants for the final timepoint (42 days), although the authors acknowledge that datapoints of the different samples overlapped in this case.
3. Furthermore, complementation of the mutant background with a wild-type gene copy was not performed - neither for ΔMSMEG_5136 in M. smegmatis, nor for the Rv0080 L121P or Rv0080 frameshift M. tuberculosis isolates - in order to assess restoration of the wild-type survival phenotype, as would be required for confirmation according to the molecular Koch's postulates. This would be especially important for the non-isogenic MTB isolate set, in which the L121P or frameshift isolates are separated by at least 108 SNPs and 129 SNPs from the Rv0080 wild-type isolate, respectively (with potentially

further structural or repetitive region changes undetectable by Illumina sequencing, which cannot be excluded).
4. The Results section on zur reads as a particularly long descriptive list of mutations and speculative functional interpretations, and seems to offer limited meaningful insights. Given the lack of changes in residues predicted to be functionally or structurally relevant by the models that were used, a number of alternative hypotheses put forward appear somewhat basic in my view. Examples are “Despite this, we predict that changes to the most frequently mutated residue, R10 (five mutations) may influence DNA binding. Arginine is one of the most favoured residues for DNA binding. Its substitution to histidine (four of the 5 mutations), results in the replacement of a positively charged amino acid with one that has a pH sensitive side chain, and may alter the role of this residue within zur” (lines 632-636), and “Notably the cysteine-rich zinc binding site (Site A; predicted residues: 650 85, 88, 125 and 128), which is also crucial for dimer formation, did not evolve any mutations (Figure 5c). We hypothesise that the remaining variants may have less obvious effects on the protein, but could destabilise overall protein structure (e.g. V106, H110, S113, H117 are near a predicted dimer interface) or affect the translation or folding of zur” (lines 650-654).
5. I was also disappointed by the discussion for similar reasons. As indicated above, the strong apparent selection acting on Rv0080 (and zur and Rv0042c, but see related comment below) in L1 in contrast to fixed mutations in most other lineages seems one central point. However, the potential explanation that is offered is essentially limited to the hypothesis “that the Rv0080 and zur mutations now fixed within ‘modern’ lineages confer important functional changes to these proteins, and that ‘ancient’ lineages are undergoing convergent evolution to emulate these changes” (lines 743-746). I would expect further development.
6. Along the same lines, while this is highlighted as a key finding (lines 732-737), the observation that diverse forms of variation - SNVs in promoter regions or frameshift-causing indels in CDSs in addition to coding-regions SNVs - via which selection can act on a gene target should not be a surprise at all. As a prominent illustration, it is very well known that these three forms of variations are main selective forces acting on drug resistance genes in MTB (e.g. indels in pncA CDS for PZA, indels in katG for INH, C-15T and T-8A in the fabG1 promoter region for INH, C-12T in the eis promoter region for KAN, etc). And in Liu et al’s work for instance, promoter and intergenic regions enriched for mutations in MTB genomes were well screened for and readily captured, as seen in Fig. 1B in their paper.
7. Although not discussed in the manuscript, the finding that Rv0080, zur, and Rv0042c show strong positive selection in L1 genomes here, yet only zur and Rv0042c appear among the top 30 dN/dS scores in the much larger global dataset from Liu et al. (as seen in Fig.S1 in Supplementary Material of that paper), also raises questions. Even if Rv0080, zur, and Rv0042c are under extreme selection specifically in L1.1.1, L1.1.2, L1.1.3 and L1.2.2 genomes according to the present work, the Liu’s dataset comprises 3,700 of these L1 sublineages (as per the data in 51229_Mtb_Seq_Info.txt, retrieved from Supplementary Materials). Therefore, I would have expected to find Rv0080 in the largest set too.
8. Lines 563-566 in Results, 765-767 in Discussion, and 939-940 in Methods. Although the fsq gene is “intact” in M. canettii strain 140070008, it is frameshifted similarly to M. tuberculosis H37Rv in other publicly available genomes of M. canettii strains, such as 140010059 and 140060008, indicating that the frameshift-inducing mutation predates the emergence of M. tuberculosis from M. canettii.

Reviewer #2

(Remarks to the Author)
The manuscript by Silcocks et al. provides a compelling analysis of ongoing positive selection in the Mycobacterium tuberculosis complex (MTBC), leveraging a large dataset of Thai genomes to identify three loci under strong diversifying selection: a dormancy gene (Rv0080), a zinc homeostasis gene (zur), and a virulence-associated gene (ESX1). Focusing on Rv0080, the authors robustly demonstrate repeated reductive evolution of this dormancy survival regulon across the MTBC phylogeny, particularly in the ancestral L1 lineage, and elucidate mutation-driven changes in molecular secondary structure, dimer stability, and hypoxia survival, validated through ΔRv0080 mutant and wild-type M. smegmatis comparisons, as well as L1 MTBC strain analyses.

I have several critiques. First, the use of a 'flat' dN/dS ratio to identify candidate genes is a limitation. More sophisticated models, such as those implemented in PAML, which partition proteins into fractions experiencing negative, neutral, or positive selection, better reflect complex biological dynamics. The current approach likely captures only extreme cases of positive selection, potentially overlooking genes under localized selection pressure, such as virulence factors with dN/dS ratios near 1 despite positive selection. Incorporating such models, though computationally intensive, would strengthen the findings. Second, the manuscript lacks discussion of the temporal dynamics and genetic diversity within MTBC lineages. Older lineages, like L1, naturally accumulate more non-synonymous mutations than younger ones, such as L2, which likely emerged during domestication in China. Recent population bottlenecks in modern lineages could also accelerate fixation of Rv0080 mutations (e.g., frameshifts or deficient mutants), a point that warrants mention. Third, the discussion of the non-replicative state during dormancy (line 234) overlooks ongoing debates, with studies from Sarah Fortune and others challenging this paradigm, which deserves acknowledgment to contextualize the findings. Finally, Figure S1’s gene labels in panels a and b are nearly illegible due to small font size, hindering interpretation.

This manuscript’s key contribution lies in highlighting how MTBC’s population genetic structure shapes distinct adaptive landscapes across lineages and sublineages, driven by epistatic interactions. These insights underscore the need for precision medicine in tuberculosis treatment, offering a path toward tailored therapies and a deeper understanding of global TB dynamics. I recommend acceptance for publication in Nature Communications after addressing these concerns to enhance methodological rigor and clarity.

Version 1:

Reviewer comments:

Reviewer #1

(Remarks to the Author)
I appreciate that many of my comments have been addressed, substantially improving the manuscript. However, I continue to have significant concerns regarding the reproducibility and interpretation of some previous or new results from hypoxia survival experiments, which constitute a major experimental support for the conclusion on reductive evolution of the dormancy regulon. I also have additional comments that were not included in my initial review, given the number and nature of concerns that were raised at that stage.
1. Lines 618-627 in Results and 907-909 in Discussion. The conclusion that the mutations V60G and V31G that evolved along deep, lineage defining branches of the Mtb phylogeny result in enhanced survival phenotypes in M. smegmatis is not robustly supported, when the results of the replicate datasets in Figure 4e and Supplementary Figure 19 are examined closely.
In Supplementary Figure 19, V60G appears to display a substantial survival advantage over both the wild type and ancestral alleles from day 5 to 10. In contrast, in Figure 4e, this survival advantage is, at best, limited to a ~1-log difference on day 7 only. The mean difference on day 14 is only ~0.5 log and likely within overlapping standard deviations (which are not shown).
Conversely, while the survival advantage of V31G over the wild type and ancestral alleles appears substantially higher (~1-log) on both day 7, 10 and 14 in Figure 4e, the supplemental data show a 1-log difference only on a single time point (day 6). In this figure, a substantial difference at day 7 is seen only with the ancestral allele, but these ancestral datapoints fall even below the wild type values and are rather suggestive of outliers than a reproducible biological effect. There is no difference at all between the V31G, wild type and ancestral alleles at day 10.
2. Lines 583-598 in Results. As indicated in my previous comments, the mean differences in survival under hypoxia between the tested non-isogenic Rv0080 "mutant" and "wild-type" M. tuberculosis isolates reported in Supplementary Figure 18 are small, mostly below 1-log CFU. Moreover, overlapping datapoints have to be acknowledged between the wild type and both mutants for day 18, and the wild type and the frameshift mutant for day 24, and not only for day 42, where the wild type appears to survive even better than the mutants. Even if presented as a tentative interpretation, these experimental data do not provide convincing support for the hypothesis that loss or alteration of Rv0080 function confers a survival advantage in a native (albeit non-isogenic) M. tuberculosis background.
3. Lines 491-501 in Results. As the text stands, the speculation that the lack of selection for Rv0080 in lineages 2, 4 and 6 can be explained via the loss-of function mutations acquired on their lineage-defining branches may appear contradictory with the presence of an additionally acquired premature stop codon in Rv0080 in a large L2 clade of 22 Thai isolates from the Pan-Asia dataset. This might on the contrary be interpreted as evidence for still ongoing selection for loss-of-function in L2. A clearer possible explanation could be that the presence of this non-sense mutation might reflect further pseudogenization of Rv0080 due to drift, after the acquisition of the LoF mutation at lineage-defining branch level. However, see the comments above on V60G hypoxia survival phenotype in M. smegmatis.
4. Lines 494-497 in Results. It should be confirmed whether all L2 & L4 genomes from the Pan-Asia dataset and all 277 L6 genomes carry the V60G and V31G mutations, as these were inferred to be have been acquired at their respective lineage-defining-branch levels based on the LAA-Z dataset.
5. Lines 503-514 in Results. As overexpression of the transcription factor Rv0042c is a main driver of Rv0079-Rv0080 expression, is there any indication that NS mutations in Rv0042c might be loss-of-function variants, in order to support the conclusion that extreme signal of L1-specific positive selection observed for Rv0042c may represent a mechanism of reducing or eliminating the expression of Rv0080?
6. Lines 844-848 in Discussion. The fact that indels are never observed in phoR is fully expected, as the selective pressure acting on phoR is opposite compared to Rv0080: loss-of-function mutations are counter-selected because phoR is required for virulence in vivo.
7. Lines 913-918 in Discussion. "…there doesn't seem to be an obvious phenotypic advantage for a frameshift mutation, as suggested by the hypoxia survival data (Figure 4d; Supp Figures 18 and 19), and lack of ongoing selection for subsequent mutations amongst L2 and L4 strains. We speculate that, like zur, there could be a fitness trade-off, whereby a partly functional version of Rv0080 could be beneficial during some stages of infection."
This appears as an overinterpretation, as it is unknown whether the substitution mutations tested for hypoxia survival do not result in complete loss-of-function of Rv0080 too, as is expectedly the case with the gene deletion or frameshift mutations.
8. Lines 114-124 in Introduction. "To help explain the differences in phenotypic manifestations between lineages, we recently proposed patterns of East Asian Mtb diversity to reflect the 'Two Layer' admixture model (Silcocks & Dunstan, 2023). Under this model, … Our Neolithic Mtb origin model provides a theoretical explanation for long-standing observations that 'evolutionarily ancient' lineages (lineages 1, 5, 6, 7, 8, 9 and 10) display lower levels of virulence and are more likely to result in latent infection than their 'modern' counterparts".
Actually, it is a paleolithic model that is strongly advocated for by Silcocks et al., as illustrated by the following sentence in their discussion: "We wish to note that our model of historic human and Mtb co-expansion is one which infers a Paleolithic origin of contemporary Mtb variation."

Reviewer #2

(Remarks to the Author)
I have reviewed the revised manuscript and the authors' point-by-point responses. The changes implemented fully address my previous comments on selection issues, as well as those of the second reviewer, resulting in a improved paper with

enhanced clarity, rigor, and completeness.

I recommend acceptance of the manuscript in its current form.

Open Access This Peer Review File is licensed under a Creative Commons Attribution 4.0 International License, which permits use, sharing, adaptation, distribution and reproduction in any medium or format, as long as you give appropriate credit to the original author(s) and the source, provide a link to the Creative Commons license, and indicate if changes were made.
In cases where reviewers are anonymous, credit should be given to 'Anonymous Referee' and the source.
The images or other third party material in this Peer Review File are included in the article's Creative Commons license, unless indicated otherwise in a credit line to the material. If material is not included in the article's Creative Commons license and your intended use is not permitted by statutory regulation or exceeds the permitted use, you will need to obtain permission directly from the copyright holder.
To view a copy of this license, visit https://creativecommons.org/licenses/by/4.0/

Reviewer #1 (Remarks to the Author):

This work principally describes results from in-silico analysis of three genome sets that indicate strong ongoing selection acting on the Rv0080 gene - previously reported to be part of the DosR-dependent dormancy regulon - within 4 of 5 L1 sublineages of the MTBC. In contrast, loss-of-function or putative functional mutations appear fixed in this gene at deep, lineage defining branch-levels for 8 other MTBC lineages. The authors analyze with great details the potential functional consequences of various non-synonymous mutations observed across the phylogeny, by using the predicted structure of the protein homodimer and FAD-binding domain of Rv0080 compared to the crystal structure of Fsq, one of its two homologues that is required for survival during hypoxia in M. smegmatis. Based on the tendency of mutations to predictably result in decreased protein stability or fall within the FAD-binding region, along with the detection of frameshift mutations in some parts of the phylogeny, it is inferred that Rv0080 is under selection for a loss or major negative alteration in protein function.

Experimental data, obtained with M. smegmatis harboring a deletion of MSMEG_5136 (the closest gene homologue of Rv0080) and two L1 MTB clinical isolates containing one nonsense or one missense mutation in Rv0080, are used to conclude that the loss or the reduction of Rv0080 function is beneficial for survival during hypoxia, providing a selective advantage that likely contributed to the global spread of MTB.

This apparent strong positive selection within different L1 sublineages, contrasting with mutations differing between but fixed within most other MTBC lineages, is intriguing. However, I believe that the experimental data do not convincingly support the conclusion that the loss of Rv0080 confers a survival advantage in hypoxic conditions. Although the in-silico analysis is quite extensive and generally well-argued for this gene, many aspects of the interpretation remain speculative. This impression is even stronger for the part on zur, the other main gene under strong selection that is analyzed, for which no experimental results are provided. The latter gene, and Rv0042c that is more concisely considered in relation with Rv0080, were already found to be among the most prominent targets of positive selection in a global dataset of > 50,000 MTB genomes, in a previous landmark work published by Liu et al (Science, 2022).

As our responses below indicate, we're very grateful to Reviewer 1 for their detailed and insightful commentary on our work. We agree that the functional insights offered into *Rv0080* and *zur* by the previous iteration of our manuscript were somewhat limited, and have completed new experiments and bioinformatic analyses to address this. This includes new hypoxia experiments for *MSMEG_5136* and *Rv0080*, in addition to an analysis of RNA-sequencing data for a subset of our Vietnamese clinical strains for *zur*.

For our hypoxia testing, we have completed a new experiment which includes complementation, and expands the scope of the analysis to demonstrate the functional relevance of *Rv0080* mutations which evolve across the deep branches of the MTBC phylogeny, thus supporting a key premise of our paper. In line with their comments regarding the study of Liu et al. (2022), we have re-analysed this dataset to show that

Rv0080 is under selection when screening the lineage 1 isolates it contains, thus addressing their concern regarding the apparent lack of selection for this gene.

Our manuscript also now offers key insight into the evolution of *zur*, which we've obtained both via our analysis of RNA-sequencing data, and re-analysis of the dataset of Liu et al. (2022). Most importantly, our analysis of RNA-seq data ascribes a functional role to *zur* mutations: demonstrating both amino acid and promoter region mutations in L1 strains to lead to consistent upregulation of the *zur* regulon, and expression levels more typical of L2 and L4 strains. To complement this result, we demonstrate the signal of selection for *zur* inferred by Liu et al. (2022) is driven overwhelmingly by isolates from lineage 1, ranking as the strongest target of selection in a L1-specific screen, yet outside the top 100 genes for L2 and top 80 for L4. These and other analyses are detailed more thorough in the point-by-point responses below.

Specific remarks are as follows.

1. In contrast to Δfsq, the difference in CFUs under hypoxic conditions between ΔMSMEG_5136 and wild-type M. smegmatis is only seen for two time points (624h and 720h) in Fig. 4c, without a higher survival of the deletion mutant at the final time point. The difference of about one log, at most, observed with three biological replicates at these two time points is not pronounced, especially considering some frequent limitations in the accuracy of mycobacterial CFU counting. The relatively large standard deviation observed at 720 h (with individual CFU values not shown) further raises concerns regarding the significance of the observed effect.

We agree with the reviewer regarding the lack of a pronounced survival phenotype in our previous hypoxia survival assay. To address this, we have performed additional hypoxia survival experiments involving *M. smegmatis* with modified experimental protocols (**Results lines 562-581, and Methods lines 1129-1154**).

The primary change in method is inoculation of the culture at a higher OD, i.e. at 0.1 as opposed to 0.005 in prior experiments. The effect this has is that oxygen is depleted more quickly from the serum vial, allowing less time for *M. smegmatis* to adapt, leading to more pronounced survival defects for genes involved in hypoxic adaptation. Under these revised experimental conditions, Δ*MSMEG_5136* displayed a survival advantage exceeding an order of magnitude relative to the wild type at three timepoints (days 7, 10 and 14), with the day 14 values exceeding two orders of magnitude (Figure 4c). Consistent with increased hypoxic stress under these revised conditions, the Δ*dosR* mutant reached the lower limit of detection by day 10 (Figure 4c). As outlined below (see comment 3), we have now complemented this phenotype using *MSMEG_5136*, which results in a reduction of hypoxia survival equivalent to wild type levels (Figure 4c). We note that a replicate of this experiment, performed in duplicate, also yielded similar results (Supp Figure 19). In conclusion, a revised experimental protocol and complementation demonstrated that the loss of the *Rv0080*'s *M. smegmatis* orthologue, *MSMEG_5136*, results in a hypoxia survival advantage.

2. At days 18 and 24 in Fig. 4d — the time points used to infer an effect on hypoxia survival in MTB — the mean CFU counts for one or both MTB mutants are even closer to those of the wild-type strain, with substantial overlap in both individual replicate values and standard deviations, between the L121P mutant (day 18) or the frameshift mutant (day 24) and the wild-type. Moreover, the average CFUs are higher for the wild-type than for both mutants for the final timepoint (42 days), although the authors acknowledge that datapoints of the different samples overlapped in this case.

We agree with the reviewer that our *Mtb* hypoxia survival experiment doesn't result in a strong survival phenotype for mutant strains. This outcome is likely related to the differences in genetic backgrounds between these clinical strains mentioned by the reviewer.

To address this, we have performed a new experiment in which we complement the *M. smegmatis* Δ*MSMEG_5136* mutant with key *Rv0080* alleles selected from across our genomic datasets (**Results lines 600-627 and Methods lines 1129-1154**). These include the ancestral *Rv0080* allele (G60V applied to the H37Rv sequence), the V60G allele present in isolates from lineages 2, 3, 4 and 7 (i.e. the H37Rv sequence), the lineage 6 allele (V31G added to this ancestral background (i.e. V31G + G60V to the H37Rv sequence)), a frameshift mutant (on the ancestral background), and two mutations commonly found amongst Vietnamese lineage 1 strains (L121P and V49G on ancestral backgrounds). The inclusion of these additional, lineage-defining mutations (V60G and V31G) allowed us to expand the scope of the experiment to test our hypothesis that mutations which evolve on the lineage-defining branches of the MTBC phylogeny confer enhanced hypoxia survival for these lineages.

As we outline in the Results (**lines 605-608**), when expressed from a native promoter as a *Rv0079-Rv0080* operon, the ancestral allele complemented Δ*MSMEG_5136*. As we now outline (**lines 610-627**), each of the *Rv0080* mutations we tested, when expressed in Δ*MSMEG_5136*, displayed enhanced hypoxia survival relative to the ancestral allele.

The three variants selected from clinical Vietnamese strains (L121P, V49G and the frameshift mutation) all either failed to or only partially complemented Δ*MSMEG_5136*, with between 10^3 and 10^4 viable cells remaining at day 14, and roughly one log survival advantage or more at days 7 and 10 (Figure 4d). The two lineage defining mutations we tested (V60G and V31G), also displayed a survival advantage relative to the ancestral allele. Both displayed an approximate survival advantage of one log at day 7, and the V31G strain also displayed a log survival advantage at day 10. Both strains displayed roughly 10^3 to 10^4 viable cells on day 14 (Figure 4e). Again, these results were consistent with the repeat of this experiment, which suggested a higher survival advantage of the V60G strain (Supp Figure 19). In conclusion, positively selected *Rv0080* variants when expressed in *M. smegmatis* model demonstrate a survival advantage under hypoxia.

3. Furthermore, complementation of the mutant background with a wild-type gene copy was not performed - neither for ΔMSMEG_5136 in M. smegmatis, nor for the Rv0080 L121P or Rv0080 frameshift M. tuberculosis isolates - in order to assess restoration of the wild-type

survival phenotype, as would be required for confirmation according to the molecular Koch's postulates. This would be especially important for the non-isogenic MTB isolate set, in which the L121P or frameshift isolates are separated by at least 108 SNPs and 129 SNPs from the Rv0080 wild-type isolate, respectively (with potentially further structural or repetitive region changes undetectable by Illumina sequencing, which cannot be excluded).

As mentioned above (comment 1), in our new hypoxia experiment, we have complemented the *M. smegmatis* Δ*MSMEG_5136* mutant, demonstrating a reduction of survival to wild-type levels (Figure 4c). We were also able to complement the *M. smegmatis* Δ*MSMEG_5136* with different *Rv0080* variants (see comment 2 above).

4. The Results section on zur reads as a particularly long descriptive list of mutations and speculative functional interpretations, and seems to offer limited meaningful insights. Given the lack of changes in residues predicted to be functionally or structurally relevant by the models that were used, a number of alternative hypotheses put forward appear somewhat basic in my view. Examples are "Despite this, we predict that changes to the most frequently mutated residue, R10 (five mutations) may influence DNA binding. Arginine is one of the most favoured residues for DNA binding. Its substitution to histidine (four of the 5 mutations), results in the replacement of a positively charged amino acid with one that has a pH sensitive side chain, and may alter the role of this residue within zur" (lines 632-636), and "Notably the cysteine-rich zinc binding site (Site A; predicted residues: 650 85, 88, 125 and 128), which is also crucial for dimer formation, did not evolve any mutations (Figure 5c). We hypothesise that the remaining variants may have less obvious effects on the protein, but could destabilise overall protein structure (e.g. V106, H110, S113, H117 are near a predicted dimer interface) or affect the translation or folding of zur" (lines 650-654).

We thank the reviewer for this commentary on our *zur*-related findings, and agree that the previous iteration of this analysis did not reach a definitive conclusion regarding the role of the mutations observed. We have been able to gain insight into the functional role of *zur* mutations using RNA-seq data from a subset of our Vietnamese strains, which was recently made available via a manuscript published in Cell (Culviner et al. 2025). We show using this data that both amino acid alterations in *zur*, and mutations in the *smtB*/*zur* promoter region lead to upregulation of the *zur* regulon, resulting in expression levels similar to strains from lineages 2 and 4. These Results are detailed in **lines 764-796** and Methods **lines 1281-1296**.

To summarise, our protocol involved screening the RNA-sequencing dataset of Culviner et al. (2025) for isolates with *zur* mutations (either amino acid altering, promoter or indels), and comparing expression levels between mutant and non-mutant L1 strains. As noted, we restricted this comparison to the 13 *zur* regulon genes (defined by Maciag et al. 2007) with strongly concordant expression levels between sample replicates. As we note, L1 strains with *zur* mutants displayed significantly higher expression levels for 9 of these 13 genes, and levels similar to L2 and L4 strains. Certain *zur* amino acid and promoter mutations (i.e. H81Y and T2641233G) led to consistently higher expression levels across genes. We further report that *zur* promoter mutants have consistently lower expression of the *smtB*/*zur* operon itself, whereas strains with amino acid mutations display expression levels typical of non-mutant L1 strains.

To shed further light on the precise mechanisms via which mutations in *zur* inhibit its function, we have expanded the scope of our bioinformatic screen to incorporate the data of Liu et al. (2022) (**lines 752-758**), and include a more comprehensive synthesis of the mechanisms of disruption implicated, using *Rv0080* and *Rv0678* as illustrative examples (**lines 727-740**).

To summarise, we failed to notice a significant enrichment of mutations in DNA-binding or DNA-interacting residues in the dataset of Liu et al. (2022), and conclude that mutations likely inhibit *zur* function via multiple independent mechanisms. In support of this conclusion, we've referenced the example of *Rv0080*, and the study of Sonnenkalb et al. (2023), who infer mutations in the drug resistance target *Rv0678* disrupt function via multiple diverse mechanisms, including decreasing stability, DNA binding, ligand binding and dimerization. We conclude that similar mechanisms may be at play when considering *zur*, which would explain why DNA-interacting or binding residues don't display a strong enrichment of mutations in either the Vietnamese dataset or the dataset of Liu et al. (2022).

In addition to these revisions, we have streamlined the Results section focused on *zur* for brevity (**see line ranges 673-699, 716-725 and 731-740**), and expanded our Discussion to consider the evolutionary significance of an alteration of *zur* function across the MTBC phylogeny (see Comment 5).

5. I was also disappointed by the discussion for similar reasons. As indicated above, the strong apparent selection acting on Rv0080 (and zur and Rv0042c, but see related comment below) in L1 in contrast to fixed mutations in most other lineages seems one central point. However, the potential explanation that is offered is essentially limited to the hypothesis "that the Rv0080 and zur mutations now fixed within 'modern' lineages confer important functional changes to these proteins, and that 'ancient' lineages are undergoing convergent evolution to emulate these changes" (lines 743-746). I would expect further development.

We thank the reviewer for highlighting the lack of detail in our Discussion exploring the lineage-specific patterns of selection we observe for our key gene hits. Given we have now obtained functional data for both *Rv0080* and *zur*, we have been able to expand the scope of this Discussion to draw key conclusions about the phenotypic consequences of mutations in these genes, and the patterns of convergence towards these phenotypes across the MTBC phylogeny.

We now outline the data we've obtained demonstrating the *Rv0080* mutation fixed in modern lineages (V60G) confers a hypoxia survival advantage relative to the ancestral version of this gene, and that mutations observed in L1 and across the MTBC phylogeny more broadly result in convergence towards a similar survival advantage (**lines 905-909**). Similarly, for *zur*, we show mutations occurring amongst L1 isolates (both promoter and amino acid) lead to upregulation of the *zur* regulon, and levels of expression similar to lineages 2 and 4 strains (**lines 764-796**). Although unlike *Rv0080*, we don't have direct evidence that the mutation fixed in modern lineages (R64H) is solely responsible for the

upregulation observed in L2 and L4, we can conclude that the mutations which evolve in L1 strains result in convergence towards the same phenotype.

Our Discussion now also includes a more detailed synthesis of what the bioinformatic data available for these genes indicates about the relative strength of selection for a complete loss versus a change in function of these genes (**see lines 874-885 and 909-918**). We infer based on the lack of enrichment of *zur* mutations in conserved residues and loss of function mutations in any global lineages, that the strength of selection for a complete loss of *zur* function isn't as strong as for a loss of *Rv0080* function. We conclude that retaining some degree of *zur* function may therefore be more beneficial than evolving a complete loss of function, possibly via retention of the ability to toggle between zinc-conserving and zinc-scavenging states. We acknowledge that there is still functional work to be done to thoroughly explore these genes, however believe we provide a strong genetic and evolutionary foundation upon which to do so.

6. Along the same lines, while this is highlighted as a key finding (lines 732-737), the observation that diverse forms of variation - SNVs in promoter regions or frameshift-causing indels in CDSs in addition to coding-regions SNVs - via which selection can act on a gene target should not be a surprise at all. As a prominent illustration, it is very well known that these three forms of variations are main selective forces acting on drug resistance genes in MTB (e.g. indels in pncA CDS for PZA, indels in katG for INH, C-15T and T-8A in the fabG1 promoter region for INH, C-12T in the eis promoter region for KAN, etc). And in Liu et al's work for instance, promoter and intergenic regions enriched for mutations in MTB genomes were well screened for and readily captured, as seen in Fig. 1B in their paper.

We thank the reviewer for pointing out the parallels between the mechanisms of evolution impacting our target genes and those associated with drug resistance. Our objective in this part of the Discussion was not to claim the novelty of this finding, but rather to note an observation we made regarding approaches typically used to screen for selection in *Mtb*, and suggest avenues for future insights. We have now expanded this section of the Discussion to better articulate this (**lines 825-848**).

As we now mention, we've noticed that most screens for selection in *Mtb* will do so from the perspective of coding region SNPs only (i.e. Wilson et al. 2020; Chiner-Oms et al. 2019), or if they do also screen for intergenic mutations (i.e. Liu et al. 2022; Chiner-Oms et al. 2022), will do so in an independent screen. Virtually all prior screens (a notable exception being Vargas et al. 2023), will systematically exclude indels as part of their variant calling pipeline. As we now mention, screening for all three forms of variation (coding region SNPs, indels and promoter mutations), will both boost power for the detection of gene hits and provide insight into the change in function being selected for. The evidence we have for selection acting on *zur*, for example, is bolstered when we consider the combined sum of lineage 1 isolates which carry non-synonymous mutations (50/643; 7.78%), promoter region mutations (54/643) and coding region indels (4/643). Additionally, when formulating hypotheses about the role of selection, we can infer from the nine lineage 1 isolates which carry frameshift inducing indels in *Rv0080*, that a complete loss of function is both tolerated and under selection.

The study of Liu et al. (2022) mentioned by the reviewer actually provides an interesting case in point. While *zur* emerges as a gene with one of the highest SNV densities (Figure 1b, main barplot), it doesn't appear in the plot of the top-ranking intergenic regions (Figure 1b, inset), and these two forms of evidence aren't reconciled with one another. Perhaps a future study could expand the 'mutation density' statistic depicted in Figure 1b of Liu et al. (2022) (which simply divides the sum of NS and S mutations by that gene's length; see 'Mutation burden test' in their Supp.), to include two additional categories of mutations: indels, and promoter region mutations (with adjustment of the denominator to include the length of the promoter region considered). Using this approach, *zur* may well have risen to attain a higher rank in this screen and emerged as a gene to prioritise in subsequent analysis.

7. Although not discussed in the manuscript, the finding that Rv0080, zur, and Rv0042c show strong positive selection in L1 genomes here, yet only zur and Rv0042c appear among the top 30 dN/dS scores in the much larger global dataset from Liu et al. (as seen in Fig.S1 in Supplementary Material of that paper), also raises questions. Even if Rv0080, zur, and Rv0042c are under extreme selection specifically in L1.1.1, L1.1.2, L1.1.3 and L1.2.2 genomes according to the present work, the Liu's dataset comprises 3,700 of these L1 sublineages (as per the data in 51229_Mtb_Seq_Info.txt, retrieved from Supplementary Materials). Therefore, I would have expected to find Rv0080 in the largest set too.

We thank the reviewer for drawing the interesting comparison between our selective screen and that of Liu et al. (2022). We have reanalysed the data used by Liu et al. (2022) to demonstrate that *Rv0080* is under selection in the lineage 1 isolates they analyse, and does in fact fall within the top 30 genes when screening in a lineage-specific fashion. We have added this data to our manuscript (**Results lines 516-531 and Methods lines 1255-1268**), as we believe it adds additional evidence and perspective to our inferences of selection in *Rv0080*. Below we outline the approach taken for this analysis, our results, and additional notes on key differences in the comparative datasets and screening approaches between our analysis and that of Liu et al. (2022).

The approach of Liu et al. (2022)
As we explain, Liu et al. (2022)'s analysis of unfixed mutations (featuring in their Figure 1b and Figure S1) identifies genes with a high density of sites at which sequencing reads support both the alternate and reference allele for a single isolate. Their approach therefore screens for selection acting on a very recent timescale (i.e. mutations in the process of becoming fixed, and not yet represented on the terminal branches of the phylogeny). Our approach, conversely, captures mutations on both the internal and terminal branches of the phylogeny, and provides insight into evolution on a much deeper timescale.

The dataset of Liu et al. (2022)
When considering dataset sizes; although their approach screens 51,229 isolates, only 33,741 actually have one or more unfixed mutations, and 221,853 unfixed mutations are found across the entire dataset after filtering. In contrast, despite consisting of fewer samples (2,506), we identify over 175,000 mutations across the evolutionary history of our Vietnamese isolates (when combining lineages 1, 2 and 4).

Another key difference influencing the inferred rate of selection acting on *Rv0080* in these two datasets is the comparative frequency of lineage 1. Lineage 1 isolates constitute 25.7% of our Vietnamese dataset (643/2,506), yet only 9.3% (4,762/51,229) of the dataset of Liu et al. (2022). As we show the signal of selection for *Rv0080* to be restricted to lineage 1 isolates, the proportion of L1 in the dataset will naturally affect whether is stands out when screening all lineages at once. For example, *Rv0080*'s dN/dS ratio in our Vietnamese dataset is 9.37 when screening lineage 1 isolates only (Supp Figure 3), yet only 2.66 in our combined screen of lineages 1, 2 and 4 (Figure 2b). Nevertheless, we were interested to explore the perspective the dataset of Liu et al. (2022) offered into the evolution of *Rv0080*, and present the results of this analysis below.

Reanalysing the dataset of Liu et al. (2022)

We downloaded the unfixed mutation data of Liu et al. (2022), which the authors have generously made publicly available (file 'All_unfixed_mutation.txt' at 'https://github.com/MtbEvolution/resR_Project'). As detailed in our Methods (**lines 1255-1268**), we filtered this file to mutations with frequency between 1 and 90% (as reported in their manuscript), resulting in their final set of 221,853 mutations. For consistency with the parameters of our screen, we filtered out mutations within poorly mappable regions of the genome using the coordinates of Marin et al. (2022), leaving 214,424 mutations. We also chose to rank genes by the density of non-synonymous mutations, as opposed to the sum of non-synonymous and synonymous mutations used by Liu et al. (2022). Although the use of this metric results in nearly indistinguishable gene rankings to Liu et al. (2022) when screening all lineages (Figure R1), we found it better allows us to identify targets of selection when screening the smaller, more noisy lineage-specific datasets (where the chance accumulation of synonymous mutations can result in a gene attaining a high rank whilst possessing a relatively low dN/dS ratio). In Figure R1, we plot the genes with the top 30 non-synonymous mutation densities, in the same style as Figure 1b of Liu et al. (2022). All 30 match the top 30 genes of Liu et al. (2022), with minor differences in ordering due to differences in the filtering and ranking approaches used. As noted by the reviewer, *Rv0080* does not appear in the top 30 genes, ranking as gene number 140.

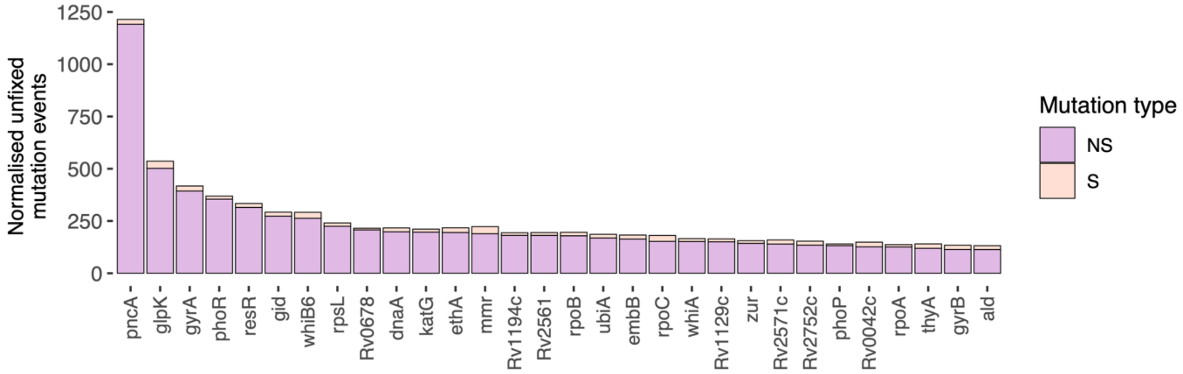

Figure R1) Barplot showing the mutational density per gene inferred from the unfixed mutation dataset of Liu et al. (2022). Mutational densities were calculated when considering mutations identified across all lineages, and genes are ranked based off the density of non-synonymous mutations. The genes with the top 30 highest NS mutation densities are shown.

Conducting a lineage-specific screen for selection using the Liu et al. (2022) dataset

Using the lineage calls made available in file '51229_Mtb_Seq_Info.txt', from the same GitHub link referenced above, we were able to screen for selection in a lineage-specific fashion. We subset the 214,424 mutations mentioned above to those occurring amongst the 4,762 lineage 1 isolates in this list, resulting in a total of 18,651 coding-region mutations for subsequent analysis. When calculating the NS mutation density for lineage 1 isolates only, *Rv0080* rose to attain the 21st highest value (Supp Figure 16).

Although appearing in the top 30 genes, it's notable that the signal of selection observed for *Rv0080* is not as prominent as we infer from our analysis of fixed mutations within the Vietnamese dataset (where *Rv0080* ranks 2nd; Supp Figure 3). As we illustrate (**Results lines 516-531**), this can be explained by the high rates of *Rv0080* mutations already fixed in some L1 sublineages. We show using the Pan-Asia *Mtb* dataset that 73% and 76% of isolates from sublineages L1.1.2 and L1.1.3 already possess non-synonymous mutations in *Rv0080* (Supp Table 1), and will therefore be under reduced pressure to evolve additional mutations. The lack of ongoing selection pressure for *Rv0080* in these sublineages is reflected in the data of Liu et al. (2022), where *Rv0080* ranks as the gene with the 308th and 3970th highest NS mutation density for L1.1.2 and L1.1.3, respectively. In contrast, we infer from the Pan-Asia *Mtb* dataset that 11% of both L1.1.1 and L1.2.2 isolates possess fixed mutations in *Rv0080* (Supp Table 1), and show that *Rv0080* attains the 8th highest NS mutation density for L1.1.1 and the 55th for L1.2.2 (although this ranking for L1.2.2 is based off only two NS mutations). Consistent with the inferences from our manuscript, there's no apparent signal of selection for *Rv0080* amongst isolates from L1.2.1, with *Rv0080* ranking 405th for this sublineage. We hope that this reanalysis of the dataset of Liu et al. (2022) has demonstrated that *Rv0080* is, in fact, under positive selection amongst the lineage 1 isolates it contains, and highlighted some key considerations when contrasting the results of selective screens which use unfixed versus fixed mutation data.

Insight into *zur* evolution from the dataset of Liu et al. (2022)
Finally, as we now note in the text (**Results lines 752-756**), our analysis of the Liu et al. (2022) dataset sheds light on aspects of *zur* evolution not recognised by the authors. While appearing in the top 30 genes in Figure 1b of Liu et al. (2022), our lineage-specific screen revealed that *zur* ranks outside the top 100 genes when screening lineage 2 isolates, and outside the top 80 when screening lineage 4. We therefore demonstrate that *zur* evolution is driven overwhelmingly by lineage 1 isolates, as we infer from the Vietnamese dataset. We thought this finding was relevant given the reviewer's premise that the appearance of *zur* in Figure 1b of Liu et al. (2022) compromises the novelty of our work on this gene. Although appearing in this figure, *zur* is not mentioned in the main text of their article, nor functionally characterised to the extent of our paper (see Comment 4).

8. Lines 563-566 in Results, 765-767 in Discussion, and 939-940 in Methods. Although the fsq gene is "intact" in M. canettii strain 140070008, it is frameshifted similarly to M. tuberculosis H37Rv in other publicly available genomes of M. canettii strains, such as 140010059 and 140060008, indicating that the frameshift-inducing mutation predates the emergence of M. tuberculosis from M. canettii.

We thank the reviewer for their scrutiny of patterns of *Rv3129* variation amongst *M. canettii* strains. We have edited the relevant section of the Results to clarify our inferences regarding the timing of this *Rv3129* frameshift during the emergence of *Mtb* (**Results lines 641-647**). We have also performed additional bioinformatic analysis using the *M. canettii* genomes supplied by Supply et al. (2013), which yielded a surprising finding suggestive of ongoing selection for a loss of *Rv3129* function amongst *M. canettii* strains (**Results lines 647-652 and Methods lines 1271-1278**).

Clarifying the timing of the *Rv3129* frameshift in *Mtb* evolution

The analysis of Supply et al. (2013) shows *M. canettii* to be comprised of a number of genetically diverse strains. The SNP distance matrix and neighbour joining tree of Supply et al. (2013) (Figures 1c and 3, respectively) show that these strains display varying genetic distances from *Mtb*, and that *Mtb* is genetically closer to some strains of *M. canettii* than these *M. canettii* strains are to one another. Supply et al. (2013) also show that strains of *M. canettii* show variation in their levels of virulence, with the most distantly related to *Mtb* (strain K) being the least virulent: a finding also supported by Malaga et al. (2023).

As we now clarify, our observation regarding the evolution of *Rv3129* is that the two *M. canettii* strains which are most genetically similar to *Mtb* (strains A – 140010059 and D – 140060008), are the two which possess the same frameshift-inducing indel in the gene. Although we can't directly link this genetic change to an increase in virulence or survival capacity, we can infer that this change took place relatively recently during the evolutionary timescale of the emergence of *Mtb* from *M. canettii*. We show in Supp Figure 21 the neighbour joining tree of Supply et al. (2013), augmented with a symbol marking strains possessing this specific indel (red symbols).

Evidence of selection for a loss of *Rv3129* function amongst *M. canettii* strains

The interest in our *Rv3129*-related finding shown by the reviewer inspired a more thorough investigation of *Rv3129* variation within the five high quality *M. canettii* assemblies published by Supply et al. (2013). This analysis consisted of conducting a BLAST search of the 'intact' *Rv3129* amino acid sequence against each *M. canettii* assembly, and translating the corresponding hit into an amino acid sequence (**Methods lines 1271-1278**).

In addition to finding the same frameshift-inducing indel in strains A and D, we also found two other frameshift mutations in the *Rv3129* sequence. Strain J displayed a 230bp deletion within the *Rv3129* coding sequence (Supp Figure 20), and strain K, despite registering a full-length match for the intact *Rv3129* sequence, possessed a frameshift mutation within the last several residues, leading to an elongated transcript. Of the five high quality *M. canettii* assemblies, we therefore document three independent pseudogenisation events, and an intact version of *Rv3129* in only one strain (strain L) (Supp Figures 20 & 21).

By synthesising data presented by Supply et al. (2013), we can deduce that this pattern of variation marks *Rv3129* as a gene of interest. Of the 81 'interrupted coding sequences' examined by Supply et al. (2013), only four are pseudogenised in *Mtb* and intact in *M. canettii*, and an additional two are pseudogenised in all five *M. canettii* strains but intact in the *M. marinum* outgroup (see Supp Table 5 of Supply et al. 2013). *Rv3129* appears to fall into a unique category of genes not explored by Supply et al. (2013), consisting of genes

pseudogenised in *Mtb* and a subset of *M. canettii* strains (although *Rv3129* was not recognised as an interrupted coding sequence in their study). Combined with the evidence of selection for a loss of function described above and its close relationship to *Rv0080* marks *Rv3129* as a gene of strong interest for subsequent analysis.

References (for responses to Reviewer 1)

Chiner-Oms, A., Sánchez-Busó, L., Jukka Corander, Sébastien Gagneux, Harris, S. R., Young, D. B., González-Candelas, F., & Comas, I. (2019). Genomic determinants of speciation and spread of the Mycobacterium tuberculosis complex. Science Advances, 5(6). https://doi.org/10.1126/sciadv.aaw3307

Chiner-Oms, A., Lopez, M. A., Moreno-Molina, M., Furió, V., & Comas, I. (2022). Gene evolutionary trajectories in Mycobacterium tuberculosis reveal temporal signs of selection. Proceedings of the National Academy of Sciences of the United States of America, 119(17). https://doi.org/10.1073/pnas.2113600119

Culviner, P. H., Frey, A. M., Liu, Q., Ha, D. T. M., Thai, P. V. K., Thu, D. D. A., Quang, N. L., Calderon, R., Lecca, L., Caws, M., Dunstan, S. J., Murray, M. B., Thuong, N. T. T., & Fortune, S. M. (2025). Evolution of Mycobacterium tuberculosis transcription regulation is associated with increased transmission and drug resistance. Cell. https://doi.org/10.1016/j.cell.2025.09.005

Liu, Q., Zhu, J., Dulberger, C. L., Stanley, S., Wilson, S., Chung, E. S., Wang, X., Culviner, P., Liu, Y. J., Hicks, N. D., Babunovic, G. H., Giffen, S. R., Aldridge, B. B., Garner, E. C., Rubin, E. J., Chao, M. C., & Fortune, S. M. (2022). Tuberculosis treatment failure associated with evolution of antibiotic resilience. Science, 378(6624), 1111–1118. https://doi.org/10.1126/science.abq2787

Maciag, A., Dainese, E., G. Marcela Rodriguez, Milano, A., Provvedi, R., Maria Rosalia Pasca, Smith, I., Giorgio Palù, Riccardi, G., & Manganelli, R. (2007). Global analysis of the Mycobacterium tuberculosis Zur (FurB) regulon. 189(3), 730–740. https://doi.org/10.1128/jb.01190-06

Malaga, W., Payros, D., Meunier, E., Frigui, W., Sayes, F., Pawlik, A., Orgeur, M., Berrone, C., Moreau, F., Mazères, S., Gonzalo-Asensio, J., Rengel, D., Martin, C., Astarie-Dequeker, C., Mourey, L., Brosch, R., & Guilhot, C. (2023). Natural mutations in the sensor kinase of the PhoPR two-component regulatory system modulate virulence of ancestor-like tuberculosis bacilli. PLoS Pathogens, 19(7), e1011437. https://doi.org/10.1371/journal.ppat.1011437

Marin, M., Vargas, R., Harris, M., Jeffrey, B., Epperson, L. E., Durbin, D., Strong, M., Salfinger, M., Iqbal, Z., Akhundova, I., Vashakidze, S., Crudu, V., Rosenthal, A., & Farhat, M. R. (2022). Benchmarking the empirical accuracy of short-read sequencing across the M. tuberculosis genome. Bioinformatics, 38(7), 1781–1787. https://doi.org/10.1093/bioinformatics/btac023

Sonnenkalb, L., Carter, J. J., Spitaleri, A., Iqbal, Z., Hunt, M., Malone, K. M., Utpatel, C., Cirillo, D. M., Rodrigues, C., Nilgiriwala, K. S., Fowler, P. W., Merker, M., Niemann, S., Barilar, I., Battaglia, S., Borroni, E., Brandao, A. P., Brankin, A., Cabibbe, A. M., & Carter, J. (2023). Bedaquiline and clofazimine resistance in Mycobacterium tuberculosis: an in-vitro and in-silico data analysis. The Lancet Microbe, 4(5), e358–e368. https://doi.org/10.1016/S2666-5247(23)00002-2

Supply, P., Marceau, M., Mangenot, S., Roche, D., Rouanet, C., Khanna, V., Majlessi, L., Criscuolo, A., Tap, J., Pawlik, A., Fiette, L., Orgeur, M., Fabre, M., Parmentier, C., Frigui, W., Simeone, R., Boritsch, E. C., Debrie, A.-S., Willery, E., & Walker, D. (2013). Genomic analysis of smooth tubercle bacilli provides insights into ancestry and pathoadaptation of Mycobacterium tuberculosis. Nature Genetics, 45(2), 172–179. https://doi.org/10.1038/ng.2517

Vargas, R., Luna, M. J., Freschi, L., Marin, M., Froom, R., Murphy, K. C., Campbell, E. A., Ioerger, T. R., Sassetti, C. M., & Farhat, M. R. (2023). Phase variation as a major mechanism of adaptation in Mycobacterium tuberculosis complex. Proceedings of the National Academy of Sciences, 120(28). https://doi.org/10.1073/pnas.2301394120

Wilson, D. J., Crook, D. W., Peto, T. E. A., Walker, A. S., Hoosdally, S. J., Cruz, A. L. G., Carter, J., Grazian, C., Earle, S. G., Kouchaki, S., Lachapelle, A., Yang, Y., Clifton, D. A., Fowler, P. W., Iqbal, Z., Hunt, M., Knaggs, J., Smith, E. G., Rathod, P., … van Soolingen, D. (2020). GenomegaMap: Within-Species Genome-Wide dN/dS Estimation from over 10,000 Genomes. Molecular Biology and Evolution, 37(8), 2450–2460. https://doi.org/10.1093/molbev/msaa069

Reviewer #2 (Remarks to the Author):
The manuscript by Silcocks et al. provides a compelling analysis of ongoing positive selection in the Mycobacterium tuberculosis complex (MTBC), leveraging a large dataset of Thai genomes to identify three loci under strong diversifying selection: a dormancy gene (Rv0080), a zinc homeostasis gene (zur), and a virulence-associated gene (ESX1). Focusing on Rv0080, the authors robustly demonstrate repeated reductive evolution of this dormancy survival regulon across the MTBC phylogeny, particularly in the ancestral L1 lineage, and elucidate mutation-driven changes in molecular secondary structure, dimer stability, and hypoxia survival, validated through ΔRv0080 mutant and wild-type M. smegmatis comparisons, as well as L1 MTBC strain analyses.

I have several critiques. First, the use of a 'flat' dN/dS ratio to identify candidate genes is a limitation. More sophisticated models, such as those implemented in PAML, which partition proteins into fractions experiencing negative, neutral, or positive selection, better reflect complex biological dynamics. The current approach likely captures only extreme cases of positive selection, potentially overlooking genes under localized selection pressure, such as virulence factors with dN/dS ratios near 1 despite positive selection. Incorporating such models, though computationally intensive, would strengthen the findings.

We thank the reviewer for their suggestion of applying ‘site’ models of the PAML software package to identify genes which are conserved (or evolving neutrally), on average, but possess a subset of sites under positive selection. We’ve applied the CODEML algorithm of the PAML software package to explore this phenomenon, and detail our results in **lines 206-229**, and our methods in **lines 1243-1252**.

As mentioned, we used this approach to calculate both the proportion of sites in each gene experiencing, purifying selection (dN/dS < 1), positive selection (dN/dS > 1) and neutral evolution (dN/dS = 1), and the proportion of sites with posterior probabilities of selection (dN/dS > 1) above various thresholds. Following the approach of Wilson et al. (2020), we chose to summarise our results in terms of these posterior values per site, as this approach gives us a more quantitative measure of the degree of selection per site, and allows us to identify specific sites for prioritisation.

We visualised the CODEML output by plotting the dN/dS value per gene against the proportion of sites in that gene with a posterior probability of selection of 0.9 or greater (Supp Figure 2). The utility of this approach for identifying functionally relevant genes is demonstrated by its retrieval of several known drug resistance genes, including *prpD*, *Rv1129c* and *dnaA*, which are globally conserved (i.e. low overall dN/dS ratio), yet contain a relatively high fraction of sites displaying positive selection. In addition to these drug resistance genes, we also identified the novel gene candidate *leuA*, which displays a similar pattern, without a known link to antibiotic resistance.

Ultimately, we saw prioritising genes identified in this screen (such as *leuA*), to be beyond the scope of this study, which has already identified and analysed two extreme targets of positive selection, which went unrecognised in prior studies (*Rv0080* and *zur*). We’re hopeful that genes identified in this screen may be the subject of future research, or that the

CODEML-based pipeline we've used may inspire the approach of future studies of selection in *Mtb*.

Another brief observation was that it was reassuring to see *zur* and *Rv0080* both attained prominent positions in this scatterplot (Supp Figure 2), which provides an additional line of evidence for the key inferences of this paper.

Second, the manuscript lacks discussion of the temporal dynamics and genetic diversity within MTBC lineages. Older lineages, like L1, naturally accumulate more non-synonymous mutations than younger ones, such as L2, which likely emerged during domestication in China. Recent population bottlenecks in modern lineages could also accelerate fixation of Rv0080 mutations (e.g., frameshifts or deficient mutants), a point that warrants mention.

We thank the reviewer for their insightful comments on the temporal dynamics of dN/dS and the possibility of drift explaining the presence of some of the *Rv0080* mutations observed. We have now completed an analysis of how the dN/dS ratio varies temporally across branches of our Vietnamese phylogeny, and demonstrate that the magnitude of variation is minor, and unlikely to influence the main conclusions of our study. We have also now expanded our Discussion to consider the possibility that genetic drift is responsible for producing the patterns of *Rv0080* variation observed, and the lines of evidence which render this hypothesis unlikely (**Discussion lines 897-901**).

Exploration of the temporal dynamics of dN/dS
We agree with the reviewer that dN/dS is a metric which varies depending on the timescale of measurement, with progressively lower dN/dS values estimated through comparisons of species separated by a longer evolutionary timescale (Rocha et al. 2006). To explore this phenomenon within an *Mtb* population and ensure it doesn't influence the key conclusions of our paper, we have investigated the relationship between the dN/dS ratio of each branch of the phylogeny, and the distance of that branch from the root of the tree in our Vietnamese dataset.

To perform this analysis, we calculated a genome-wide dN/dS ratio for each branch of the phylogeny which accumulated at least one synonymous mutation for lineages 1 and 2. We visualise the dN/dS per branch as a heat-scale in Figure R2, and generated a scatterplot below showing the relationship between dN/dS per branch and the distance of the midpoint of that branch from the root of the tree. We found no correlation between dN/dS and distance from the root of the tree within the lineage 1 phylogeny (Pearson's correlation test, $p=0.308$), and a marginally significant positive correlation within the lineage 2 phylogeny ($p=0.0466$, correlation coefficient = 0.0432).

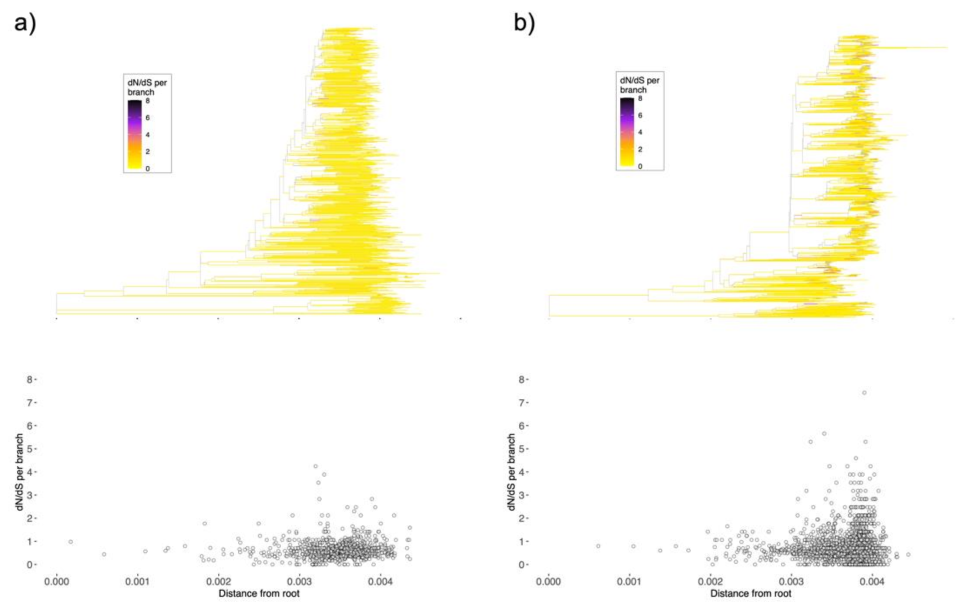

Figure R2) Phylogenies of the lineage 1 (panel a; upper) and lineage 2 (panel b; upper) isolates from our Vietnamese dataset, with branches marked according to their branch-specific dN/dS ratio. Also shown are scatterplots displaying the distribution of dN/dS values with distance from the root of each tree (lower panels). Each point corresponds to a branch in the above plot.

Consistent with the study of Namouchi et al. (2012), we found terminal branches of the phylogeny to display higher dN/dS values, on average, compared to internal branches, for both lineages 1 and 2 (Mann Whitney U test, $p=2.03 \times 10^{-4}$ for L1 and $p=2.17 \times 10^{-12}$ for L2) (Figure R3). When filtering out terminal branches, the weak correlation between dN/dS per branch and distance from the root for lineage 2 was no longer significant ($p=0.196$). We conclude, as do Namouchi et al. (2012), that these excess non-synonymous mutations are likely in the process of being purged by selection. In conclusions, we've found there to be little temporal variation in the dN/dS ratio across branches of the *Mtb* phylogeny, and have uncovered no apparent sources of bias which would influence our inferences regarding selection in *Rv0080* or other key genes.

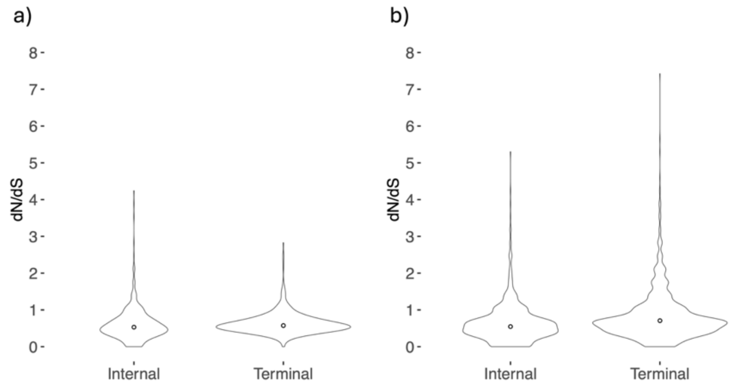

Figure R3) Violin plots showing the distributions of branch-specific dN/dS ratios on internal vs terminal branches of the phylogeny for lineage 1 (panel a) and lineage 2 (panel b).

Drift alone is unlikely to explain the patterns of *Rv0080* variation observed
We have also amended our Discussion to better contextualise the likelihood of genetic drift explaining the patterns of variation observed for *Rv0080* (**Discussion lines 897-901**). Strictly speaking, even if *Rv0080* wasn't under selection, it would still be possible for it to accumulate one or more mutations across the *Mtb* complex phylogeny by chance. Observing five mutations, however, is more than a chance occurrence, and our mutation randomisation analysis places the probability of observing five or more mutations in a gene of *Rv0080*'s size at one in 25,000 ($p=4x10^{-5}$). Considered alongside the functional relevance of these five mutations (two nonsense, two FAD-binding or adjacent, one highly destabilising), and the signal of selection observed for *Rv0080* in the remaining two human-adapted lineages (L1 and L9), the possibility of drift explaining the pattern of variation observed is further reduced. We have updated our Discussion to better contextualise these lines of evidence.

Third, the discussion of the non-replicative state during dormancy (line 234) overlooks ongoing debates, with studies from Sarah Fortune and others challenging this paradigm, which deserves acknowledgment to contextualize the findings.

We thank the reviewer for highlighting some very relevant literature, emphasising the lack of understanding of the dormant phase of *M. tuberculosis*. We have amended our Discussion to mention the Fortune lab's study which inferred surprisingly high *Mtb* substitution rates during dormancy in non-human-primates (Ford et al. 2011). We have also mentioned a study which reaches a similar conclusion in humans (Lillebaek et al. 2016), and two others which infer much lower substitution rates during dormancy (Colangeli et al. 2014 and 2020). Finally, we have also mentioned a prior study of non-human-primates which documents a wide range of bacterial burdens and histopathology's in cases classified as 'latent' (Lin et al. 2009). We have concluded that a more comprehensive understanding of the dynamics of *Mtb* dormancy and physiological challenges faced by the bacteria may help generate new hypotheses regarding the function of *Rv0080*. Each of the above amendments have been applied to the Discussion, between **lines 920-930**.

Finally, Figure S1's gene labels in panels a and b are nearly illegible due to small font size, hindering interpretation.

We thank the reviewer for pointing out the issue with the image quality of this figure, and have now substituted it with a high resolution version.

This manuscript's key contribution lies in highlighting how MTBC's population genetic structure shapes distinct adaptive landscapes across lineages and sublineages, driven by epistatic interactions. These insights underscore the need for precision medicine in tuberculosis treatment, offering a path toward tailored therapies and a deeper understanding of global TB dynamics. I recommend acceptance for publication in Nature Communications after addressing these concerns to enhance methodological rigor and clarity.

We thank the reviewer for this endorsement of our work, and hope the amendments we've made to our manuscript have adequately addressed their concerns.

References (for responses to Reviewer 2)

Colangeli, R., Arcus, V. L., Cursons, R. T., Ruthe, A., Karalus, N., Coley, K., Manning, S. D., Kim, S., Marchiano, E., & Alland, D. (2014). Whole Genome Sequencing of Mycobacterium tuberculosis Reveals Slow Growth and Low Mutation Rates during Latent Infections in Humans. PLoS ONE, 9(3), e91024. https://doi.org/10.1371/journal.pone.0091024

Colangeli, R., Gupta, A., Vinhas, S. A., Chippada Venkata, U. D., Kim, S., Grady, C., Jones-López, E. C., Soteropoulos, P., Palaci, M., Marques-Rodrigues, P., Salgame, P., Ellner, J. J., Dietze, R., & Alland, D. (2020). Mycobacterium tuberculosis progresses through two phases of latent infection in humans. Nature Communications, 11(1). https://doi.org/10.1038/s41467-020-18699-9

Ford, C. B., Lin, P. L., Chase, M. R., Shah, R. R., Iartchouk, O., Galagan, J., Mohaideen, N., Ioerger, T. R., Sacchettini, J. C., Lipsitch, M., Flynn, J. L., & Fortune, S. M. (2011). Use of whole genome sequencing to estimate the mutation rate of Mycobacterium tuberculosis during latent infection. Nature Genetics, 43(5), 482–486. https://doi.org/10.1038/ng.811

Lillebaek, T., Norman, A., Rasmussen, E. M., Marvig, R. L., Folkvardsen, D. B., Andersen, Å. B., & Jelsbak, L. (2016). Substantial molecular evolution and mutation rates in prolonged latent Mycobacterium tuberculosis infection in humans. International Journal of Medical Microbiology, 306(7), 580–585. https://doi.org/10.1016/j.ijmm.2016.05.017

Lin, P. L., Rodgers, M., Smith, L., Bigbee, M., Myers, A., Bigbee, C., Chiosea, I., Capuano, S. V., Fuhrman, C., Klein, E., & Flynn, J. L. (2009). Quantitative Comparison of Active and Latent Tuberculosis in the Cynomolgus Macaque Model. Infection and Immunity, 77(10), 4631–4642. https://doi.org/10.1128/iai.00592-09

Namouchi, A., Didelot, X., Schöck, U., Gicquel, B., & Rocha, E. P. C. (2012). After the bottleneck: Genome-wide diversification of the Mycobacterium tuberculosis complex by mutation, recombination, and natural selection. Genome Research, 22(4), 721–734. https://doi.org/10.1101/gr.129544.111

Rocha, E. P. C., Smith, J. M., Hurst, L. D., Holden, M. T. G., Cooper, J. E., Smith, N. H., & Feil, E. J. (2006). Comparisons of dN/dS are time dependent for closely related bacterial genomes. Journal of Theoretical Biology, 239(2), 226–235. https://doi.org/10.1016/j.jtbi.2005.08.037

Wilson, D. J., Crook, D. W., Peto, T. E. A., Walker, A. S., Hoosdally, S. J., Cruz, A. L. G., Carter, J., Grazian, C., Earle, S. G., Kouchaki, S., Lachapelle, A., Yang, Y., Clifton, D. A., Fowler, P. W., Iqbal, Z., Hunt, M., Knaggs, J., Smith, E. G., Rathod, P., … van Soolingen, D. (2020). GenomegaMap: Within-Species Genome-Wide dN/dS Estimation from over 10,000 Genomes. Molecular Biology and Evolution, 37(8), 2450–2460. https://doi.org/10.1093/molbev/msaa069

REVIEWER COMMENTS

Reviewer #1 (Remarks to the Author):
I appreciate that many of my comments have been addressed, substantially improving the manuscript. However, I continue to have significant concerns regarding the reproducibility and interpretation of some previous or new results from hypoxia survival experiments, which constitute a major experimental support for the conclusion on reductive evolution of the dormancy regulon. I also have additional comments that were not included in my initial review, given the number and nature of concerns that were raised at that stage.

We thank the reviewer again for their careful review of our manuscript. Once again, we believe it has provided us the opportunity to substantially improve the quality of our paper and clarify important details.

In response to Comment 1 and the 'Additional Comments' below, we have edited the *M. smegmatis* hypoxia survival section to only present data from strains which produce highly consistent survival advantages between replicates, and edited the Results and Discussion to remain speculative about the functional consequence of lineage-defining point mutations in *Rv0080* (i.e. V60G and V31G). While expressing uncertainty surrounding these mutations, we now clarify in greater detail here and in the manuscript how available data supports the notion that a loss of *Rv0080* function played an important role in MTBC evolution, by placing a greater emphasis on data (both genetic and functional) pertaining to frameshift mutations.

We have also edited our manuscript to clarify our observations regarding the lineage 2 Thai isolates with the *Rv0080* frameshift mutation, and have selected alternative genes to *phoR* as examples of those experiencing selection for a subtle alteration of function (as opposed to a complete loss). Finally, we have completed additional bioinformatic analysis to confirm the presence of the V60G substitution in *Rv0080* across all modern lineage strains from our datasets, confirmed the presence of V31G in all L6 strains, and performed an investigation of the functional consequences of mutations in *Rv0042c*. Detailed responses to each comment are given below.

We have marked all edits to the Main Text with yellow highlighting, and have also attached a version with tracked changes, in order to show passages of text we've deleted. Please note that the line numbers we quote below correspond to the former of these two documents, and also correspond to the latter document when tracked changes are disabled.

1. Lines 618-627 in Results and 907-909 in Discussion. The conclusion that the mutations V60G and V31G that evolved along deep, lineage defining branches of the Mtb phylogeny result in enhanced survival phenotypes in M. smegmatis is not robustly supported, when the results of the replicate datasets in Figure 4e and Supplementary Figure 19 are examined closely.

In Supplementary Figure 19, V60G appears to display a substantial survival advantage over both the wild type and ancestral alleles from day 5 to 10. In contrast, in Figure 4e, this survival advantage is, at best, limited to a ~1-log difference on day 7 only. The mean

difference on day 14 is only ~0.5 log and likely within overlapping standard deviations (which are not shown).

Conversely, while the survival advantage of V31G over the wild type and ancestral alleles appears substantially higher (~1-log) on both day 7, 10 and 14 in Figure 4e, the supplemental data show a 1-log difference only on a single time point (day 6). In this figure, a substantial difference at day 7 is seen only with the ancestral allele, but these ancestral datapoints fall even below the wild type values and are rather suggestive of outliers than a reproducible biological effect. There is no difference at all between the V31G, wild type and ancestral alleles at day 10.

We thank the reviewer for their careful scrutiny of the hypoxia survival readings for our strains carrying the lineage-defining *Rv0080* alleles V31G and V60G. While these strains do display, on average, a survival advantage relative to the ancestral allele, we agree that this advantage is not strongly consistent across replicates of the experiment (as the remainder of alleles are). As per the suggestion they provide in their 'Additional Comments', we have restricted the scope of this experiment to focus on the strains producing strongly consistent survival phenotypes between replicates: the wild type, Δ*MSMEG_5136*, the ancestral allele, and the three alleles selected from our Vietnamese dataset: V49G, L121P and the frameshift allele (**Figure 4d**; **Results lines 593-611**).

In line with the above, we have updated our Discussion to highlight our uncertainty regarding the specific phenotypic consequences of the V60G and V31G mutations, and the need to investigate these further, specifically in an *Mtb* model (**lines 901-902**; **lines 906-908**; **lines 913-916**). While remaining tentative regarding these point mutations, we now justify in this section how available genetic and functional data supports the notion that *Rv0080* played an important role in the evolution of the *Mtb* complex (**lines 893-908**). We do this by placing greater emphasis on the role of frameshift mutations, which we show are significantly enriched in *Rv0080* across the lineage-defining branches of the *Mtb* phylogeny ($p=1.0x10^{-3}$; **lines 480-483**), and which we show to result in enhanced hypoxia survival (**Figure 4d**) (albeit in an *M. smegmatis* model). These edits and other relevant details are outlined in greater detail in our response to the 'Additional Comments' below.

2. Lines 583-598 in Results. As indicated in my previous comments, the mean differences in survival under hypoxia between the tested non-isogenic Rv0080 "mutant" and "wild-type" M. tuberculosis isolates reported in Supplementary Figure 18 are small, mostly below 1-log CFU. Moreover, overlapping datapoints have to be acknowledged between the wild type and both mutants for day 18, and the wild type and the frameshift mutant for day 24, and not only for day 42, where the wild type appears to survive even better than the mutants. Even if presented as a tentative interpretation, these experimental data do not provide convincing support for the hypothesis that loss or alteration of Rv0080 function confers a survival advantage in a native (albeit non-isogenic) M. tuberculosis background.

We thank the reviewer for their additional scrutiny of our hypoxia survival experiment using clinical *Mtb* strains. During the last round of revisions, we made edits to this section to express our tentativeness surrounding the conclusions we can draw from this experiment

when considered in isolation. We understand if the preference of the reviewer is to remove this *Mtb* data, and have edited the relevant section of the Results (**lines 572-611**) in the revised manuscript to do so. As per their 'Additional Comments', we have also edited the Discussion (**lines 901-902**; **lines 906-908**; **lines 913-916**) to highlight the objective of obtaining data for isogenic *Mtb* strains with *Rv0080* mutations as an avenue of future research. While we don't have conclusive data for the *Mtb* strains tested, we note that our *M. smegmatis* experiment does include the two *Rv0080* alleles in question (L121P and frameshift), and demonstrates the enhanced hypoxia survival associated with these two mutations on an isogenic background.

3. Lines 491-501 in Results. As the text stands, the speculation that the lack of selection for Rv0080 in lineages 2, 4 and 6 can be explained via the loss-of function mutations acquired on their lineage-defining branches may appear contradictory with the presence of an additionally acquired premature stop codon in Rv0080 in a large L2 clade of 22 Thai isolates from the Pan-Asia dataset. This might on the contrary be interpreted as evidence for still ongoing selection for loss-of-function in L2. A clearer possible explanation could be that the presence of this non-sense mutation might reflect further pseudogenization of Rv0080 due to drift, after the acquisition of the LoF mutation at lineage-defining branch level. However, see the comments above on V60G hypoxia survival phenotype in M. smegmatis.

We thank the reviewer for highlighting some alternate interpretations of the presence of an *Rv0080* frameshift mutation amongst a clade of 22 Thai isolates, and have updated this section of the Results (**lines 503-514**) to better articulate the inferences we draw from it. To summarise: the presence of this clade suggests that *Rv0080* is non-essential for the survival and virulence of modern lineages, and that complete loss of function mutants do not experience strong purifying selection on a modern lineage background. Below we also outline data illustrating that the presence of this clade alone is not sufficient evidence to suggest modern lineages undergo ongoing selection for a full and unambiguous loss of function.

This data implies that *Rv0080* is non-essential on a lineage 2 background
The significance of this cluster of 22 strains from lineage 2 carrying the same *Rv0080* frameshift mutation indicates that a strain had at some point evolved this mutation, survived, and subsequently transmitted to multiple new hosts. We can therefore interpret this as evidence that *Rv0080* is not essential for the survival or transmission of strains from lineage 2. We would perhaps be more tentative in this inference if we were to observe frameshift mutations in one or two solitary L2 isolates (which therefore haven't displayed the capacity to transmit after evolving the *Rv0080* mutation), as these may be 'unfit' strains soon to be purged by the action of purifying selection.

This data alone isn't sufficient evidence for ongoing selection for a loss of function in L2
A plausible hypothesis mentioned by the reviewer is that modern lineages isolates, although carrying the V60G mutation, may still experience selection for mutations resulting in a complete ablation of *Rv0080* function (i.e. a frameshift mutations). Note that the hypothesis assumes that strains with the V60G mutation still retain some degree of *Rv0080* function, and do not lose it entirely (as discussed by the reviewer in Comment 7).

Whilst this hypothesis is plausible, it is not strongly supported by our available genomic data, as evidenced by the low frequency of frameshift mutation evolution events amongst modern lineage isolates. For instance, while there are two independent frameshift-inducing mutation events in the L2 isolates from the Pan Asia dataset (**Supp Figure 13**; n=686 isolates), there is only one in the Vietnamese dataset (n=1,589). Similarly, none are observed amongst lineage 4 isolates in either dataset (n=247 in Pan Asia and n=271 in Vietnamese dataset), and amongst SNP data from the L2/L4 strains of Liu et al. (2022) (L2 n=13,650; L4 n=26,183), we don't observe the evolution of any mutations resulting in premature stop codons.

Confirming V60G results in a complete loss of function will require functional data
Finally, we believe the latter hypothesis mentioned by the reviewer (whereby this frameshift represents further pseudogenisation following the V60G mutation) is a likely one, yet one which will require functional data to prove definitively. As we mention in other comments, we have amended the Discussion (**lines 901-902**; **lines 913-916**) to highlight the need to establish the function effects of this V60G mutation.

4. Lines 494-497 in Results. It should be confirmed whether all L2 & L4 genomes from the Pan-Asia dataset and all 277 L6 genomes carry the V60G and V31G mutations, as these were inferred to be have been acquired at their respective lineage-defining-branch levels based on the LAA-Z dataset.

We thank the reviewer for their suggestion of screening the relevant genomic datasets to confirm that all modern lineage strains (L2 and L4), and lineage 6 strains we analyse carry their respective lineage-defining *Rv0080* substitutions (V60G for L2 and L4, and V31G for L6).

A note on our approach: we localised these mutations to the lineage defining branches of the *Mtb* complex phylogeny by screening the LAA-Z dataset, which contains representatives of the deepest known split in each *Mtb* lineage. Strains representing the deepest split within each lineage were selected after careful inspection of phylogenies published in prior large-scale *Mtb* genomic projects. It follows that there's a strong expectation that all members of a lineage will carry that lineage's lineage-defining mutations, regardless of what dataset they're inferred from (i.e. Vietnamese, Pan Asia, etc). There are, however, three unlikely (yet plausible) scenarios under which the above does not hold: when a back-mutation takes place, when a large genomic deletion of the locus and surrounding region occurs, or when the dataset analysed contains a strain(s) descending from a deeper, yet unidentified split in the *Mtb* complex phylogeny.

To account for these three possibilities, we screened the variant call files from our Vietnamese dataset and confirmed that all 1,589 lineage 2 isolates, in addition to all 271 lineage 4 isolates possessed the 'G' nucleotide (as opposed to the 'T' nucleotide) at position 89,200 in the H37Rv reference genome (which is responsible for the V60G amino acid substitution in *Rv0080*). We also screened the variant call file for the Pan Asia *Mtb* dataset and confirmed all 686 lineage 2 isolates and all 247 lineage 4 isolates also possessed the 'G' nucleotide at position 89,200. Finally, we screened variant calls for all 277 lineage 6 isolates included in our analysis, and confirmed that all possessed the 'G' nucleotide (as opposed to

the ‘T’ nucleotide) at position 89,113 in the H37Rv reference genome (which is responsible for the V31G amino acid substitution). To illustrate this visually, we plot below the phylogeny for the Vietnamese *Mtb* dataset with tip-points indicating the genotype (i.e. the nucleotide) for position 89,200 (**Figure R1**), and can plot additional phylogenies if requested by the reviewer. We have also now documented these findings in the Results section (**lines 497-500**), and agree that it adds important rigour to our analysis.

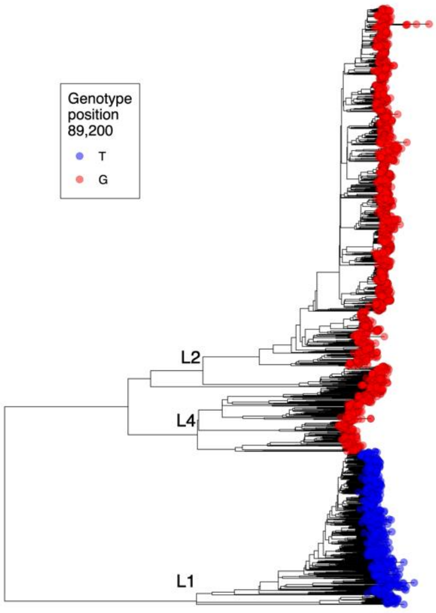

Figure R1) Phylogeny of Vietnamese *Mtb* isolates from lineages 1, 2 and 4, with the genotype of position 89,200 in the H37Rv reference genome indicated in tip-point colour. This mutation is responsible for the V60G amino acid substitution in *Rv0080*, with the ‘G’ nucleotide resulting in the Glycine (‘G’) amino acid and the ‘T’ nucleotide resulting in the Valine (‘V’) amino acid. All L1 isolates possess the ‘T’ nucleotide, and all L2 and L4 isolates possess the ‘G’ nucleotide.

5. Lines 503-514 in Results. As overexpression of the transcription factor Rv0042c is a main driver of Rv0079-Rv0080 expression, is there any indication that NS mutations in Rv0042c might be loss-of-function variants, in order to support the conclusion that extreme signal of L1-specific positive selection observed for Rv0042c may represent a mechanism of reducing or eliminating the expression of Rv0080?

We thank the reviewer for their suggestion of exploring the functional consequences of *Rv0042c* mutations amongst lineage 1 isolates, to test the hypothesis that a loss of function is a mechanism targeted by selection. Following the framework used for *Rv0080* and *zur*, we did this by assessing the distribution of *Rv0042c* mutations with respect to its orthologues, and the frequency of frameshift-inducing indels in the *Rv0042c* coding sequence (see **Results lines 527-532**).

To summarise, we find that 75.9% of *Rv0042c* mutations hit residues that are conserved in an alignment of its orthologues, which is higher than expected if mutations were distributed

randomly (chi squared test, p=0.012) (**Supp Figure 16**). In addition, we document a total of 6 lineage 1 strains which possess frameshift-inducing indels in the *Rv0042c* coding sequence. Collectively, we conclude this data to indicate that a loss of function is one of the mechanisms of *Rv0042c* evolution targeted by selection.

We close by noting that this finding is directionally consistent with the transcription factor overexpression data of Rustad et al. (2014), which indicates increased expression of *Rv0042c* to lead to an increase in *Rv0080* expression (**Supp Figure 14**). It follows that a loss of function of *Rv0042c* can be expected to decrease the expression of *Rv0080*, and produce an analogous biological effect to a reduction or elimination of *Rv0080* function. We have added this observation to our Results section (**lines 532-535**).

6. Lines 844-848 in Discussion. The fact that indels are never observed in phoR is fully expected, as the selective pressure acting on phoR is opposite compared to Rv0080: loss-of-function mutations are counter-selected because phoR is required for virulence in vivo.

We thank the reviewer for making this valid point about *phoR*: the example we gave of a gene under selection for a subtle alteration, rather than complete loss of function. Although *phoR* is annotated as a 'non-essential' gene for in vitro growth, it has been shown to be essential for virulence in animal infection models, which is relevant given the nature of this clinical dataset we analyse.

To address this, we have edited our Discussion to instead give examples of genes thought to be 'non-essential', which our screen showed to display high dN/dS ratios, yet few to no frameshift-inducing indels (**lines 832-838**). The genes we selected were *Rv1779c* and *Rv0380c*, which displayed dN/dS ratios in the Vietnamese dataset of 2.66 and 5.56 respectively, yet frameshift mutation counts of 2 and 0. We close this paragraph of the Discussion by noting that exploring the frequency of complete loss of function mutations in this way will hopefully serve as means of elucidating the change in function under selected for each gene in future genomic screens.

7. Lines 913-918 in Discussion. "…there doesn't seem to be an obvious phenotypic advantage for a frameshift mutation, as suggested by the hypoxia survival data (Figure 4d; Supp Figures 18 and 19), and lack of ongoing selection for subsequent mutations amongst L2 and L4 strains. We speculate that, like zur, there could be a fitness trade-off, whereby a partly functional version of Rv0080 could be beneficial during some stages of infection."

This appears as an overinterpretation, as it is unknown whether the substitution mutations tested for hypoxia survival do not result in complete loss-of-function of Rv0080 too, as is expectedly the case with the gene deletion or frameshift mutations.

We thank the reviewer for making a valid point regarding our interpretation of the consequences of the V60G mutation on *Rv0080* function, and have reworded this section of the Discussion to address this (**lines 913-921**).

It is tempting to speculate that some of the point mutations observed in *Rv0080* such as V60G and V31G lead to less extreme alterations in *Rv0080* function relative to the complete loss of function induced by a frameshift mutation. The data we observed for *zur*, whereby certain point mutations lead to different, yet consistent levels of upregulation of *zur* regulon genes (**Supp Figure 25**) may provide some grounds on which to formulate this hypothesis, however we stress that this is speculative, and will require functional data to confirm. In this part of our Discussion, we also now highlight the related research question of whether retaining some degree of *Rv0080* function is beneficial during certain stages of infection.

8. Lines 114-124 in Introduction. "To help explain the differences in phenotypic manifestations between lineages, we recently proposed patterns of East Asian Mtb diversity to reflect the 'Two Layer' admixture model (Silcocks & Dunstan, 2023). Under this model, … Our Neolithic Mtb origin model provides a theoretical explanation for long-standing observations that 'evolutionarily ancient' lineages (lineages 1, 5, 6, 7, 8, 9 and 10) display lower levels of virulence and are more likely to result in latent infection than their 'modern' counterparts".

Actually, it is a paleolithic model that is strongly advocated for by Silcocks et al., as illustrated by the following sentence in their discussion: "We wish to note that our model of historic human and Mtb co-expansion is one which infers a Paleolithic origin of contemporary Mtb variation."

We thank the reviewer for highlighting this inconsistency with the Discussion section of our previous paper, and have updated the relevant passage of text in our Introduction for precision (**lines 119-120**).

To clarify, the main focus of this previous paper was to outline evidence illustrating the parallel expansion of human and *Mtb* lineages across East Asia, predominantly during the agricultural expansion of the Neolithic period. Our model does imply the origin of the association between humans and *Mtb* began prior to the Neolithic, however, so is more correctly described as a "Paleolithic origin model". We have updated the text to read "proposing a Paleolithic origin for the association of humans and *Mtb* and coexpansion during the Neolithic", as we believe this better describes the full scope of the model we proposed.

Reviewer #2 (Remarks to the Author):
I have reviewed the revised manuscript and the authors' point-by-point responses. The changes implemented fully address my previous comments on selection issues, as well as those of the second reviewer, resulting in a improved paper with enhanced clarity, rigor, and completeness.
I recommend acceptance of the manuscript in its current form.

We thank Reviewer 2 for their contribution to the review of this manuscript, and are pleased to hear we adequately addressed their concerns.

Additional Comments from Reviewer #1:
As confirmed with an expert biostatistician, rigorous statistical testing of the survival differences between the M. smegmatis strains carrying V60G and V31G and the WT and ancestral alleles would require pooling the data from both experiments, following normalization to D0.

However, the lack of reproducibility of the survival phenotypes of V60G and V31G relative to their comparator strains across the two experiments indicates that no consistent statistical support could be obtained.

Therefore, I do not think that any claim can be made regarding experimental support for the hypothesis of loss-of-function or reductive evolution of Rv0080 across all deep branches (other than L1) of the MTBC. The authors should limit themselves to a speculative interpretation, extrapolated from the frameshift mutations observed in the lineage-defining branches of L5 and L10.

A definitive experimental demonstration for all alleles could only be achieved in the M. tuberculosis genetic background. In principle, this could be done by introducing the available V60G, V31G, WT and ancestral constructs (as well as the Viet L1 alleles) into their available M. tuberculosis strain carrying a frameshift mutation in Rv0080. However, this would require several months of additional work and could perhaps be mentioned as a perspective for future work.

We thank the reviewer for these additional comments and recommendations regarding our hypoxia survival data. As we outline in our response to Comment 1, we have restricted the scope of our hypoxia experiment to present data from strains which produced highly reproducible CFU values between replicates (therefore including all but the V60G and V31G alleles) (**Figure 4**; **Results lines 572-611**). In line with these edits, we have also modified our Discussion to account for the fact we don't have direct functional data for lineage-defining point mutations like V60G and V31G, and have highlighted the need to generate this in future studies (**lines 901-902**; **lines 906-908**; **lines 913-916**), ideally in an *Mtb* model.

Despite lacking data on these specific point mutations, we have applied edits to emphasise the conclusions we can draw regarding the evolution of *Rv0080* across the deep evolutionary history of the *Mtb* complex, by placing greater emphasis on our data pertaining to frameshift mutations (see **Discussion lines 893-908**). The inclusion of a frameshift allele in our hypoxia assay (**Figure 4d**) means we have functional evidence that it results in enhanced hypoxia survival relative to the ancestral allele (albeit in an *M. smegmatis* model). We also find by repeating our permutation test (**Results lines 480-483**), that *Rv0080* displays a strongly significant enrichment of frameshift-inducing indels across the lineage-defining branches of the *Mtb* phylogeny ($p=1.0x10^{-3}$), being the only gene in the 100-200 amino acid range to accumulate 2 or more. Considering these two lines of evidence (the strong implausibility of the number of frameshifts observed and their proven functional consequences), we believe it reasonable to conclude that the frameshifts fixed in lineages 5 and 10 evolved to confer enhanced hypoxia survival for these lineages, and thus that *Rv0080* underwent selection for a loss of function during the early stages of MTBC evolution. We believe we have framed

these inferences with appropriate cautiousness, and highlight the need to obtain additional functional data from an *Mtb* model in future studies.

Despite lacking functional data for specific point mutations (i.e. V60G, V31G), we also believe that our genetic data alone implies that selection on *Rv0080* has acted across the deep evolutionary history of the *Mtb* complex. This is suggested by the very small probability of a gene of *Rv0080*'s size accumulating 5 or more amino acid altering mutations (this time considering both indels and non-synonymous SNPs) across the lineage defining branches of the *Mtb* complex phylogeny ($p=4x10^{-5}$; see **lines 477-480**). The nature of the specific mutations observed also lends evidence to our hypothesis, with the two frameshift-inducing indels mentioned, two in the inferred FAD-binding region and the remaining mutation predicted to have a strongly destabilising effect on protein structure (see **lines 455-471**). The fact *Rv0080* also experiences strong positive selection in the remaining two human-adapted lineages not to evolve a mutation on an upstream branch (L1 and L9), marks *Rv0080* as a gene subject to a truly singular pattern of positive selection. Therefore, while we acknowledge we lack data for specific lineage-defining point mutations like V60G and V31G, the above lines of evidence, and experimental data for the L121P, V49G and frameshift alleles renders enhancing hypoxia survival to be a strong and well-justified prior hypothesis as to their function.
